# Selective Small‐Molecule AdipoR1 Agonist 3‐Hydroxy Pterocarpan Salt (CDRI‐1709S) Ameliorates Skeletal Muscle Atrophy

**DOI:** 10.1002/jcsm.70328

**Published:** 2026-07-21

**Authors:** Md. Rameez Moin, Shyamal Pal, Shubhrajyoti Das, Pallavi Awasthi, Anushka Talukdar, Akhilesh Kumar, Shruti Suhas Varode, Nikhil Nikolas, Shamima Khatoon, Madhav Nilkanth Mugale, Rajdeep Guha, Sabyasachi Sanyal, Atul Goel

**Affiliations:** ^1^ Division of Biochemistry and Structural Biology CSIR‐Central Drug Research Institute Lucknow India; ^2^ Division of Medicinal & Process Chemistry CSIR‐Central Drug Research Institute Lucknow India; ^3^ Academy of Scientific and Innovative Research (AcSIR) Ghaziabad India; ^4^ Division of Toxicology and Experimental Medicine CSIR‐Central Drug Research Institute Lucknow India; ^5^ Laboratory Animal Facility CSIR‐Central Drug Research Institute Lucknow India

**Keywords:** adiponectin, AdipoR1 agonist, muscle fibre‐type, muscle function improvement, skeletal muscle atrophy

## Abstract

**Background:**

Skeletal muscle atrophy is a frequent comorbidity of metabolic disorders and chronic diseases, and despite its high prevalence, no pharmacological therapy is available, representing a major unmet clinical need. Adiponectin and its receptors are key regulators of skeletal muscle metabolism, mitochondrial function and myogenesis, yet clinical translation has been hindered by the lack of receptor‐selective agonists with favourable pharmacological and safety profiles. Here, we report the identification and characterization of CDRI‐1709S, the first small‐molecule AdipoR1‐selective agonist and evaluate its myogenic and anti‐atrophy efficacy.

**Methods:**

A PGC‐1α luciferase reporter‐based screen in AdipoR1/AdipoR2‐transfected, AdipoR‐low HEK293T cells identified CDRI‐1709S as an AdipoR1 agonist. Adiponectin‐associated signalling events were evaluated by immunoblotting in AdipoR1/2‐overexpressing HEK293T cells and AdipoR‐abundant C2C12 myotubes, with receptor specificity confirmed using RNA interference. Myogenic potential was assessed by morphometric analysis and immune detection of myogenic factors. Fibre‐type composition and metabolic capacity were evaluated using immunoblotting and extracellular flux analysis. Anti‐atrophy effects were examined in vitro using various assault‐induced models of myotube atrophy, and in vivo using rat models of dexamethasone (Dex) and sciatic nerve denervation‐induced muscle atrophy.

**Results:**

CDRI‐1709S selectively activated AdipoR1 with high potency (EC_50_: 414.7pM) and, at a pharmacologically relevant concentration (100 nM), induced rapid adiponectin‐associated signalling, including phosphorylation of AMPK, AKT and p38‐MAPK, along with upregulation of its downstream skeletal muscle metabolic targets PGC‐1α, GLUT4 and UCP3 in an AdipoR1‐dependent manner (*p* < 0.05). CDRI‐1709S promoted C2C12 myoblast differentiation into mature myotubes, accompanied by increased expression of MyoD and myogenin (*p* < 0.05). Treated myotubes were protected against cytokine‐, Dex‐ and nutrient‐deprivation‐induced atrophy through suppression of atrogenes Atrogin‐1 and MuRF‐1 (*p* < 0.01), restoration of myogenic markers (*p* < 0.05) and prevention of Dex‐induced fibre‐type switching toward glycolytic MyHC‐IIB, with concomitant induction of slow (MyHC‐I) and fast (MyHC‐IIA) oxidative fibres (*p* < 0.05). CDRI‐1709S also reversed Dex‐mediated impairments in oxidative and glycolytic capacity (*p* < 0.05).

Oral administration of CDRI‐1709S (10 mg/kg/day) in Dex‐ and denervation‐induced rat models restored atrogene expression, myogenic markers, local adiponectin signalling and myofibrillar architecture to normalcy (*p* < 0.05 to *p* < 0.0001). CDRI‐1709S prevented Dex‐induced enrichment of glycolytic fibres and preserved oxidative fibre composition (*p* < 0.05). The structural/molecular improvements translated into significant functional enhancements, including toe‐spread reflex in denervated limbs (*p* < 0.05) and increased grip strength (*p* < 0.0001) plus prolonged wire‐hang duration (*p* < 0.01) in Dex‐treated animals.

**Conclusion:**

CDRI‐1709S is the first AdipoR1‐selective small‐molecule agonist that induced myogenesis and robustly ameliorated skeletal muscle atrophy, establishing the proof‐of‐concept for AdipoR1‐targeting as a promising therapeutic strategy for sarcopenia and skeletal muscle atrophy.

## 1 Introduction

Adiponectin constitutes ~0.05% of the total serum protein [1] [S1]. Full‐length adiponectin, consisting of a collagenous and a globular domain, exists in three multimeric forms: trimeric, hexameric and high‐molecular‐mass [2] [S2 and S3], and is cleaved by leucocyte elastases to form the shorter bioactive globular adiponectin (gAd) [3, 4, 5] [S4]. gAd remains the most studied form and shows impressive pre‐clinical efficacy against metabolic disorders [6] [S2]. AdipoR1, AdipoR2 and T‐cadherin are the primary adiponectin receptors, with AdipoR1 and AdipoR2 being responsible for most of its reported biological activities, including regulation of energy metabolism, inflammation and mitochondrial function [2] [S5]. AdipoR1 is ubiquitously expressed, with the highest abundance in skeletal muscle; AdipoR2, although primarily expressed in the liver, is also present in skeletal muscle [2, 6].

Skeletal muscle serves as a key metabolic site and an endocrine organ mediating multi‐organ cross‐talk via synthesis and secretion of various myokines including adiponectin [7, 8, 9, 10] [S6]. Muscle atrophy occurs as a co‐morbidity of multiple metabolic disorders, anorexia, disuse, adverse effects of various drugs and ageing [11, 12] [S7 and S8]. Irrespective of the causes, muscle atrophy presents itself with enhanced muscle protein degradation consequent to upregulation of a cluster of genes commonly termed as atrogenes, which include the E3‐ubiquitin ligases MuRF1 and Atrogin‐1 [8, 11, 13]. In several pathologies including chronic heart failure, kidney and liver disease, a bidirectional relation exists between these organs and muscle atrophy, creating a vicious cycle [14, 15, 16].

Despite its wide impact, no pharmacotherapy is available [12] [S9]. Exercise and nutrition remain the standard‐of‐care option, which may be unfeasible for the elderly or bed‐ridden [17] [S10]. While a number of molecules targeting various pathways are under evaluation for muscle atrophy, the initial results are not encouraging, chiefly due to lack of functional improvement and cardiovascular liabilities [18, 19] [20, 21] [S9, S11].

Systemic hypoadiponectinemia is clinically associated with a plethora of metabolic disorders; however, a consensus for its correlation with skeletal muscle atrophy/sarcopenia has not been established, primarily due to insufficient delineation of the roles of different Adiponectin oligomers, autocrine and paracrine roles of muscle‐derived adiponectin and muscular adiponectin‐resistance under various pathological conditions [4, 22] [S4,S12,S13]. Despite the lack of clinical clarity, a large body of preclinical work, using adiponectin/adiponectin mimetics and transgenic systems, demonstrates that adiponectin signalling is not only crucial for energy metabolism, but also plays important roles in all facets of myogenesis, anti‐atrophic functions, endurance and imparting exercise benefits on skeletal muscle [4, 23, 24, 25, 26, 27] [S13–S20].

Despite its documented importance, adiponectin supplementation has not been possible owing primarily to its large size, which necessitates the discovery of small‐molecule AdipoR agonists. To date, five such molecules have been reported. GTDF, an isoflavone‐C‐glucoside [28] [S21], could not be translated due to problems in scalable synthesis, and AdipoRon, although widely studied, shows activity at high micromolar concentrations, unfavourable pharmacokinetics and toxicity [20, 29]. Isovitexin and ESME (AdipoR2‐specific), and AdipoAI (dual agonist) remain to be fully characterized [21, 30, 31]. Despite limitations, all these compounds exhibit impressive pre‐clinical efficacy against various pathophysiologies, and the myogenic and anti‐atrophy effects of AdipoRon and GTDF are well established [26, 28, 32, 33] [S14, S22].

Together, pharmacological intervention in skeletal muscle atrophy is a critical unmet clinical need, and given the crucial role adiponectin and its receptors play in every facet of muscle biology, small‐molecule AdipoR agonists, with favourable efficacy, pharmacological and toxicity profiles, may provide promising therapeutic options for this condition.

Here we report the discovery and characterization of CDRI‐1709S (1709), a previously described synthetic osteogenic pterocarpan [34], as a small‐molecule selective AdipoR1 agonist and demonstrate its myogenic and anti‐atrophy efficacy.

## 2 Materials and Methods

### 2.1 Chemicals and Reagents

CDRI‐1709S (1709) was synthesized as described in Supporting Information S1: Supplementary Methods and Full blots. gAd was synthesized and purified as before [22]. Cell‐culture reagents were from Gibco and Sigma‐Aldrich. Fine chemicals: Sigma‐Aldrich, Abcam, Cayman, BioVision and MP Biomedicals (Navi Mumbai, India). Stock solutions of Forskolin and all the inhibitors were prepared in cell culture‐grade DMSO. gAd was dissolved in sterile PBS (stock: 1 mg/mL). Compound 1709 was dissolved in dH2O. Final conc. of DMSO and PBS in all experiments was 0.1% and 0.001% respectively. Different vehicles were compared in pilot experiments, and since no difference was observed, DMEM‐high glucose (DMEM‐HG; the final diluent) was used as vehicle. Antibody information: Table S1.

### 2.2 Cell Culture

C2C12 (CRL‐1772) and HEK293T (CRL‐3216) were from ATCC and were cultured in growth medium (GM: DMEM‐high glucose (HG) + 10% FBS (#16000044)) with 1% antibiotic‐antimycotic (Gibco) at 37°C and 5% CO2. C2C12 cells were used between 2nd and 10th passages. HEK293T cells: 10–18th passages.

#### 2.2.1 Myogenesis Induction in C2C12 Myoblasts

90%‐confluent C2C12 myoblasts were treated with serum‐free DMEM‐HG medium (SS) supplemented with 1709/gAd/AdipoRon. The SS‐treated and differentiation medium (DM; DMEM‐HG + 2% Horse Serum (HS))‐treated cells served as negative and positive controls, respectively. Compound treatments were replenished every 24 h. Phase contrast images were captured on the 3rd day of treatment.

#### 2.2.2 Luciferase Reporter Assays

Luciferase assays were performed as earlier [21]. Briefly, HEK293T cells on 24‐well plates were transfected as indicated using Lipofectamine 3000 (Invitrogen). Twenty‐four hours after transfection, cells were treated as indicated. Green fluorescence (from the transfected eGFPN1 plasmid) and luciferase activity were determined in a plate reader (SpectraMax M series) and a luminometer (Glomax, Promega), respectively.

#### 2.2.3 AdipoR1/R2 Overexpression and Assessment of Rapid Signalling Events

HEK293T cells seeded in 6‐well plates were transfected as above. Twenty‐four hours post transfection were treated as indicated and analysed by immunoblotting.

#### 2.2.4 Generation of Lentivirus in HEK293T

Lentiviral particles containing sh‐Scr (scramble), sh‐AdipoR1 or sh‐AdipoR2 were packaged in HEK293T cells using standard methods.

#### 2.2.5 Knockdown of AdipoR1 or AdipoR2 in C2C12 Myotubes

Differentiated C2C12 myotubes in 6‐well plates were infected with lentivirus soup. Forty‐eight hours later, medium was replaced and myotubes were treated as indicated, harvested and lysates were used for immunoblotting. Mouse shRNAs were from Sigma‐Aldrich in pLKO.1 vector. AdipoR1: TRC Clone ID‐TRCN0000249149 (set‐1); Target sequence: GGGATTGCTCTACTGATTATG, TRC Clone ID‐TRCN0000257836 (set‐2); Target sequence: AGATGGAGGAGTTCGTGTATA. AdipoR2: TRC Clone ID‐TRCN0000175771 (set‐1); Target sequence: GCAGGAATTTCGTTTCATGAT; TRC Clone ID‐TRCN0000173201 (set‐2) Target sequence: CCATCATGCTATGGAACGAAT. Packaging plasmids psPAX2 (#12260) and pMD2.G (#12259) were from Addgene.

### 2.3 Immunofluorescence (MyHC Staining) of C2C12 Myotubes

C2C12 myoblasts seeded on sterile coverslips in 12‐well plates were treated as indicated, fixed using 10% Neutral buffered formalin (NBF) followed by permeabilization with 1% TritonX‐100 (Sigma‐Aldrich), blocked with 1% BSA Fraction V (MP Biomedicals) and probed with MyHC antibody (Clone MF20, Developmental Studies Hybridoma bank) followed by incubation with Alexa Fluor‐488‐conjugated secondary antibody (Invitrogen). Nuclei were stained with DAPI‐containing mounting medium (Abcam). Images were captured and morphometry was performed using ImageJ [S23].

### 2.4 Inhibitor Studies in Myoblasts and Myotubes

90% confluent C2C12 myoblasts were pre‐treated for 30 min with Compound C (20 μM; Abcam)/Wortmannin (1 μM; Sigma‐Aldrich)/SB203580 (10 μM; Sigma‐Aldrich), followed by 3‐day treatment with SS, 2% HS or SS + 1709/gAd. For evaluating inhibitors on amelioration of Dexamethasone (Dex)‐induced atrophy by 1709, fully differentiated myotubes were treated with inhibitors for 30 min followed by treatment with Dex ± 1709 for 48 h.

### 2.5 In Vitro Myotube Atrophy Models

C2C12 myotubes were pre‐treated for 24 h with SS or SS + 1709/gAd/AdipoRon, washed with PBS, followed by 6‐h nutrient deprivation in PBS (±Ca2^+^) with or without 1709/gAd/AdipoRon. For Dex‐ or LT‐induced atrophy models, myotubes were pre‐treated as above, followed by 48 h treatment with Dex (10 μM), or LT (1 μg/mL Lipopolysaccharide (LPS) (Invivogen) + 20 ng/mL Tumour necrosis factor alpha (TNFα) (BioVision)) ± 1709 or other AdipoR agonists. Treatments were replenished every 24 h. Phase contrast images were captured and analysed by ImageJ.

### 2.6 Fatty Acid Oxidation Assay

C2C12 myoblasts (80 000 cells/well) in 24‐well XF cell plates (Agilent) were differentiated for 4 days in DM. Myotubes once formed were pre‐treated with vehicle/1709/gAd for 24 h, followed by treatment with Dex (10 μM) ± test compounds for 48 h. Myotubes were then serum starved for 6 h followed by incubation in a non‐CO2 incubator at 37°C for 45 min in Krebs–Henseleit buffer (KHB) supplemented with 1 mM glutamine (Gibco) and 0.5 mM L‐carnitine (Cayman). Among the four ports on XFe cartridge, 1.5 μM of FCCP (Abcam) was supplied to the 1st port, and the others were left empty. Cartridge plate was inserted in the Seahorse XFe24 Analyser (Agilent) for a 12 min equilibration and calibration. Just prior to loading the XF cell plate into the machine, 250 μM palmitate (Sigma‐Aldrich) or 38 μM BSA (MP Biomedicals) was added to corresponding wells and the oxygen consumption rate (OCR) and extracellular acidification rate (ECAR) were measured, normalized with protein concentration, plotted against time (min) and expressed as pmol/min/μg protein. This experiment also included another treatment group corresponding to Medicarpin; a compound we identified as a dual AdipoR1/R2 agonist, which is being reported separately, and we thus declare that both this and the Medicarpin study share the same V, Dex and Dex + gAd groups pertaining to the extracellular flux analysis.

### 2.7 In Vivo Experiments

All experiments were approved by the Institutional Animal Ethical Committee (IAEC) (approval No.: IAEC/2022/44). Outbred Sprague Dawley (SD) rats were obtained from the National Laboratory Animal Center at CSIR‐CDRI, and caged at 24°C ± 2°C; humidity 50%–60%; with 12‐h light/dark cycles (light: 300 Lux). Rats had ad libitum access to dH2O and normal chow (Altromin), and were acclimatized for 7 days before the experiment. Termination involved euthanizing with three times the anaesthesia dose of Ketamine and Xylazine (anaesthesia dose: Ketamine (80 mg/kg) + Xylazine (10 mg/kg)).

#### 2.7.1 Dexamethasone‐Induced Muscle Atrophy

Before the start of the experiments, female SD rats (8‐week‐old; body weight 255 ± 20 g) were trained on a wire hanging apparatus, grip‐strength monitor and rotarod for 4 days. Rats were then randomized (body weight‐based) into three groups (*n* = 6/group): Dex (200 μg/kg/day i.p.), Dex + 1709 (Dex + 1709: 10 mg/kg/day/p.o. in distilled water) and Vehicle (V) group (orally fed distilled water) and dosed for 15 days. Body composition was assessed by EchoMRI on day 0, 7 and 14 (details in Data S1). Functional studies were performed on day 15. Sacrifice and tissue harvesting were conducted on day 16. Prior to sacrifice, blood samples were collected via the retro‐orbital route. Serum adiponectin levels were measured using a Rapid ELISA kit (Thermo‐Fisher Scientific). Gastrocnemius (GN) muscles from the left hindlimbs were snap‐frozen and stored at −80°C whereas those from right hindlimbs were fixed using 10% NBF. This experiment also included the Medicarpin treatment group, which is being reported separately, and we thus declare that both this and the Medicarpin study share the same V and Dex groups pertaining to the Dex‐induced rat muscle atrophy experiments.

##### 2.7.1.1 Functional Tests

All animals were trained and acclimatized on a wire hang setup, grip strength monitor and rotarod instrument (three sessions/day; 15‐min intervals in between). On the final test day, three sessions were conducted for each functional test with 15‐min breaks between each session.

###### 2.7.1.1.1 Wire Hang Test

A 1 mm thick wire, under tension, was set horizontally 50 cm above the floor. Rats were trained to grip and hang onto the wire, using forepaws only. On the test day, muscular strength was assessed by the following scoring system: 0 = failure to hang, 1 = hanging with both forelimbs, 2 = hanging with both forelimbs and climbing on wire, 3 = hanging with both fore‐ and hind limbs, 4 = hanging with fore and hindlimbs with tail wrapped around the wire, 5 = attempting to escape after climbing on the wire with all four limbs.

###### 2.7.1.1.2 Grip‐Strength Analysis

Rats were trained to use both forelimb paws to grip onto the steel grid of the grip‐strength meter (Chatillon, DFE2–010). On test day, scores were recorded (three readings at 15‐min interval).

###### 2.7.1.1.3 Rotor‐Rod Test

Rats were trained to walk on a rotarod machine (IITC Inc./Life Science); among the different rpms of rotarod tested (5–30 rpm, 5 min each), 15 rpm, 5 min was selected and used on the test day.

#### 2.7.2 Denervation‐Induced Atrophy

Male SD rats (8‐week‐old; body weight 250 ± 20 g) were randomly divided into two groups (*n* = 6/group). Right hindlimbs of one group served as Sham (skin opened, sciatic nerve exposed, sutured back without transection), while left hindlimbs were denervated (a 5 mm section of sciatic nerve was removed and nerve ends were ligated); these left hindlimbs served as the Den group. This group was orally dosed with distilled water and served as the Sham and Den groups. One animal from the 7‐day Sham/Den group died on the 4th day of the experiment. Hence, in the 7‐day experiment, *n* = 5 for Sham/Den. Left hindlimbs of rats from the other group (*n* = 6) were denervated and served as the Den+1709 group. Twenty‐four hours post‐surgery, the rats received oral dosing of distilled water (Sham/Den) or 1709 (Den+1709, 1709: 10 mg/kg/day) for 3 or 7 days. Rats were then euthanized; GN muscles were collected, halved vertically; one half was snap frozen and stored at −80°C, the remaining half was stored in 10% NBF for histology.

##### 2.7.2.1 Toe Spread Scoring in Denervation‐Induced Atrophy Model

For toe‐spread analysis [17], hind paws were dipped in a non‐toxic dye and gently touched onto filter paper without forceful pressing and scored using a three‐point scoring system (0 = no spreading of the toes, 1 = intermediate spreading, 2 = complete spreading).

#### 2.7.3 H&E and WGA Staining of Transverse Muscle Sections

Muscles fixed in 10% NBF were cut into transverse sections (7 μm) and placed on Poly‐L‐Lysine‐coated slides and stained with Haematoxylin & Eosin (H&E) or wheat germ agglutinin (WGA). For WGA staining, sections were permeabilized with 0.1% TritonX‐100, followed by incubation with WGA (Alexa Fluor‐488‐conjugated, 5 μg/mL in PBS) for 2 h. The nuclei were stained with DAPI in mounting medium.

#### 2.7.4 Myofibre Quantification

Cross‐sectional area and Feret's diameter were calculated using ImageJ [S23]. All myofibres with clear and distinct boundaries from each image were analysed for unbiased myofibre size assessment.

### 2.8 Immunoblotting

Cells/tissue were lysed in chilled RIPA buffer with protease and phosphatase inhibitors (Sigma‐Aldrich). Equal amounts of proteins were resolved by SDS‐PAGE, blotted onto PVDF membranes (Millipore), probed with appropriate primary and HRP‐conjugated secondary antibodies, and chemiluminescence was detected using an ECL kit (Millipore) in a LAS4010 chemidoc (Roche). Full immunoblots are included in Supporting Information S1: Supplementary Methods and Full blots.

### 2.9 Quantitative Real‐Time Polymerase Chain Reaction (QRT‐PCR)

Detailed methods for QRT‐PCR are in Data S1 and Table S2.

### 2.10 Statistical Analysis

Graphs are Mean ± SEM (*n* = 3, or as indicated). One‐way or two‐way ANOVA followed by Bonferroni's post‐test, or Kruskal–Wallis test followed by Dunn's post‐test, as appropriate were used for statistical analysis. For checking intra‐group variances, Bartlett's test was used. Statistics involving two groups were performed by unpaired, two‐tailed Student's *t*‐test (F test used to assess intra‐group variance). GraphPad Prism 5 was used to analyse data; *p < 0.05, **p < 0.01, ***p < 0.0001 were considered statistically significant.

## 3 Results

### 3.1 CDRI‐1709S is a Selective Agonist for AdipoR1

**FIGURE 1:**
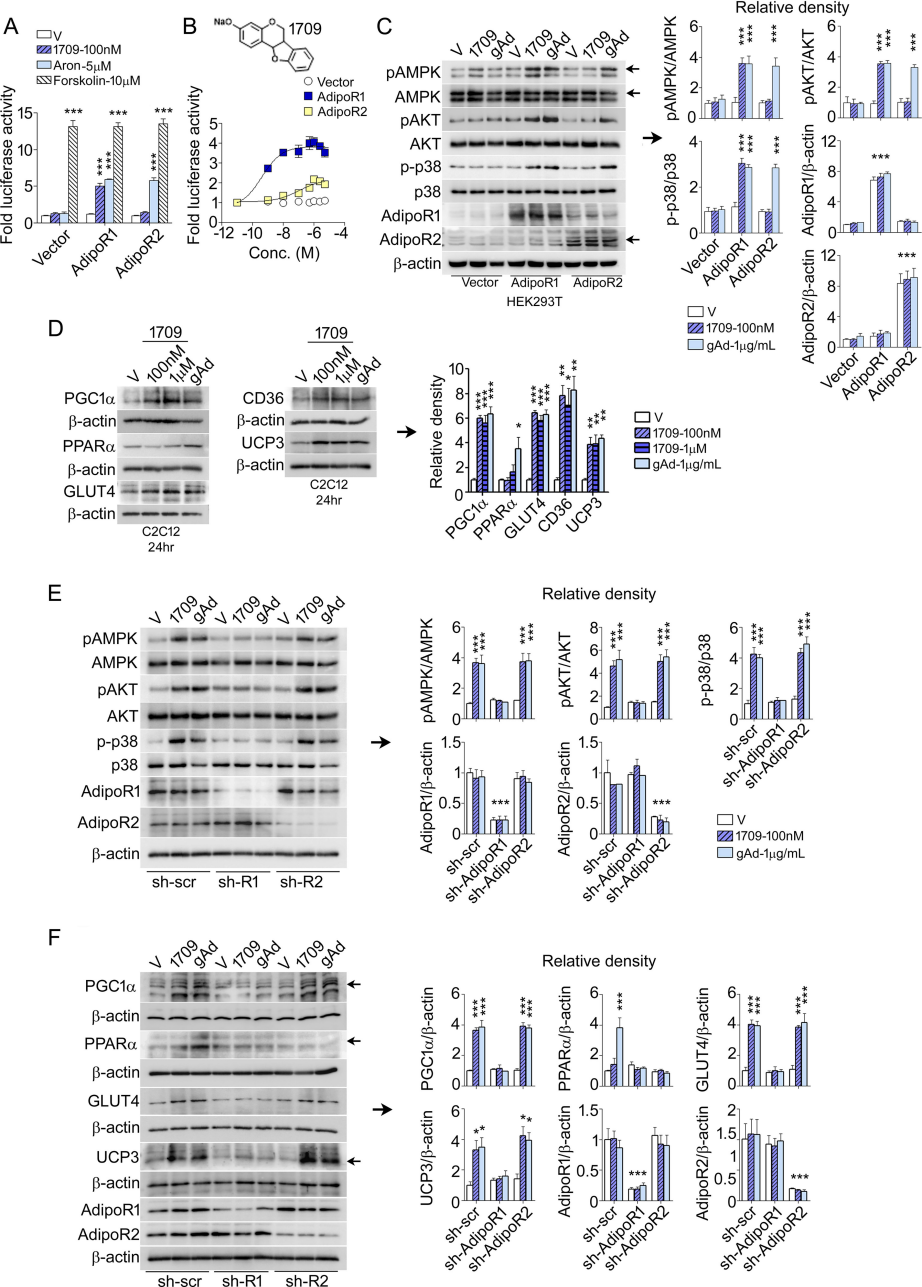
CDRI‐1709S activated adiponectin signalling via AdipoR1 but not AdipoR2. (A, B) Compound 1709 induced PGC‐1α‐luc reporter only in AdipoR1 but not empty vector or AdipoR2 transfected HEK293T cells. (A) HEK293T cells transfected with ~1.1 kb PGC‐1α‐luciferase reporter (400 ng/well) and empty vector/AdipoR1/AdipoR2 (400 ng/well) expression vectors and eGFPN1 (200 ng/well, normalizing control), were treated with vehicle (V) or test compounds (1709: 100 nM, AdipoRon: 5 μM or Forskolin: 10 μM) for 24 h. Luciferase activity was measured and normalized with green fluorescence (emitted by eGFPN1 plasmid) (*n* = 3, in triplicates). (B) For EC50 determination, HEK293T cells were transfected as described above, and treated for with different concentrations of 1709 for 24 h followed by plotting of Luciferase/green fluorescence values (*n* = 3, in duplicates). (C) CDRI‐1709S induced adiponectin‐associated rapid signalling events only in AdipoR1 overexpressing HEK293T cells. HEK293T cells were transfected as above (but without PGC‐1α‐luc plasmid) with 2000 ng/well of empty vector/AdipoR1/AdipoR2 expressing plasmids and eGFPN1 (500 ng/well) followed by treatment with V/1709 (100 nM)/gAd (1 μg/mL) for 10 min. Protein expression was analysed by western blotting; adjacent graphs represent densitometry (*n* = 3). (D–F) CDRI‐1709S activated adiponectin signalling and expression of its downstream targets via AdipoR1, but not AdipoR2. (D) C2C12 myotubes were treated with V, 1709 (100 nM/1 μM) or gAd (1 μg/mL) for 24 h and protein expression analysed by immunoblotting (*n* = 3). In all subsequent experiments, the concentrations of 1709, gAd and AdipoRon used were 100 nM, 1 μg/mL and 5 μM, respectively (unless mentioned otherwise). (E, F). C2C12 myotubes were infected with sh‐scr/shAdipoR1/shAdripoR2‐lentivirus. Forty‐eight hours post‐infection, cells were treated for 10 min (E) or 24 h (F) and protein expression analysed by immunoblotting. Adjacent graphs represent densitometries. Data represented as mean ± SEM (*n* = 3). For densitometry, phosphoproteins were normalized with their respective total proteins; for other proteins, β‐actin was used for normalization; stripping and reprobing were done on the same blots. Statistical analyses were performed by 1‐way (D) or 2‐way ANOVA (A, C, E, F) followed by Bonferroni's post‐test. *V/vector/sh‐scr vs. treatment groups/AdipoRs/sh‐AdipoRs. * *p* < 0.05, ** *p* < 0.01, *** *p* < 0.0001. Aron; AdipoRon, gAd; globular adiponectin.

Adiponectin promotes osteoblast differentiation and inhibits osteoclastogenesis [24, 35] [S24], and osteogenic compounds like GTDF and isovitexin were later identified as AdipoR agonists [21, 28]. We thus screened an in‐house library of osteogenic compounds (screening conc. 100 nM) for their ability to modulate AdipoRs using a recently established assay [21], and 1709 was found to activate a PGC‐1α promoter‐driven luciferase reporter in AdipoR‐low HEK293T cells transfected with AdipoR1, but not AdipoR2 (Figure 1A). gAd (1 μg/mL), which activates both receptors, increased reporter activity in the presence of either receptor, while forskolin action was AdipoR‐independent (Figure 1A). AdipoR1 was activated by 1709 with an EC_50_ of 414.7pM (Figure 1B). The reported Cmax of 1709 in SD rats is ~120 nM [34]; therefore, a pharmacologically relevant 100 nM and a suprapharmacological 1 μM concentrations were used for subsequent studies. Treatment with 1709 for 10 min induced AMPK, AKT and p38‐MAPK phosphorylations in AdipoR1‐transfected HEK293T cells (Figure 1C), and endogenous AdipoR‐rich C2C12 myotubes (Figure S1). Treating C2C12 myotubes with 1709 for 24 h increased expression of canonical adiponectin targets, including PGC‐1α, GLUT4, CD36 and UCP3 (Figure 1D). In contrast, PPARα, a known AdipoR2‐specific target [36], was induced by gAd but not 1709 (Figure 1D).

To confirm receptor selectivity, we performed AdipoR1/AdipoR2 knockdown in C2C12 myotubes using two independent shRNA sets and assessed 1709‐induced signalling events. AdipoR1 but not AdipoR2 depletion abrogated 1709‐induced AMPK, AKT and p38 phosphorylation (Figure 1E, Figure S2). Similarly, PGC‐1α, GLUT4 and UCP3 induction by 1709 was compromised only in AdipoR1‐depleted cells (Figure 1F, Figure S2). Interestingly, despite PPARα being an AdipoR2 target, gAd‐induced PPARα expression was reduced when either receptor was depleted (Figure 1F, Figure S2).

These results establish 1709 as a selective AdipoR1 agonist that activates classical adiponectin‐associated signalling pathways.

### 3.2 CDRI‐1709S Induced Myogenesis in C2C12 Myoblasts

**FIGURE 2:**
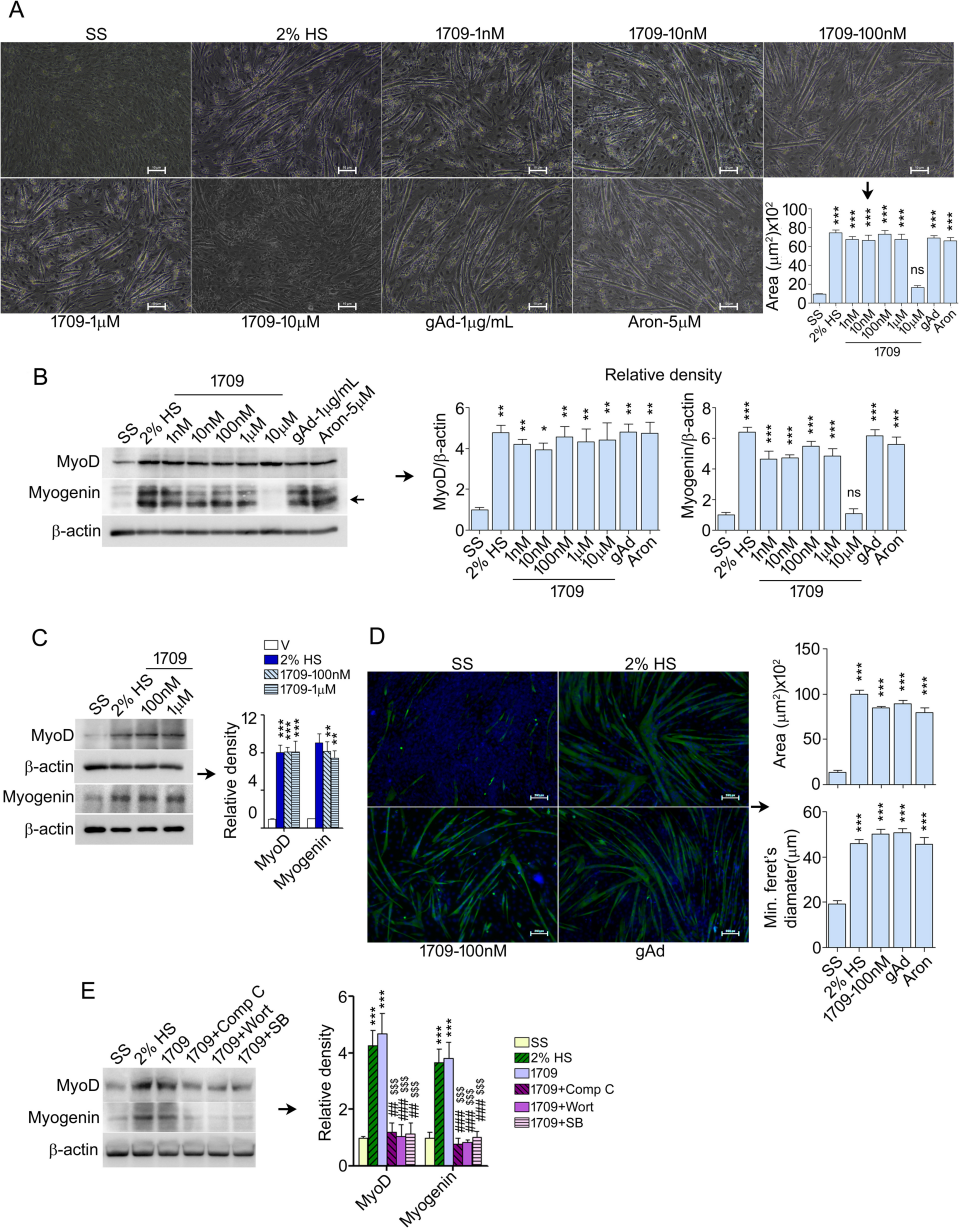
CDRI‐1709S induced differentiation of C2C12 myoblasts to myotubes. (A) Representative of phase‐contrast images comparing differences in differentiation following 3rd day treatment of 90% confluent myoblasts, with 1709 (different indicated conc.), gAd or AdipoRon in SS (serum‐free DMEM‐HG media). 2% HS treatment served as positive control. These phase‐contrast images were captured on Nikon ECLIPSE Ts2 microscope (magnification 100×; scale bar 10 μm). Adjacent graph represents myotube area (*n* = 3, 18 images/treatment group) (B, C) Expression of MyoD and myogenin (myogenesis markers) was assessed by densitometry of immunoblots. C2C12 myoblasts were treated with incremental concentrations (B) or 100 nM (**C**) of 1709.2% HS, gAd or AdipoRon were used as controls. Right panel represents densitometry. (D) Representative images of Immunofluorescence (IF) stained cells for myosin heavy chain (MyHC) (MF‐20 antibody). Here, green florescence: MyHC expression, blue fluorescence: DAPI stained nuclei. IF images were captured on Nikon ECLIPSE Ts2 microscope (magnification 100×; scale bar 10 μm). Right panel: morphometric comparison of Area and Min. Feret' diameter (*n* = 3, 18 images/treatment group). (E) Assessment of 1709‐induced differentiation in presence of various inhibitors. Myoblasts were pre‐treated for 30 min with Compound C (20 μM)/Wortmannin (1 μM)/SB 203580 (1 μM) followed by treatment with 1709 or gAd. SS only and SS + 2% HS treatments served as negative and positive controls respectively. Treatments were replenished every 24 h. Right panel: quantification of MyoD and myogenin expression; analysed by immunoblotting. All morphometry and densitometry performed using ImageJ and graphs, respectively, represent mean ± SEM (*n* = 3). *SS vs. treatment groups, #HS vs. treatment groups, $: 1709 vs. 1709 + inhibitors (A–E) Statistical analyses were performed by 1‐Way ANOVA followed by Bonferroni's post‐test. */#/$ *p* < 0.05, **/##/$$ *p* < 0.01, ***/###/$$$ *p* < 0.0001. Comp C: Dorsomorphin, SB: SB 203580, Wort: Wortmannin.

Adiponectin and its mimetics are established inducers of myogenesis [26, 27] [S17, S20]. We therefore examined the myogenic potential of 1709 in confluent C2C12 myoblasts cultured in serum‐free medium. In a dose–response study, 3‐day treatment with 1709 induced differentiation, characterized by elongated myotube formation from 1 nM onwards and this effect was lost at 10 μM (Figure 2A). Consistently, expression of the early myogenic marker MyoD and the late marker myogenin was increased by 1709 (1 nM‐1 μM), gAd/AdipoRon (Figure 2B). Notably, although 1709 at 10 μM induced MyoD, it failed to upregulate myogenin (Figure 2B), suggesting a biphasic effect at higher concentration. Similar findings are reported for AdipoRon depicting high‐dose AdipoRon toxicity and superior efficacy at lower doses in muscle [33]. The myogenic activity of 1709 was again confirmed in C2C12 myoblasts treated with 100 nM 1709, with resultant induction of MyoD, myogenin and myosin heavy chain (MyHC) (Figure 2C,D).

To determine which of the adiponectin‐associated early signalling events is involved in 1709‐induced myogenesis, myoblasts were treated with inhibitors of AMPK (Compound C), AKT (wortmannin) or p38‐MAPK (SB203580) prior to 1709 treatment, and each of these inhibitors completely blocked 1709‐induced MyoD/myogenin expression and myotube formation (Figure 2E), indicating that AMPK, AKT and p38‐MAPK signalling are all essential for 1709‐mediated myogenesis.

### 3.3 CDRI‐1709S Ameliorates C2C12 Myotube Atrophy

**FIGURE 3:**
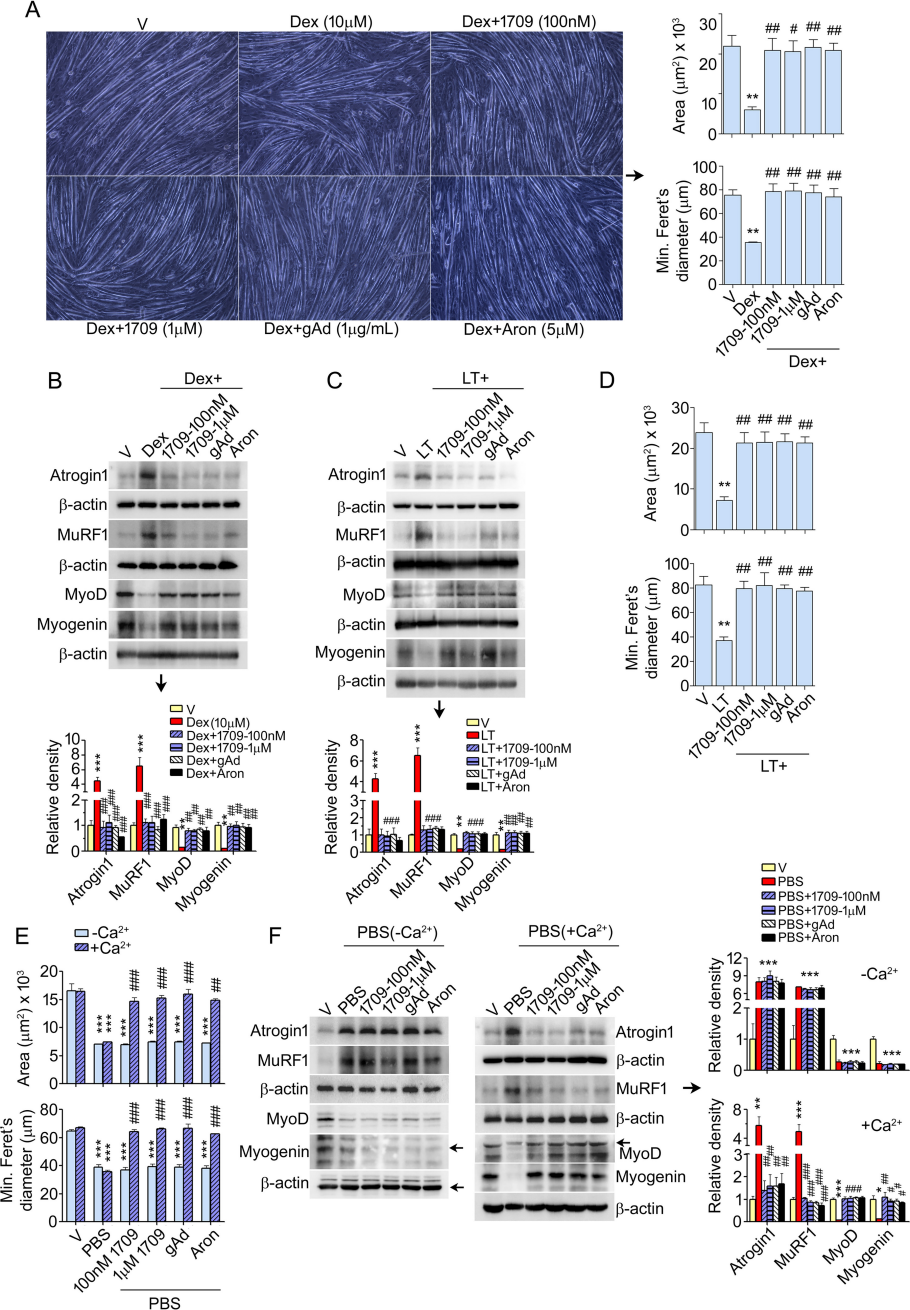
CDRI‐1709S protected myotubes against Dexamethasone, LPS + TNFα and PBS (nutrient deficiency)‐induced atrophy. (A, B) Compound 1709 protected against Dex‐induced myotube atrophy. C2C12 myotubes were pre‐treated with 1709/gAd/AdipoRon for 24 h in a serum free medium and then treated with Dex (10 μM) alone or Dex + 1709/gAd/AdipoRon for 48 h. Treatments were replenished every 24 h. Left panel: Phase contrast images of myotubes. Right panel: quantification of area and min. Feret's diameter of myotubes (*n* = 3, 18 images/treatment group). (B) Protein expression analysed by immunoblotting and quantified using densitometry (lower panel). (C, D) Compound 1709 protected against LT‐induced myotube atrophy. C2C12 myotubes were pre‐treated with 1709/gAd/AdipoRon for 24 h in a serum‐free medium and then treated as indicated with LT or LT + indicated compounds. (C) Protein expression analysed by immunoblotting and quantified using densitometry (lower panel). (D) Area and Min. Feret's diameter of myotubes quantification using phase contrast images (representative images provided in Figure S4). (E, F) Compound 1709 protected myotubes from nutrient‐deficiency‐induced atrophy in the presence of Ca^2+^. C2C12 myotubes were pre‐treated with 1709/gAd/AdipoRon for 24 h, in a serum‐free medium and then treated with 1709/gAd/AdipoRon in PBS (Ca^2+^ free or Ca^2+^ containing) for 6 h only. (E) Quantification of myotube area and min. Feret's diameter (corresponding representative phase‐contrast images provided in Figure S4). (F) Protein expression analysed using immunoblots. Right panel represents densitometry. All phase contrast images were captured on Leica DMI6000B (magnification 100×; scale bar: 200 μm). All data represented as mean ± SEM (*n* = 3). */# *p* < 0.05, **/## *p* < 0.01, ***/### *p* < 0.0001. *V vs. treatment groups, #Dex/LT/PBS vs. Dex/LT/PBS + 1709. Statistical analyses were performed by 1‐Way ANOVA followed by Bonferroni's post‐test.

We next assessed the anti‐atrophy potential of 1709 in C2C12 myotubes exposed to dexamethasone (Dex), inflammatory cytokines (LPS + TNFα; LT) or nutrient deprivation (PBS ± Ca2^+^). Fully differentiated myotubes pre‐treated with 1709, gAd/AdipoRon for 24 h, were exposed to atrophic stimuli. Dex and LT induced pronounced myotube shrinkage, reflected by reduced myotube area and diameter, along with induction of Atrogin‐1 and MuRF1, and suppression of MyoD and myogenin, and all these effects were reversed by 1709, gAd/AdipoRon (Figure 3A–D, Figures S3 and S4). The effects imparted by 1709 were also validated using QRT‐PCR in the Dex‐induced myotube atrophy model, and here as well, Dex induction of MuRF‐1 and Atrogin and suppression of Myogenin transcripts were reversed by 1709 treatment (Figure S3). In the nutrient‐deprivation model, PBS treatment induced myotube atrophy and atrogene expression regardless of the presence of Ca^2+^. However, the protective effects of 1709, gAd/AdipoRon were observed only in Ca^2+^‐containing PBS (Figure 3E,F, Figure S4). These results are consistent with canonical Adiponectin signalling via Ca^2+^‐dependent pathways [26, 37] and indicate that 1709 confers protection against muscle atrophy via typical adiponectin signalling mechanisms.

### 3.4 CDRI‐1709S Increased the Expression of Oxidative Fibre Type Markers and Oxidative Capacity of Myotubes

**FIGURE 4:**
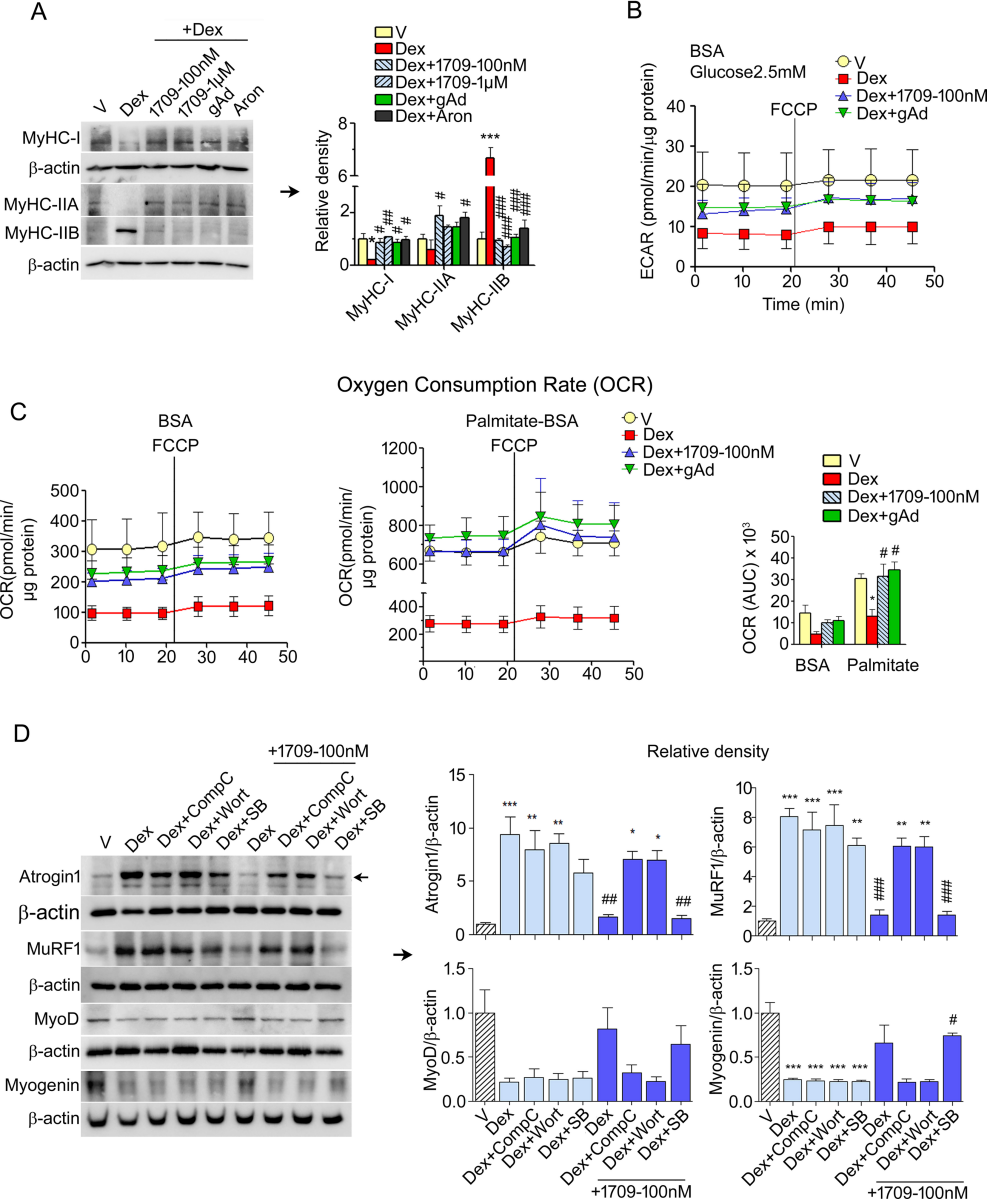
CDRI‐1709S increased the oxidative capacity of myotubes and attenuated Dex‐induced fibre‐type changes in C2C12 myotubes. (A) Dex induced expression of glycolytic MyHC‐IIB, and reduced oxidative MyHC‐I and IIA expression in C2C12 myotubes while 1709 reversed it. C2C12 myotubes were first pre‐treated with 1709/gAd/AdipoRon for 24 h and were then treated with Dex for 48 h. Left panel: immunoblots, Right panel: graphs representing densitometry. (B, C) Compound 1709 improved Dex‐induced reduction of glycolytic and FAO rate in C2C12 myotubes. C2C12 cells plated on a 24‐well Seahorse XFe cell culture plate (80 000 cells/well), differentiated for 4 days in DM, followed by treatment with Dex/Dex + 1709/Dex + gAd for 48 h. Next, ECAR (B) was measured in presence of 2.5 mM glucose and OCR (C) was measured using a real‐time treatment with 250 μM palmitate (right panel) or 38 μM BSA (vehicle; left panel) (*n* = 3, in triplicates). (D) Inhibition of AMPK and AKT compromised 1709‐induced amelioration of Dex‐induced atrogene expression. C2C12 myotubes were treated with Compound C/Wortmannin/SB 203580 for 30 min, followed by treatment with Dex/Dex + 1709 for 48 h. Protein expression was analysed by immunoblotting. Right panel: Graphs represent quantification of protein expression by densitometric analyses. Graphs corresponding to Western blots represent densitometric analyses: *n* = 3. */# *p* < 0.05, **/## *p* < 0.01, ***/### *p* < 0.0001. *V vs. treatment groups, #Dex vs. treatment groups. $Dex + 1709 vs. treatment groups. Statistical analyses were performed by 1‐Way ANOVA followed by Bonferroni's post‐test. ECAR: extracellular acidification rate, FCCP: carbonyl cyanide‐p‐trifluoromethoxyphenylhydrazone, MyHC: Myosin Heavy Chain, OCR: oxygen consumption rate.

PGC‐1α, a key adiponectin target, regulates mitochondrial biogenesis and muscle fibre‐type specification [25, 38]. Since corticosteroid‐derived atrophy preferentially affects fast glycolytic fibres [39] [S26], we examined fibre‐type markers in Dex‐, Dex + 1709‐ or Dex + gAd‐treated myotubes. Interestingly, Dex suppressed oxidative fibre markers (MyHC‐I and MyHC‐IIA) and induced the glycolytic marker MyHC‐IIB, whereas 1709 and gAd/AdipoRon reversed these changes (Figure 4A). Despite the shift towards glycolytic fibres, Dex‐treated myotubes showed reduced glucose utilization, reflected by decreased ECAR, and 1709 or gAd restored it (Figure 4B). Similarly, Dex suppressed mitochondrial oxidative capacity, particularly when palmitate was used as the energy source, while 1709 and gAd restored OCR to near‐control levels (Figure 4C). Pathway inhibition studies revealed that unlike in myoblast differentiation, where all three pathways were necessary (Figure 2E), AMPK and AKT, but not p38‐MAPK, were required for the anti‐atrophy effects of 1709 in Dex‐treated myotubes (Figure 4D).

### 3.5 CDRI‐1709S Prevented Dex‐Induced Skeletal Muscle Atrophy

**FIGURE 5:**
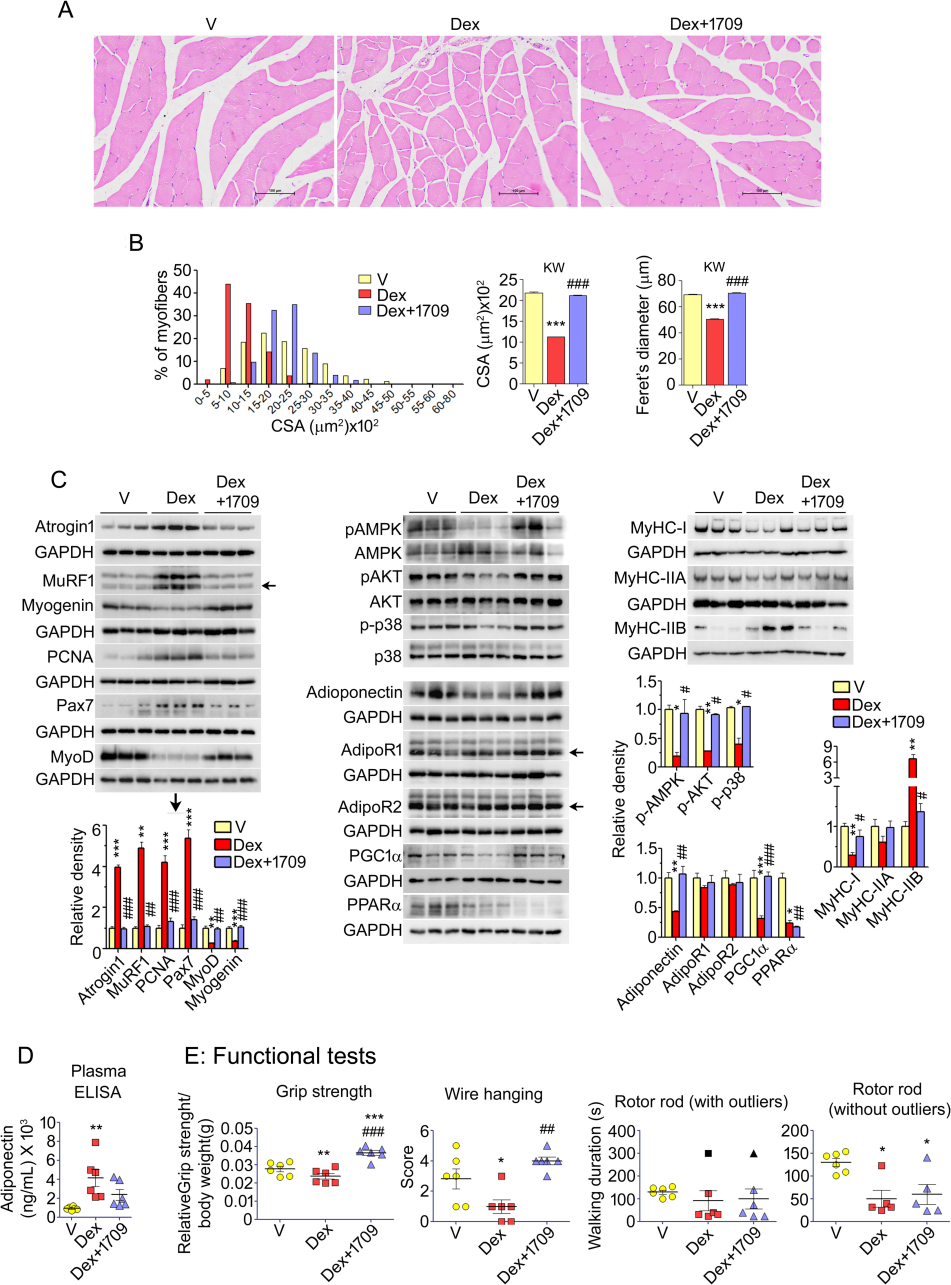
CDRI‐1709S prevented dexamethasone‐induced skeletal muscle atrophy in SD rats. Female SD rats were divided into 3 groups—V, Dex and Dex + 1709 (as described in Materials and methods). (A, B) Compound 1709 prevented Dex‐induced myofibre changes. (A) Representative images of H&E‐stained transverse sections of GN muscles; images captured using Nikon Eclipse E200 microscope (magnification 200×; Scale bar 100 μm). (B) Percentage of myofibre distribution based on cross‐sectional area (CSA). Graphs represent quantification of myofibre CSA and Feret's diameter. Quantification: 30 images/group, 6 animals/group. (C) Compound 1709 prevented upregulation of Dex‐mediated expression of atrogenes, suppression of adiponectin‐associated signalling events, changes in expression of fibre‐type markers, and also hastened muscle repair. Western blotting was performed to assess changes in protein expression of indicated proteins (in GN muscle). Each lane represents protein sample from a single animal. Immunoblots represent protein expression of three animals/group (immunoblots of samples from other three animals are provided in Supporting Information S1: full blots). Corresponding graphs represent quantification using protein expression of all six animals in each group. Phosphoproteins were normalized with their respective total proteins, for other proteins; β‐actin was used for normalization; stripping and reprobing done on same blots. (D) Analysis of plasma levels of adiponectin among indicated groups (*n* = 6/group). (E) Compound 1709 restored Dex‐mediated reduction in functional parameters in rats. Muscle function was evaluated using wire hanging, grip strength and rotarod tests (*n* = 6/group). All images and immunoblots were quantified using ImageJ. Data are presented as mean ± SEM (*n* = 6/group). */# *p* < 0.05, **/## *p* < 0.01, ***/### *p* < 0.0001. *Control vs. other groups, #Dex vs. Dex + 1709. Statistical analyses were performed by 1‐Way ANOVA followed by Bonferroni's post‐test (C: Atrogin1, PCNA, Pax7 and Myogenin, E: all functional tests except rotarod test without outlier) or Kruskal–Wallis test followed by Dunn's post‐test (C: all proteins except Atrogin1, PCNA, Pax7 and Myogenin, E: rotarod test without outlier).

To determine if the in vitro effects of 1709 could be translated in vivo, we evaluated its efficacy in a Dex‐induced systemic atrophy model in SD rats. Animals were treated with Dex (200 μg/kg/day, i.p.), Dex + 1709 (10 mg/kg/day, oral) or vehicle for 15d. Despite similar food intake, Dex‐treated animals lost body weight, which was not rescued by 1709, and evaluation of body composition revealed no statistically significant difference between Dex and Dex + 1709 groups, although Dex + 1709 groups displayed a modest resistance to Dex‐induced lean mass loss (Figure S5A–C). Tibialis anterior (TA) and soleus muscle weights also were not significantly different between the Dex vs. Dex + 1709 groups, and here, a modest resistance in loss of TA, but not soleus muscle mass in the Dex + 1709 treated animals was observed (Figure S5D). However, histological analysis of GN muscle revealed that Dex markedly reduced myofibre cross‐sectional area and Feret's diameter, while 1709 preserved them (Figure 5B).

Muscle damage is typically accompanied by induction of atrogenes; MuRF1 and Atrogin‐1 [8, 11, 13]. Following injury/damage, skeletal muscle undergoes repair, wherein self‐renewing Pax7+/MyoD‐ myosatellite cells are recruited toward myogenic lineage characterized by a Pax7+/MyoD+ state, and these committed cells then enter differentiation and the completion of which is evidenced by the loss of Pax7 and induction of myogenin expression [40]. Dex increased expression of Atrogin‐1, MuRF1, Pax7 and PCNA (proliferating cell nuclear antigen), with reduced MyoD and myogenin (Figure 5C) indicating ongoing damage and delayed regeneration. In contrast, GN muscles from Dex + 1709 animals showed normalized Atrogin‐1 and MuRF1, and elevated MyoD and myogenin expressions, comparable to vehicle controls (Figure 5C). These results were validated by QRT‐PCR where similar patterns were observed (Figure S5E). These results indicated that either 1709 protected against Dex‐induced damage, or the regeneration in these animals was faster, or both. To rule out the probability that 1709 is a GR antagonist the effect of 1709/gAd/AdipoRon was evaluated in a Dex‐mediated GR‐GRE‐luc reporter assay and neither of the AdipoR agonists meaningfully impacted Dex‐mediated GR activity (Figure S6).

Evaluation of the adiponectin signalling components revealed that Dex suppressed AMPK/AKT/p38 phosphorylations and reduced PGC‐1α expression in GN muscle, all of which were restored by 1709. PPARα remained suppressed, consistent with AdipoR1 selectivity of 1709 (Figure 5C). Dex reduced local muscle adiponectin while increasing circulating levels, and 1709 restored muscle adiponectin without altering plasma adiponectin (Figure 5C,D). AdipoR1 and 2 expressions were unaltered across all groups (Figure 5C).

Fibre‐type marker analysis revealed: consistent with Figure 3A, GN muscle from the Dex group displayed significantly lower MyHC‐I and increased glycolytic MyHC‐IIB expression, and 1709 reversed them (Figure 5C). Fast oxidative MyHC‐IIA also showed a similar pattern to MyHC‐I (albeit not statistically significant) (Figure 5C).

Functionally, Dex‐treated animals exhibited reduced grip strength and wire‐hanging performance, which were significantly improved by 1709, exceeding vehicle controls. Rotarod performance, however, was not restored by 1709 (Figure 5E). Together, these data demonstrate that 1709 protects against Dex‐induced skeletal muscle atrophy by restoring Adiponectin signalling, fibre‐type balance and muscle function.

### 3.6 CDRI‐1709S Protected Against Denervation‐Induced Skeletal Muscle Atrophy

Since Dex affects multiple organs, in addition to its direct effect on muscles, Dex‐induced atrophy may also occur from cumulative dysfunction of other organs. Consequently, improvement in any of these systems could contribute to the apparent protective effects of 1709. To thus determine whether 1709 exerts a direct protective effect on skeletal muscle, we evaluated its efficacy in a local sciatic nerve denervation‐induced model of muscle atrophy.

**FIGURE 6:**
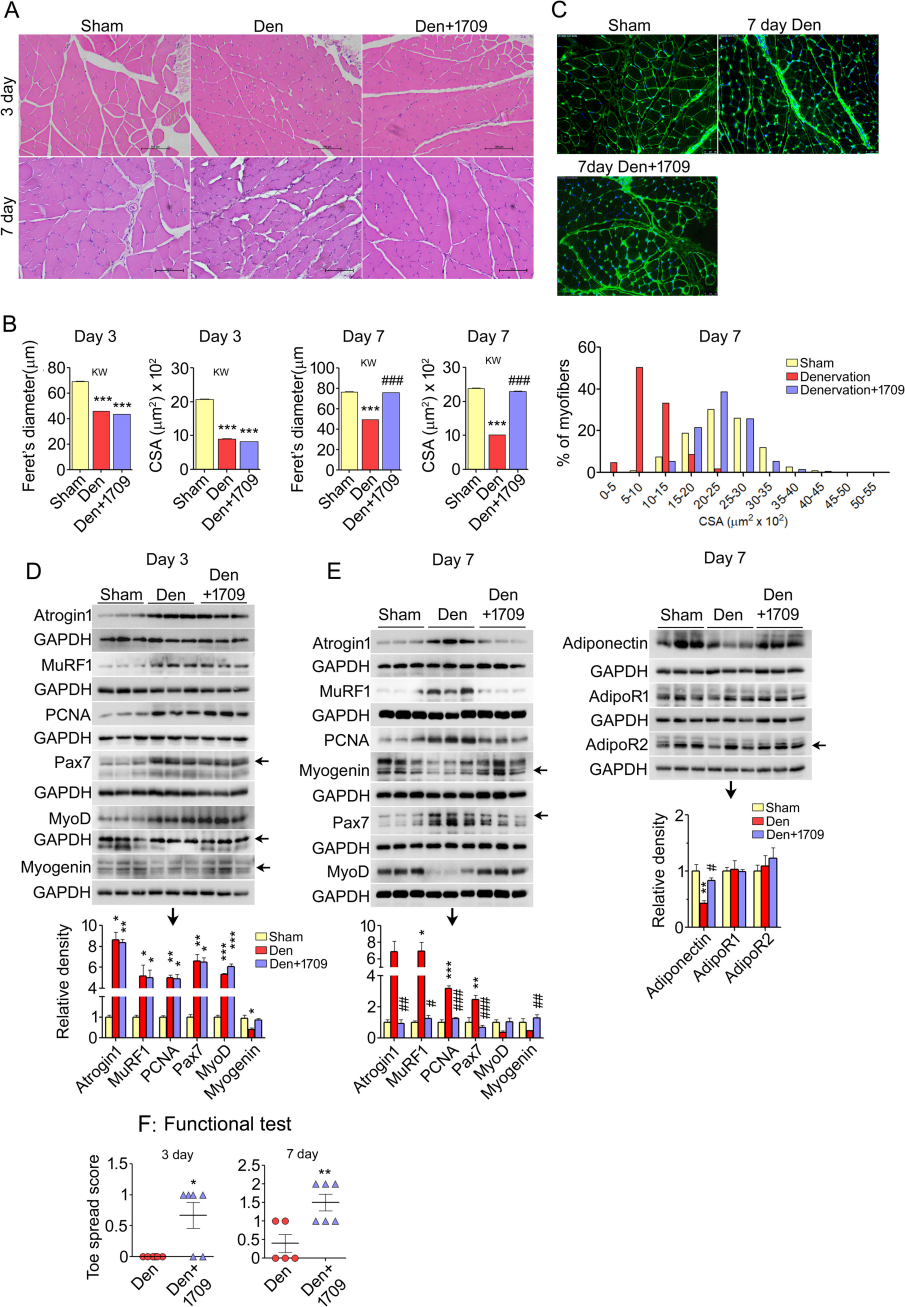
CDRI‐1709S protected against 7 day denervation‐induced skeletal muscle atrophy in SD rats. As described in Materials and methods, male SD rats were randomized into two groups: vehicle‐treated and 1709‐treated (*n* = 6/group), following sciatic nerve‐denervation. (A) Compound 1709 restored denervation‐induced alterations in myofibre architecture. A. Representative images of H&E‐stained GN muscle transverse sections of sham or Den+1709 group animals at day 3 day or 7 day following denervation were captured using a Nikon Eclipse E200 microscope (magnification 200×; Scale bar: 100 μm). (B) Graphs representing quantification of CSA and Min. Feret's diameter, and % distribution of myofibres based on CSA, were plotted following morphometry of H&E‐stained GN muscle sections. (C) Representative fluorescence images from 7 day, showing WGA‐stained GN muscle sections. (Green fluorescence: WGA bound to sarcolemma, blue fluorescence: DAPI stained nuclei). Images were captured using a Leica DMI6000B fluorescence microscope (magnification 200×; scale bar: 50 μm). (D, E) Compound 1709 induced the recovery from Den‐induced damage and adiponectin expression in muscle. Expression of proteins (in GN muscle) as indicated were analysed from 3 day (D) or 7 day (E) denervation experiments by immunoblotting. Each lane represents protein sample from a single animal. Immunoblots represent protein expression of three animals/group (immunoblots of samples from other animals are provided in Supporting Information S1: full blots). Graphs represent quantification using protein expression of all 6 animals/group. (F) Compound 1709 improved Denervation‐induced functional impairment. Toe‐spreading ability was used to assess functional improvement in denervated legs at 3 day (*n* = 6/group) and 7 day (*n* = 5 for Sham and Den, *n* = 6 for Den+1709). Quantification performed using ImageJ. Graphs represent mean ± SEM */# *p* < 0.05, **/## *p* < 0.01, ***/### *p* < 0.0001. Statistical analyses were performed by 1‐Way ANOVA followed by Bonferroni's post‐test (D: MyoD, Myogenin. E: MyoD) or Kruskal–Wallis test followed by Dunn's post‐test (B: Day 3 and Day 7 CSA and Feret's diameter. D: MuRF1, Atrogin1, PCNA, Pax7. E: MuRF1, Atrogin1, Myogenin, adiponectin, AdipoR1, AdpoR2) and unpaired, two‐tailed t‐test (F) *Control vs. other groups, #Den vs. Den+1709. WGA: Wheat germ agglutinin, Den: Denervation.

Sciatic nerve denervation was performed on the left hindlimb of male SD rats, while the contralateral limb served as a sham control. Following denervation, the rats were either fed vehicle or 1709. Left and right limbs from vehicle‐treated rats served as Den and control groups respectively, while the denervated left limbs from 1709‐treated animals were labelled as Den+1709. Morphometry of H&E‐stained GN sections (Figure 6A) showed a marked reduction in Feret's diameter and cross‐sectional area (CSA) at both day 3 and day 7 and a pronounced increase in small myofibres in the day 7 Den group, compared to sham (Figure 6B). Restoration of these parameters by 1709 was evident only at day 7 (Figure 6A,B). WGA staining further reiterated these observations (Figure 6C).

At day 3, GN muscles from denervated limbs of both vehicle and 1709 groups showed elevated Atrogin‐1, MuRF1, PCNA, Pax7 and MyoD expression relative to sham (Figure 6D). Myogenin, however, was reduced in the Den group but preserved in Den+1709 muscles (Figure 6D). By day 7, Atrogin‐1, MuRF1, PCNA and Pax7 remained elevated in Den muscles but were restored to sham levels in Den+1709 muscles (Figure 6E). Similarly, MyoD and myogenin expression remained suppressed in the Den group but was comparable to sham in Den+1709 muscles at day 7 (Figure 6E). Similar to the Dex model, local adiponectin expression was significantly reduced in Den muscles at day 7 but preserved in Den+1709 muscles, while AdipoR1 and AdipoR2 levels remained unchanged (Figure 6E). No differences in adiponectin expression were observed at day 3 (data not shown).

Muscle function was assessed using a previously described toe‐spread test [17]. At day 3, partial toe spreading was observed in 4/6 animals in the Den+1709 group, compared to none in the Den group (Figure 6F, Figure S7). By day 7, partial toe spreading was observed in 2/5 Den animals, while in the Den+1709 group, three animals showed partial and three animals showed complete toe spreading (Figure 6F).

To assess if 1709‐treatment could elicit any response in unchallenged muscles, we compared the molecular parameters corresponding to Figure 6D in the GN muscles from the sham‐operated limbs of the 7‐day Vehicle and 1709‐treated animals. As shown in Figure S8, no difference in the expression of MuRF‐1, Atrogin‐1, MyoD, Myogenin, adiponectin, AdipoR1/2, MyHC‐1 and MyHC‐IIB was observed between the groups, and although MyHC‐1IA showed a modest trend of increase in 1709‐treated muscles, it was statistically not significant. Since adiponectin is the most abundant circulating biomolecule [1] and healthy muscles express abundant AdipoRs, it appears that added adiponectin/mimetics may not cause any change over and above the normal adiponectin response.

Collectively, these data indicate that while 1709 provided modest protection against denervation‐induced atrophy at day 3, it significantly accelerated muscle regeneration by day 7, resulting in pronounced structural, molecular and functional recovery.

## 4 Discussion

Here we report the discovery and comprehensive characterization of the synthetic pterocarpan 1709 as the first selective AdipoR1 agonist and demonstrate its myogenic and anti‐atrophy efficacy across multiple in vitro and in vivo models of skeletal muscle atrophy.

Our group has previously established that AdipoR agonists possess osteoanabolic and osteoprotective properties [24, 35] [S21, S27]. Based on this premise, we screened a library of compounds originally identified as osteogenic for their AdipoR‐activating potential, which led to the identification of 1709, a 3‐hydroxy pterocarpan previously reported and patented for its osteogenic activity [34], as a highly potent AdipoR1‐selective agonist with an EC_50_ of 414.7 pM.

At pharmacologically relevant concentrations, 1709 activated hallmark adiponectin‐associated signalling events, including phosphorylation of AMPK, AKT and p38‐MAPK, in AdipoR1‐overexpressing HEK293T and AdipoR‐rich C2C12 myotubes. These effects were abolished by AdipoR1 but not AdipoR2 knockdown, confirming receptor selectivity. Consistent with this specificity, 1709 failed to induce PPARα, a canonical AdipoR2 target [36], whereas the dual agonist gAd induced PPARα expression. Intriguingly, gAd‐mediated PPARα induction required both AdipoR1 and AdipoR2, suggesting functional cooperation between the receptor isoforms. Similar observations were reported previously for the selective AdipoR2 agonist isovitexin, where AdipoR1‐knockdown compromised its downstream signalling [21]. These findings highlight a complex and interdependent AdipoR signalling network that warrants further investigation.

Given the well‐established role of adiponectin and AdipoR1 in skeletal muscle metabolism, regeneration and protection from atrophy [4, 23, 26, 27, 33] [S4, S14, S15, S28], we evaluated the myogenic and anti‐atrophy potential of 1709. As a single agent, 1709 induced C2C12 myoblast differentiation, evidenced by myotube formation and increased expression of MyoD, myogenin and MyHC. In differentiated myotubes, 1709 protected against atrophy induced by corticosteroids, inflammatory cytokines and nutrient deprivation, preserving myotube morphology and suppressing the induction of Atrogin‐1 and MuRF1.

Mechanistically, inhibition studies revealed that AMPK, AKT and p38‐MAPK were all required for the myogenic effects of 1709, whereas p38‐MAPK was dispensable for its anti‐atrophy actions, indicating pathway divergence between differentiation and atrophy protection. Detailed analysis of Dex‐treated myotubes showed a shift toward a fast glycolytic phenotype, with increased MyHC‐IIB and reduced MyHC‐I and MyHC‐IIA expression. Notably, despite this glycolytic shift, both glucose utilization and oxidative capacity were impaired, suggesting that Dex may drive conversion of oxidative fibres into a metabolically compromised glycolytic state. This phenomenon, together with known corticosteroid‐induced insulin resistance and lipolysis [39], may contribute to muscle vulnerability in chronic glucocorticoid exposure. Importantly, 1709 reversed Dex‐induced fibre‐type switching and restored both glycolytic flux and mitochondrial OCR. Similar findings were observed in vivo in the Dex‐induced rat model, where 1709 preserved myofibre size, normalized fibre‐type distribution, restored adiponectin signalling and improved muscle strength. Despite failing to rescue Dex‐mediated body weight loss and altered body composition, 1709 treatment significantly improved grip strength and wire‐hanging performance, surpassing even vehicle‐treated animals. Although we did not see any statistically significant improvement in muscle and/or lean mass, the Dex + 1709 group showed modestly higher resistance to the Dex‐induced loss in TA weight and lean mass. Given that we routinely experience mortality issues upon longer exposure to Dex in SD rats, we aim to perform future rehabilitation experiments in Dex vs. Dex + 1709‐treated animals following withdrawal of Dex treatment after 15 days to assess if 1709 could accelerate restoration of muscle and lean mass. The lack of improvement in rotarod performance suggests that while AdipoR1 activation enhances muscle strength, it may not fully correct Dex‐induced impairments in motor coordination or proprioception, and since both AdipoR1 and AdipoR2 are expressed in the brain [6], dissection of the roles of each isoform in brain merits further study.

At the molecular level, Dex reduced local muscle adiponectin without altering AdipoR expression, suppressed AMPK/AKT/p38 signalling and decreased PGC‐1α and PPARα expression. 1709 restored all these parameters except PPARα, consistent with its AdipoR1 selectivity. These suggest that fibre‐type specification and oxidative capacity in skeletal muscle can be regulated independently of PPARα, raising the possibility that AdipoR1 engages alternative lipid‐utilization pathways.

To exclude systemic confounders inherent to Dex treatment, we evaluated 1709 in a sciatic nerve denervation model. Early after denervation (day 3), both vehicle‐ and 1709‐treated muscles showed architectural deterioration and induction of atrogenes; however, 1709 preserved myogenin expression and partial functional ability. By day 7, 1709 markedly accelerated regeneration, restoring muscle architecture, normalizing satellite cell activation markers, reducing atrogene expression and significantly improving function. As observed in the Dex model, denervation also reduced local muscle adiponectin, which was restored by 1709 at later stages, indicating that local adiponectin depletion may be a common feature of muscle injury and atrophy. The improvement following 1709 treatment does not appear due to reinnervation as the excision and removal of a 5‐mm section of the sciatic nerve followed by ligation of the ends does not allow reinnervation, at least up to 21 days, which was established in our lab while optimizing this model [17]. Denervation is followed by a huge calcium flux causing rapid induction of atrogenes, substantial rise in inflammation and cellular ROS, which is associated with edema, muscle stiffness, fibrosis and follow‐up further secondary atrophy [S30]. Since AdipoR activation reduces inflammation, ROS and fibrosis [33] the improvement in toe‐spread might be a result of reduced muscle degradation, fibrosis, edema, stiffness and overall local mechanical improvement rather than reinnervation, and we have reported similar findings earlier for liraglutide [17].

## 5 Conclusion

We report CDRI‐1709S as the first small‐molecule selective AdipoR1 agonist that induces myogenesis and protects against skeletal muscle atrophy at pharmacologically relevant concentrations via activation of hallmark adiponectin signalling, enhancing oxidative capacity and accelerated muscle regeneration. These findings strengthen the therapeutic rationale for targeting adiponectin signalling in muscle wasting disorders and provide a foundation for developing next‐generation translation‐worthy AdipoR1‐selective agents. This study however, needs to be extended to long‐term treatment in unchallenged preclinical models to investigate if such small‐molecule AdipoR agonists can induce molecular effects over and above the already abundant circulating adiponectin level, in muscle and other organs, particularly heart, where beneficial effects of adiponectin signalling are well reported.

## Funding

This work was supported by the Council of Scientific and Industrial Research, India (MLP2028) and Blockchain for Impact‐BIOME (GAP0485).

## Ethics Statement

All in vivo experimental procedures were approved by the Institutional Animal Ethics Committee (IAEC) of CSIR‐Central Drug Research Institute; Approval No. (IAEC/2022/44).

## Conflicts of Interest

The authors declare no conflicts of interest.

## Supporting information


**Table S1:** List of antibodies.
**Table: S2.** QRTPCR primer sequences.
**Figure S1:** CDRI‐1709S treatment in myotubes activates adiponectin‐associated rapid signalling events. C2C12 myotubes were treated with V, 1709 (100 nM/1 μM) or gAd (1 μg/mL) for 10 min and protein expression was analysed by immunoblotting (*n* = 3). Graph represents mean ± SEM *V vs. treatment groups. *p < 0.05, **p < 0.01, ***p < 0.0001. Statistical analysis was performed using 1‐way ANOVA followed by Bonferroni's post‐test. gAd; Globular adiponectin.
**Figure S2:** CDRI‐1709S treatment in C2C12 myotubes induces and activates factors involved in adiponectin signalling pathways, and AdipoR1/R2 knockdown compromises it. C2C12 myotubes were infected with lentivirus (Set‐2) containing shscr/AdipoR1/AdipoR2 shRNAs. Forty‐eight hours after infection, cells were treated as depicted for 10 min (S 2A) or 24 h (S 2B). Phospho‐proteins were normalized with their respective total proteins (on the same blot), for other proteins, β‐actin (on the same blot) was used as normalizing control. Graphs represent mean ± SEM (*n* = 3). *p < 0.05, **p < 0.01, ***p < 0.0001. Statistical analyses were performed by 2‐way ANOVA (S2A‐B) followed by Bonferroni's post‐test.
**Figure S3:** CDRI‐1709S reverses Dex induction of atrogenes and restores Dex‐mediated suppression of myogenin mRNA expression in C2C12 myotubes. Graph represents mean ± SEM. *V vs. treatment groups, #Dex vs. treatment groups, */#*p* < 0.05, **/##*p* < 0.01, ***/##*p* < 0.0001. Statistical analysis was performed using Kruskal–Wallis test followed by Dunn's post‐test. Aron: Adiporon. This experiment also included another treatment group corresponding to Medicarpin; a compound we identified as a dual AdipoR1/R2 agonist, which is being reported separately thus we declare that both this and the Medicarpin studies share the same V, Dex, Dex+gAd and Dex+Aron datasets.
**Figure S4:** CDRI‐1709S ameliorates (LPS + TNFα; LT), and nutrientdeficiency (PBS)–induced myotube shrinkage. Phase contrast microscopic images of LTinduced atrophy model (A), PBS without Ca2 + (B) and PBS with Ca2 + (C). Images in A were captured using a Nikon ECLIPSE Ts2 microscope (magnification × 100; scale bar, 10 μm, 18 fields/treatment group). Images in B and C were captured using EVOS FL Auto Imaging System (Life Technologies) (magnification × 100; scale bar 400 μm, 18 fields/treatment group). Morphometric analyses of microscopic images (A–C) are shown in Figure 3. PBS; phosphate buffer saline.
**Figure S5:** CDRI‐1709S does not affect GR activation by Dex in a GRELuciferase activity assay. GRE‐luc reporter assay was performed in HEK‐293 T cells as described in materials and methods of the main manuscript. Data represent mean ± SEM. *p < 0.05, **p < 0.01, ***p < 0.0001. *Vehicle vs. test compounds in GR‐transfected groups. Statistical analysis was performed by 2‐way ANOVA followed by Bonferroni's post‐test.
**Figure S6:** Evaluation of the effect of Dex and CDRI‐1709S on feed‐intake, body composition, muscle weight and muscle mRNA expressions. Feed intake (A) body weight (B) of rats from indicated groups (*n* = 6/group). (C) Lean mass and Fat mass (in grams) of rats from indicated groups (*n* = 6/group) measured using an EchoMRI‐500 body composition analyser (EchoMRI Corporation Pvt. Ltd. Singapore). D. Absolute and relative muscle weights of TA and Soleus muscles from left hindlimbs (*n* = 6/group). E. Transcript levels of Atrogin1, MuRF1 and Myogenin in GN muscle of rats from indicated treatment groups (*n* = 3 in triplicates). All graphs represent mean ± SEM. *V vs. treatment groups, #Dex vs. treatment groups. */#*p* < 0.05, **/##*p* < 0.01, ***/##*p* < 0.0001. Statistical analysis performed using 1‐way ANOVA followed by Bonferroni's post‐test for (C, D, E). For Atrogin1, MuRF1 and Myogenin in E. Kruskal–Wallis test was performed followed by Dunn's post‐test.
**Figure S7:** Paw prints taken on 3rd day and 7th day, post‐denervation. Paw print of each rat is labelled along with their respective scores. Left hind limb in each case was denervated and the right limb served as sham and was considered as benchmark (score: 2, indicating complete opening of paws). Corresponding graphs are in main Figure 6F.
**Figure S8:** CDRI‐1709s treatment does not significantly alter expressions of the proteins examined in GN muscles from sham‐operated limbs. A. Immunoblot panels represent expression of indicated proteins in GN muscles from sham‐operated limbs of vehicle (*n* = 5) or 1709 (*n* = 6)‐treated rats corresponding to the denervation experiment (main Figure 6). Graph represents densitometric analysis (mean ± SEM). MyHC‐I, MyHC‐IIA and MyHC‐IIB were normalized with α‐actinin, and the rest were normalized with GAPDH. Statistical analysis was performed by unpaired, two‐tailed Student's *t*‐test, except for AdipoR2, MyHC‐I, MyHCIIA and MyHC‐IIB, where Mann–Whitney test was used. F test was used to assess intra‐group variances.

**Data S1:** Supporting Information.

## Data Availability

The data that support the findings of this study are available from the corresponding author upon reasonable request.
