# Supplementary material for: Selective Small‐Molecule AdipoR1 Agonist 3‐Hydroxy Pterocarpan Salt (CDRI‐1709S) Ameliorates Skeletal Muscle Atrophy
Source: J Cachexia Sarcopenia Muscle. 2026 Jul 21;17(4):e70328. doi: 10.1002/jcsm.70328 (PMC13387834; doi:10.1002/jcsm.70328)
Supplement: Supplementary file 1 — Table S1: List of antibodies. Table: S2. QRTPCR primer sequences. Figure S1: CDRI‐1709S treatment in myotubes activates adiponectin‐associated rapid signalling events. C2C12 myotubes were treated with V, 1709 (100 nM/1 μM) or gAd (1 μg/mL) for 10 min and protein expression was analysed by immunoblotting (n = 3). Graph represents mean ± SEM *V vs. treatment groups. *p < 0.05, **p < 0.01, ***p < 0.0001. Statistical analysis was performed using 1‐way ANOVA followed by Bonferroni's post‐test. gAd; Globular adiponectin. Figure S2: CDRI‐1709S treatment in C2C12 myotubes induces and activates factors involved in adiponectin signalling pathways, and AdipoR1/R2 knockdown compromises it. C2C12 myotubes were infected with lentivirus (Set‐2) containing shscr/AdipoR1/AdipoR2 shRNAs. Forty‐eight hours after infection, cells were treated as depicted for 10 min (S 2A) or 24 h (S 2B). Phospho‐proteins were normalized with their respective total proteins (on the same blot), for other proteins, β‐actin (on the same blot) was used as normalizing control. Graphs represent mean ± SEM (n = 3). *p < 0.05, **p < 0.01, ***p < 0.0001. Statistical analyses were performed by 2‐way ANOVA (S2A‐B) followed by Bonferroni's post‐test. Figure S3: CDRI‐1709S reverses Dex induction of atrogenes and restores Dex‐mediated suppression of myogenin mRNA expression in C2C12 myotubes. Graph represents mean ± SEM. *V vs. treatment groups, #Dex vs. treatment groups, */#p < 0.05, **/##p < 0.01, ***/##p < 0.0001. Statistical analysis was performed using Kruskal–Wallis test followed by Dunn's post‐test. Aron: Adiporon. This experiment also included another treatment group corresponding to Medicarpin; a compound we identified as a dual AdipoR1/R2 agonist, which is being reported separately thus we declare that both this and the Medicarpin studies share the same V, Dex, Dex+gAd and Dex+Aron datasets. Figure S4: CDRI‐1709S ameliorates (LPS + TNFα; LT), and nutrientdeficiency (PBS)–induced myotube shrinkage. Phase con [file JCSM-17-e70328-s001.pdf]

Table S1: List of antibodies

Antibody	Catalogue no.	Manufacturer	Dilution
Adiponectin (19F1)	MA1-054	Thermo Fisher Scientific	1:1000
AdipoR1	DF6430	Affinity Biosciences	1:1000
AdipoR2	DF12811	Affinity Biosciences	1:1000
Phospho-AMPKα (Thr172) (40H9)	#2535	Cell Signaling Technology	1:1000
AMPKα (D5A2)	#5831	Cell Signaling Technology	1:1000
Phospho-p38 MAPK (Thr180/Tyr182) (D3F9)	#4511	Cell Signaling Technology	1:1000
p38 MAPK (D13E1)	#8690	Cell Signaling Technology	1:1000
Phospho-Akt (Ser473) (193H12)	#4058	Cell Signaling Technology	1:1000
Akt (pan) (C67E7)	#4691	Cell Signaling Technology	1:1000
Pax7 (EPR3353)	ab92317	Abcam	1:1,000
PCNA (PC10)	#2586	Cell Signaling Technology	1:1000
PGC-1α	ab54481	Abcam	1:1000
PPARα (3B6/PPAR)	MA1-822	Thermo Fisher Scientific	1:1000
Glut4 (1F8)	#2213	Cell Signaling Technology	1:1000
UCP3	ab3477	Abcam	1:1000
CD36	sc-7309	Santa Cruz	1:1000
MyoD	MA1-41017	Thermo Fisher Scientific	1:1000
Myogenin	DF8273	Affinity Biosciences	1:1000
Myogenin	ab124800	Abcam	1:1000
MURF1	ab172479	Abcam	1:1000
Fbx32	ab168372	Abcam	1:1000
MyHC (MF20)	DSHB, #MF20	DSHB	1:200
Myosin heavy chain Type IIA	DSHB, #SC-71	DSHB	0.3μg/mL
Myosin heavy chain Type IIB	DSHB, #BF-F3	DSHB	0.3μg/mL
Myosin heavy chain Type I	DSHB, #BA-D5	DSHB	0.3μg/mL
GAPDH (14C10)	#3683	Cell Signaling Technology	1:1000
Rat Adiponectin Rapid ELISA Kit	EELR028	Thermo Fisher Scientific	1:2
β-Actin	A3854	Sigma-Aldrich	1:35,000
Anti-Rabbit IgG secondary	A9169	Sigma-Aldrich	1:10,000
Anti-Mouse IgG secondary	A9044	Sigma-Aldrich	1:10,000
α-actinin	6487	Cell Signaling Technology	1:1000

Table: S2. QRTPCR primer sequences

Rat	Forward Primer (5'---3')	Tm (°C)	Reverse Primer (5'---3')	Tm (°C)	Accession no.
Atrogin1	GAAGACCGGCTACTGTGGAA	59.4	ATCAATCGCTTGCGGATCT	54.5	NM_133521.2
MuRF1	AGGACTCCTGCCGAGTGAC	61.0	TTGTGGCTCAGTTCCTCCTT	57.3	AY059627.1
Myogenin	CTACAGGCCTTGCTCAGCTC	57.9	TGGGAGTTGCATTCACTGG	55.3	NM_017115.3
GAPDH	TGGGAAGCTGGTCATCAAC	56.7	GCATCACCCCATTTGATGTT	55.3	NM_017008.4
**Mouse**	**Forward Primer (5'---3')**	**Tm (°C)**	**Reverse Primer (5'---3')**	**Tm (°C)**	**Accession no.**
MuRF1	CCTGCAGAGTGACCAAGGA	58.94	GGCGTAGAGGGTGTCAAACT	59.68	NM_001369245.1
Atrogin1	GATCAAACGCTTGCGAATCT	57.19	AGTGAGGACCGGCTACTGTG	61.25	NM_026346.3
Myogenin	CTACAGGCCTTGCTCAGCTC	60.46	TGGGAGTTGCATTCACTGG	57.66	NM_031189.2
β-actin	CCTCACCCTCCCAAAAGC	57.58	GTGGACTCAGGGCATGGA	58.60	NM_007393.5

Supplementary Figure S1

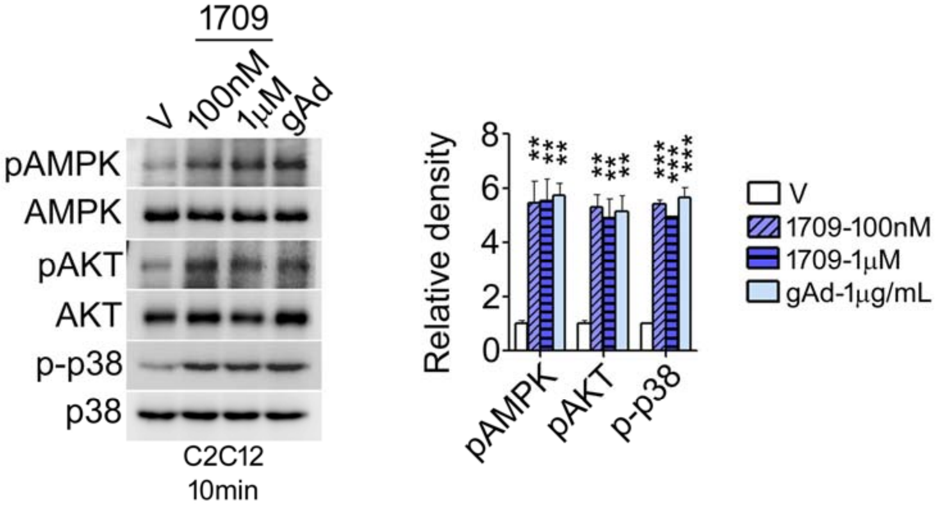

Supplementary Figure S1. CDRI-1709S treatment in myotubes activates adiponectin-associated rapid signaling events. C2C12 myotubes were treated with V, 1709 (100nM/1μM) or gAd (1μg/mL) for 10 min, and protein expression was analyzed by immunoblotting (n=3). Graph represents mean ± SEM. *V vs. treatment groups. *p <0.05, **p <0.01, ***p <0.0001. Statistical analysis was performed using 1-way ANOVA followed by Bonferroni's post-test. gAd; Globular Adiponectin.

Supplementary Figure S2

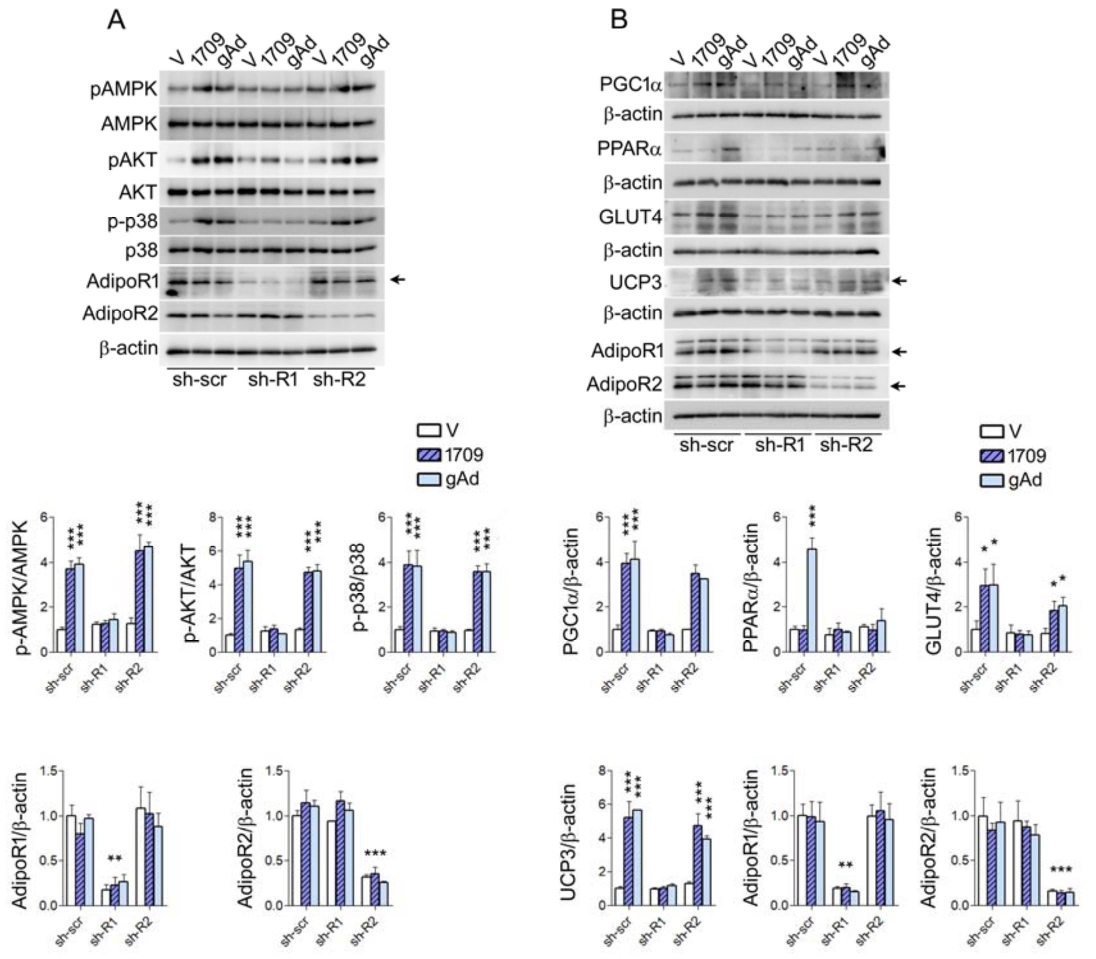

Supplementary Figure S2. **CDRI-1709S treatment in C2C12 myotubes induces and activates factors involved in Adiponectin signaling pathways, and AdipoR1/R2 knockdown compromises it**. C2C12 myotubes were infected with lentivirus (Set-2) containing sh-scr/AdipoR1/AdipoR2 shRNAs. 48 h after infection, cells were treated as depicted for 10 min (S 2A) or 24 h (S 2B). Phospho-proteins were normalized with their respective total proteins (on the same blot), for other proteins, β-actin (on the same blot) was used as normalizing control. Graphs represent mean ± SEM (n = 3). *p <0.05, **p <0.01, ***p <0.0001. Statistical analyses were performed by 2-way ANOVA (S2A-B) followed by Bonferroni's post-test.

Supplementary Figure S3

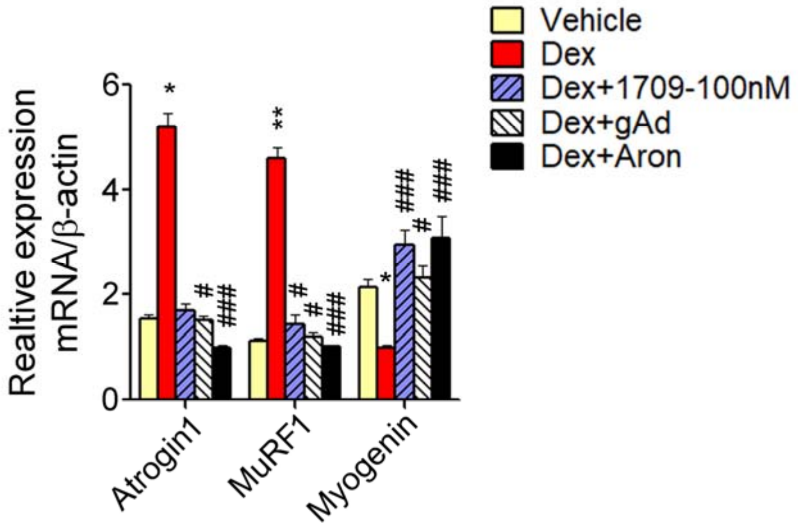

Supplementary Figure S3. CDRI-1709S reverses Dex induction of atrogenes and restores Dex-mediated suppression of myogenin mRNA expression in C2C12 myotubes.
Graph represents mean ± SEM. *V vs. treatment groups, #Dex vs treatment groups, */#p <0.05, **/##p <0.01, ***/##p <0.0001. Statistical analysis was performed using Kruskal-Wallis test followed by Dunn's post-test. Aron: Adiporon. *This experiment also included another treatment group corresponding to Medicarpin; a compound we identified as a dual AdipoR1/R2 agonist, which is being reported separately thus we declare that both this and the Medicarpin studies share the same V, Dex, Dex+gAd, and Dex+Aron datasets.*

Supplementary Figure S4

A

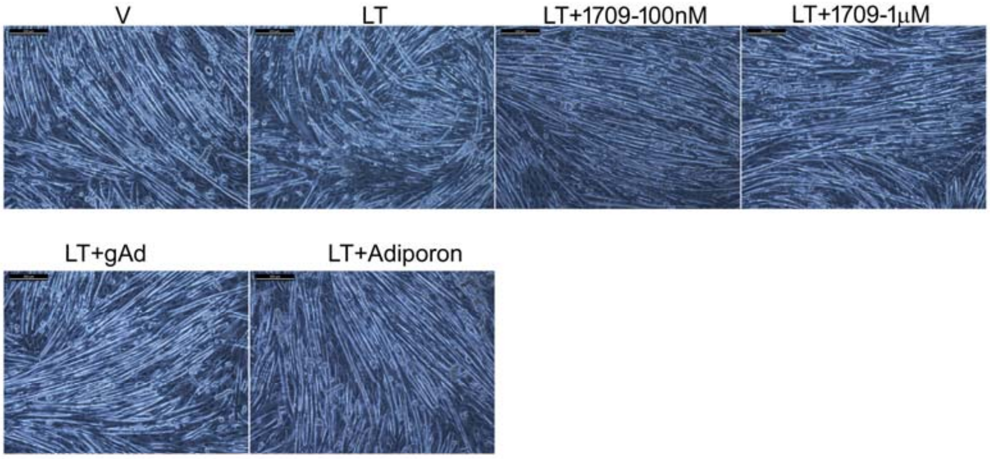

B

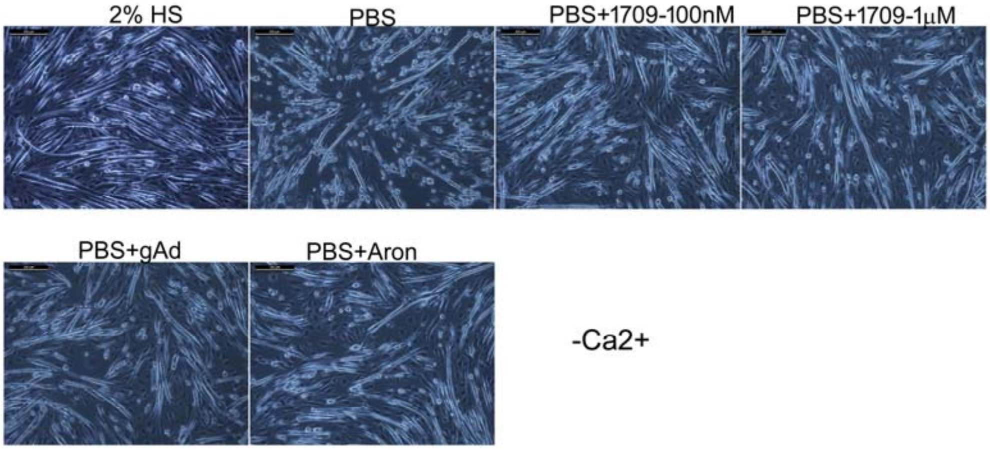

C

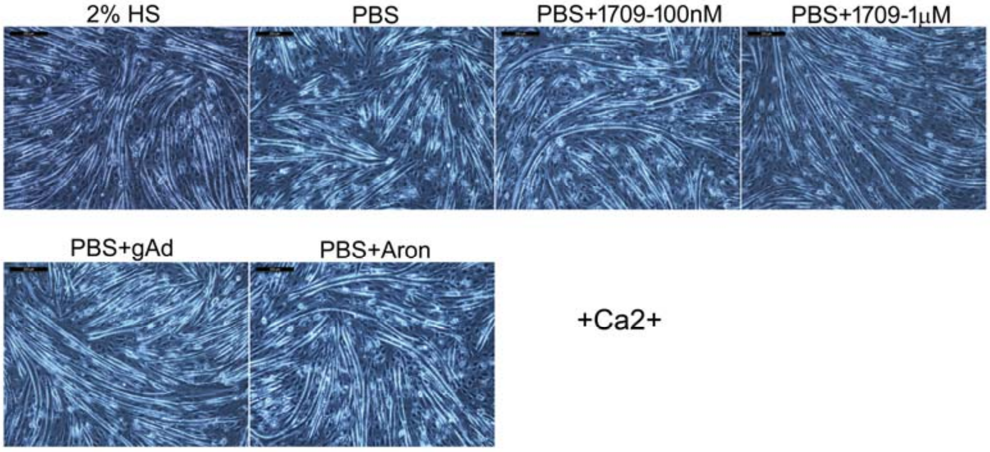

Supplementary Figure S4. CDRI-1709S ameliorates (LPS + TNFα; LT), and nutrient-deficiency (PBS)–induced myotube shrinkage. Phase contrast microscopic images of LT-induced atrophy model (**A**), PBS without Ca^{2+} (**B**) and PBS with Ca^{2+} (**C**). Images in **A** were captured using a Nikon ECLIPSE Ts2 microscope (magnification, x 100; scale bar, 10μm, 18 fields/treatment group). Images in **B** and **C** were captured using EVOS FL Auto Imaging System (Life Technologies) (magnification x 100; scale bar 400μm, 18 fields/treatment group). Morphometric analyses of microscopic images (**A-C**) are shown in **Figure 3**. PBS; phosphate buffer saline.

Supplementary Figure S5

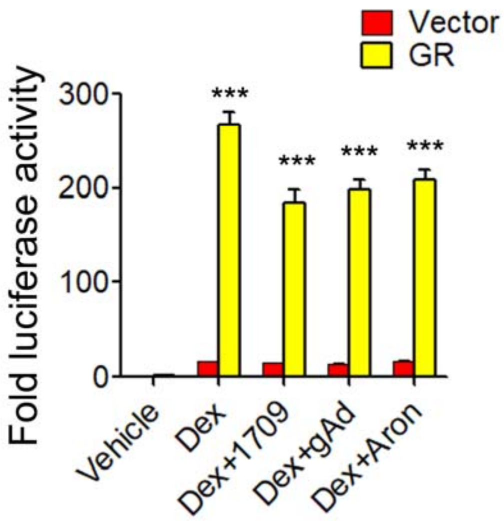

Supplementary Figure S5. CDRI-1709S does not affect GR activation by Dex in a GRE-Luciferase activity assay. GRE-luc reporter assay was performed in HEK-293T cells as described in materials and methods of the main manuscript. Data represent mean ± SEM. *p <0.05, **p <0.01, ***p <0.0001. *Vehicle vs test compounds in GR-transfected groups. Statistical analysis was performed by 2-way ANOVA followed by Bonferroni's post-test.

Supplementary Figure S6

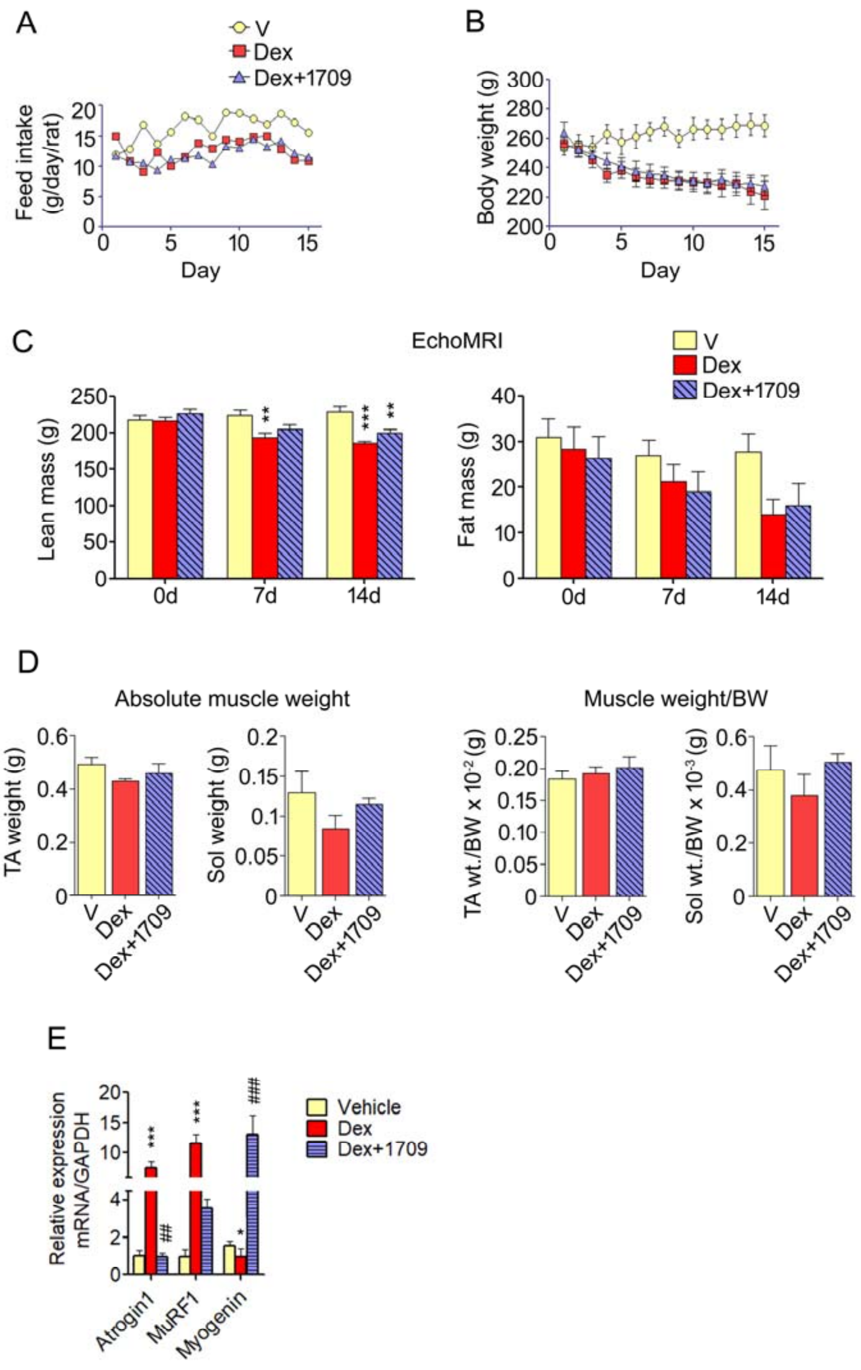

Supplementary figure S6. Evaluation of the effect of Dex and CDRI-1709S on feed-intake, body composition, muscle weight, and muscle mRNA expressions. Feed intake (**A**) body weight (**B**) of rats from indicated groups (n=6/group). **C.** Lean mass and Fat mass (in grams) of rats from indicated groups (n=6/group) measured using an EchoMRI-500 body composition analyzer (EchoMRI Corporation Pvt. Ltd. Singapore). **D. Absolute and relative muscle weights**

of TA and Soleus muscles from left hindlimbs (n=6/group). **E.** Transcript levels of Atrogin1, MuRF1 and Myogenin in GN muscle of rats from indicated treatment groups (n=3 in triplicates). All graphs represent mean ± SEM. *V vs treatment groups, #Dex vs treatment groups. */#p < 0.05, **/##p < 0.01, ***/##p < 0.0001. Statistical analysis performed using 1-way ANOVA followed by Bonferroni's post-test for **C**, **D**, **E**. For Atrogin1, MuRF1 and Myogenin in **E**. Kruskal-Wallis test was performed followed by Dunn's post-test.

Supplementary figure S7

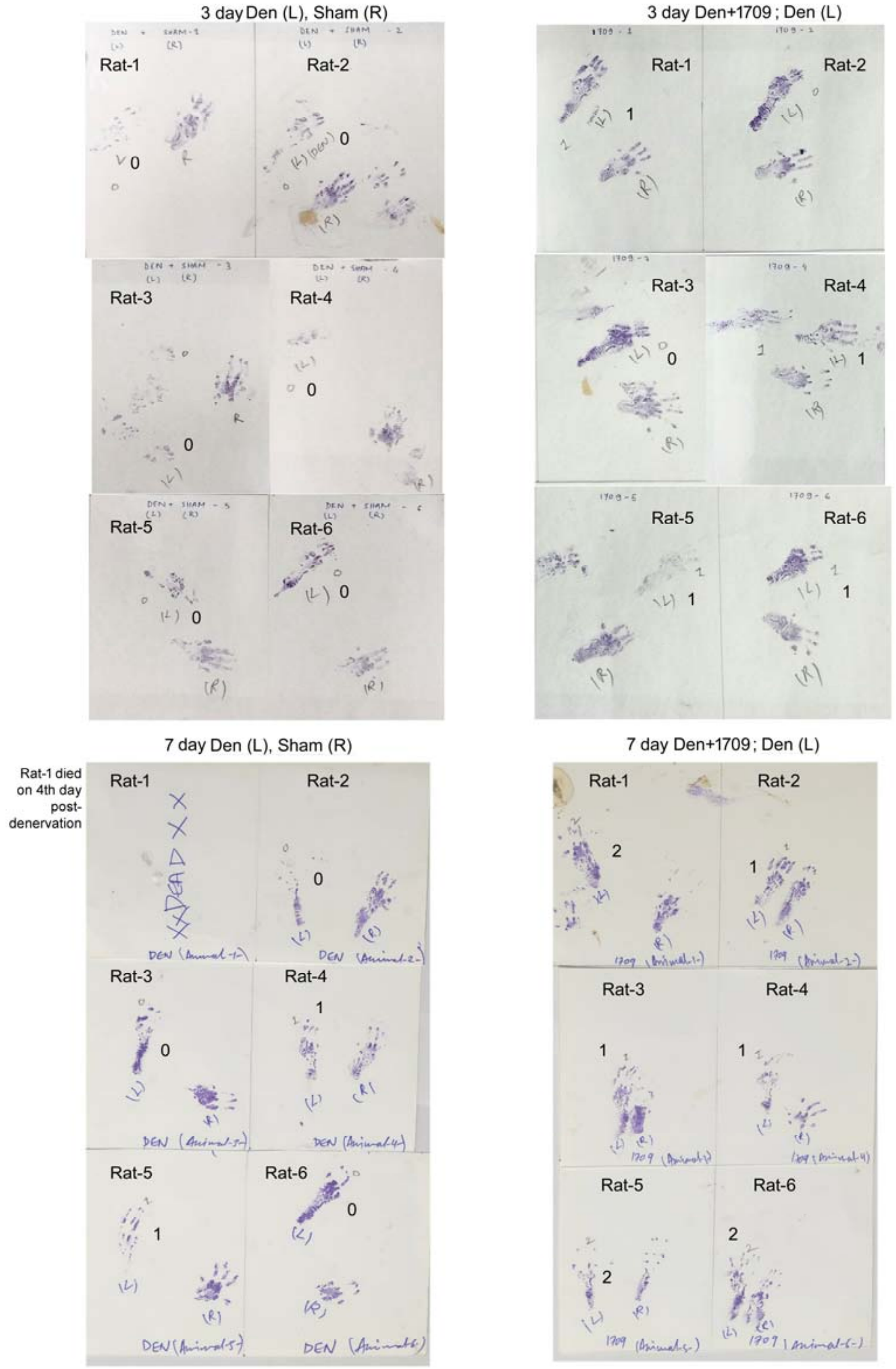

Supplementary Figure S7. Paw prints taken on 3rd day and 7th day, post-denervation. Paw print of each rat is labeled along with their respective scores. Left hind limb in each case was

denervated and the right limb served as sham and was considered as benchmark (score: 2, indicating complete opening of paws). Corresponding graphs are in **main Figure 6F**.

Supplementary Figure S8

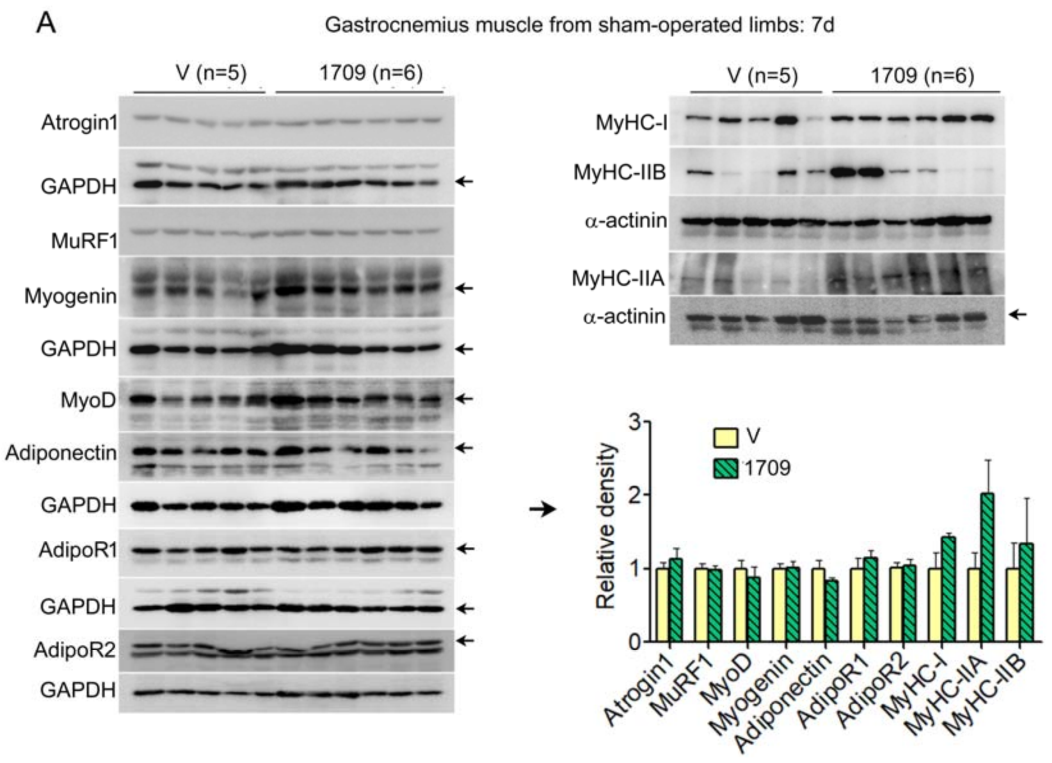

Supplementary Figure S8. CDRI-1709s treatment does not significantly alter expressions of the proteins examined in GN muscles from sham-operated limbs. A. Immunoblot panels represent expression of indicated proteins in GN muscles from sham-operated limbs of vehicle (n=5) or 1709 (n=6) -treated rats corresponding to the denervation experiment (**main Figure 6**). Graph represents densitometric analysis (mean ± SEM). MyHC-I, MyHC-IIA, and MyHC-IIB were normalized with α-actinin, and the rest were normalized with GAPDH. Statistical analysis was performed by unpaired, two-tailed Student's t-test, except for AdipoR2, MyHC-I, MyHC-IIA and MyHC-IIB, where Mann-Whitney test was used. F test was used to assess intra-group variances.

Supplementary references

S1. Ziemke F, Mantzoros CS. Adiponectin in insulin resistance: lessons from translational research. Am J Clin Nutr. 2010;91:258S-61S. doi:10.3945/ajcn.2009.28449C
S0002-9165(23)01621-0 [pii]
28449C [pii]

S2. Achari AE, Jain SK. Adiponectin, a Therapeutic Target for Obesity, Diabetes, and Endothelial Dysfunction. Int J Mol Sci. 2017;18:doi:10.3390/ijms18061321
1321
ijms18061321 [pii]

S3. Nguyen TMD. Adiponectin: Role in Physiology and Pathophysiology. Int J Prev Med. 2020;11:136. doi:10.4103/ijpvm.IJPVM_193_20
136
IJPVM-11-136 [pii]

S4. Krause MP, Liu Y, Vu V, Chan L, Xu A, Riddell MC, et al. Adiponectin is expressed by skeletal muscle fibers and influences muscle phenotype and function. Am J Physiol Cell Physiol. 2008;295:C203-12. doi:10.1152/ajpcell.00030.2008
00030.2008 [pii]
C-00030-2008 [pii]

S5. Straub LG, Scherer PE. Metabolic Messengers: Adiponectin. Nat Metab. 2019;1:334-9. doi:10.1038/s42255-019-0041-z
10.1038/s42255-019-0041-z [pii]

S6. Delaigle AM, Jonas JC, Bauche IB, Cornu O, Brichard SM. Induction of adiponectin in skeletal muscle by inflammatory cytokines: in vivo and in vitro studies. Endocrinology. 2004;145:5589-97. doi:en.2004-0503 [pii]
10.1210/en.2004-0503

S7. Langen RC, Gosker HR, Remels AH, Schols AM. Triggers and mechanisms of skeletal muscle wasting in chronic obstructive pulmonary disease. Int J Biochem Cell Biol. 2013;45:2245-56. doi:S1357-2725(13)00199-4 [pii]
10.1016/j.biocel.2013.06.015
S8. Martinez-Luna N, Orea-Tejeda A, Gonzalez-Islas D, Flores-Cisneros L, Keirns-Davis C, Sanchez-Santillan R, et al. Association between body composition, sarcopenia and pulmonary function in chronic obstructive pulmonary disease. BMC Pulm Med. 2022;22:106. doi:10.1186/s12890-022-01907-1

S9. Cao RY, Li J, Dai Q, Li Q, Yang J. Muscle Atrophy: Present and Future. Adv Exp Med Biol. 2018;1088:605-24. doi:10.1007/978-981-13-1435-3_29

S10. Hong Y, Lee JH, Jeong KW, Choi CS, Jun HS. Amelioration of muscle wasting by glucagon-like peptide-1 receptor agonist in muscle atrophy. J Cachexia Sarcopenia Muscle. 2019;10:903-18. doi:10.1002/jcsm.12434
JCSM12434 [pii]

S11. Huang L, Li M, Deng C, Qiu J, Wang K, Chang M, et al. Potential Therapeutic Strategies for Skeletal Muscle Atrophy. Antioxidants (Basel). 2022;12:doi:10.3390/antiox12010044
44
antiox12010044 [pii]
antioxidants-12-00044 [pii]

S12. Sente T, Van Berendoncks AM, Hoymans VY, Vrints CJ. Adiponectin resistance in skeletal muscle: pathophysiological implications in chronic heart failure. J Cachexia Sarcopenia Muscle. 2016;7:261-74. doi:10.1002/jcsm.12086
JCSM12086 [pii]

S13. Liu Y, Sweeney G. Adiponectin action in skeletal muscle. Best Pract Res Clin Endocrinol Metab. 2014;28:33-41. doi:S1521-690X(13)00115-2 [pii]
10.1016/j.beem.2013.08.003

S14. Abou-Samra M, Selvais CM, Boursereau R, Lecompte S, Noel L, Brichard SM. AdipoRon, a new therapeutic prospect for Duchenne muscular dystrophy. J Cachexia Sarcopenia Muscle. 2020;11:518-33. doi:10.1002/jcsm.12531
JCSM12531 [pii]

S15. Balasubramanian P, Schaar AE, Gustafson GE, Smith AB, Howell PR, Greenman A, et al. Adiponectin receptor agonist AdipoRon improves skeletal muscle function in aged mice. Elife. 2022;11:doi:10.7554/eLife.71282
e71282
71282 [pii]

S16. Fernandez-Marcos PJ, Auwerx J. Regulation of PGC-1alpha, a nodal regulator of mitochondrial biogenesis. Am J Clin Nutr. 2011;93:884S-90. doi:10.3945/ajcn.110.001917
S0002-9165(23)02168-8 [pii]
001917 [pii]

S17. Fiaschi T, Giannoni E, Taddei ML, Chiarugi P. Globular adiponectin activates motility and regenerative traits of muscle satellite cells. PLoS One. 2012;7:e34782. doi:10.1371/journal.pone.0034782
e34782
PONE-D-11-24656 [pii]

S18. Ingelsson E, Arnlov J, Zethelius B, Vasan RS, Flyvbjerg A, Frystyk J, et al. Associations of serum adiponectin with skeletal muscle morphology and insulin sensitivity. J Clin Endocrinol Metab. 2009;94:953-7. doi:10.1210/jc.2008-1772
jc.2008-1772 [pii]

S19. Jortay J, Senou M, Abou-Samra M, Noel L, Robert A, Many MC, et al. Adiponectin and skeletal muscle: pathophysiological implications in metabolic stress. Am J Pathol. 2012;181:245-56. doi:10.1016/j.ajpath.2012.03.035
S0002-9440(12)00324-0 [pii]
S20. Fiaschi T, Tedesco FS, Giannoni E, Diaz-Manera J, Parri M, Cossu G, et al. Globular adiponectin as a complete mesoangioblast regulator: role in proliferation, survival, motility, and skeletal muscle differentiation. Mol Biol Cell. 2010;21:848-59. doi:E09-04-0310 [pii]
3571077 [pii]
10.1091/mbc.e09-04-0310

S21. Khan MP, Singh AK, Joharapurkar AA, Yadav M, Shree S, Kumar H, et al. Pathophysiological Mechanism of Bone Loss in Type 2 Diabetes Involves Inverse Regulation of Osteoblast Function by PGC-1alpha and Skeletal Muscle Atrogenes: AdipoR1 as a Potential Target for Reversing Diabetes-Induced Osteopenia. Diabetes. 2015;64:2609-23. doi:10.2337/db14-1611
db14-1611 [pii]

S22. Selvais CM, Davis-Lopez de Carrizosa MA, Versele R, Dubuisson N, Noel L, Brichard SM, et al. Challenging Sarcopenia: Exploring AdipoRon in Aging Skeletal Muscle as a Healthspan-Extending Shield. Antioxidants (Basel). 2024;13:doi:10.3390/antiox13091073
1073
antiox13091073 [pii]
antioxidants-13-01073 [pii]

S23. Schneider CA, Rasband WS, Eliceiri KW. NIH Image to ImageJ: 25 years of image analysis. Nat Methods. 2012;9:671-5. doi:10.1038/nmeth.2089

S24. Pal China S, Sanyal S, Chattopadhyay N. Adiponectin signaling and its role in bone metabolism. Cytokine. 2018;112:116-31. doi:S1043-4666(18)30260-6 [pii]
10.1016/j.cyto.2018.06.012

S25. Iwabu M, Yamauchi T, Okada-Iwabu M, Sato K, Nakagawa T, Funata M, et al. Adiponectin and AdipoR1 regulate PGC-1alpha and mitochondria by Ca(2+) and AMPK/SIRT1. Nature. 2010;464:1313-9. doi:10.1038/nature08991
nature08991 [pii]

S26. Wu Z, Puigserver P, Andersson U, Zhang C, Adelmant G, Mootha V, et al. Mechanisms controlling mitochondrial biogenesis and respiration through the thermogenic coactivator PGC-1. Cell. 1999;98:115-24. doi:S0092-8674(00)80611-X [pii]
10.1016/S0092-8674(00)80611-X

S27. Wang Y, Pessin JE. Mechanisms for fiber-type specificity of skeletal muscle atrophy. Curr Opin Clin Nutr Metab Care. 2013;16:243-50. doi:10.1097/MCO.0b013e328360272d

S28. Pal S, Sharma S, Porwal K, Riyazuddin M, Kulkarni C, Chattopadhyay S, et al. Oral Administration of Isovitexin, a Naturally Occurring Apigenin Derivative Showed Osteoanabolic Effect in Ovariectomized Mice: A Comparative Study with Teriparatide. Calcif Tissue Int. 2022;111:196-210. doi:10.1007/s00223-022-00979-9
10.1007/s00223-022-00979-9 [pii]

S29. Shinohara I, Kataoka T, Mifune Y, Inui A, Sakata R, Nishimoto H, et al. Influence of adiponectin and inflammatory cytokines in fatty degenerative atrophic muscle. Sci Rep. 2022;12:1557. doi:10.1038/s41598-022-05608-x
1557
10.1038/s41598-022-05608-x [pii]
5608 [pii]

S30. Ji Y, Li M, Chang M, Liu R, Qiu J, Wang K, et al. Inflammation: Roles in Skeletal Muscle Atrophy. Antioxidants (Basel). 2022;11:doi:10.3390/antiox11091686
1686
antiox11091686 [pii]
antioxidants-11-01686 [pii]
