# Supplementary material for: Selective Small‐Molecule AdipoR1 Agonist 3‐Hydroxy Pterocarpan Salt (CDRI‐1709S) Ameliorates Skeletal Muscle Atrophy
Source: J Cachexia Sarcopenia Muscle. 2026 Jul 21;17(4):e70328. doi: 10.1002/jcsm.70328 (PMC13387834; doi:10.1002/jcsm.70328)

Supplementary Materials and Methods

General procedure for the synthesis of sodium 6a,11a-dihydro-6H-benzofuro[3,2-c]chromen-3-olate (CDRI-1709S):

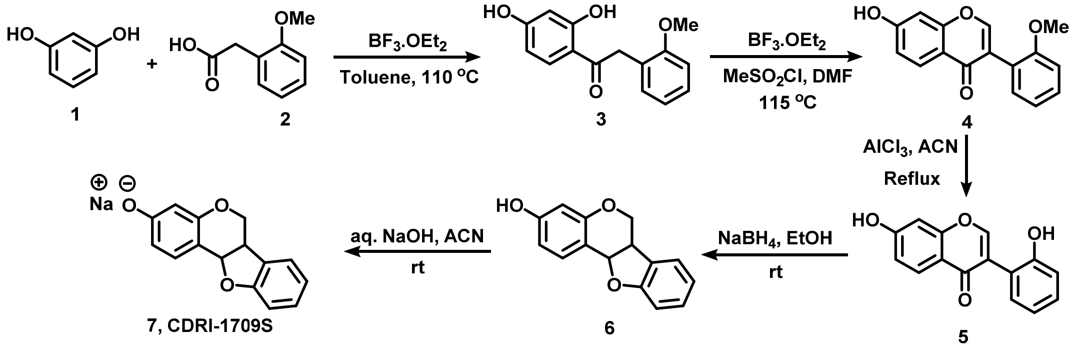

Scheme-1. Synthesis of sodium 6a,11a-dihydro-6H-benzofuro[3,2-c]chromen-3-olate **(CDRI-1709S)**.

Compound **6** was prepared according to the procedure demonstrated in our previous report (Singh et al., 2023), (Scheme-1). Briefly, the acylation reaction of 2-(2-methoxyphenyl)acetic acid and resorcinol in presence of Lewis acid, $BF_3.OEt_2$ produced aromatic ketone **3** in 85% yield. This compound **3,** on further treatment with DMF and methanesulfonyl chloride, formed substituted isoflavone derivative **7**-hydroxy-3-(2-methoxyphenyl)-4H-chromen-4-one (**4**). Next, on demethylation of **4** using aluminium trichloride in ACN reflux, lead to the formation of 7-hydroxy-3-(2-hydroxyphenyl)-4H-chromen-4-one **(5)** in 70% yield. Subsequent reduction followed by intramolecular cyclization of **5** using $NaBH_4$ furnished 6a,11a-dihydro-6H-benzofuro[3,2-c]chromen-3-ol (**6**) in 63% yield. Finally, the treatment of compound **6** with aq. NaOH solution at room temperature resulted in the formation of water-soluble sodium 6a,11a-dihydro-6H-benzofuro[3,2-c]chromen-3-olate (**CDRI-1709S)** in 89% yield.

Method of synthesis:

Synthesis of 1-(2,4-dihydroxyphenyl)-2-(2-methoxyphenyl)ethan-1-one (3):

In a 250 mL round-bottom flask, charged with resorcinol **1** (5 g, 1 equiv) and 2-(2-methoxyphenyl) acetic acid **2** (8.3 g, 1.1 equiv), toluene (100 mL) was added. The resulting mixture was cooled to 0 °C and $BF_3.OEt_2$ (11.7 mL, 2 equiv) was added dropwise. The reaction mixture was then heated under reflux for 4 h. After this, the reaction was allowed to cool to room temperature and concentrated under reduced pressure. The residue was quenched with water and neutralized by the addition of a saturated solution of $NaHCO_3$. The resulting precipitate was filtered and washed with chlorobenzene to afford the desired compound **3** (10 g, 85% yield). Mp: 158–160 °C; R_f 0.52 (3:2 Hexane:EtOAc); ^{1}H NMR (500 MHz, DMSO-d_6): δ 12.47 (s, 1H), 10.63 (s, 1H), 7.92 (d, J = 8.8 Hz, 1H), 7.25 (td, J = 7.8, 1.7 Hz, 1H), 7.16 (dd, J = 7.4, 1.7 Hz, 1H), 6.98 (d, J = 8.2 Hz, 1H), 6.90 (td, J = 7.4, 1.2 Hz, 1H), 6.40 (dd, J = 8.8, 2.4 Hz, 1H), 6.26 (d, J = 2.3 Hz, 1H), 4.24 (s, 2H), 3.72 (s, 3H); ^{13}C NMR (125 MHz, DMSO-d_6): δ 201.9,

164.8, 164.3, 157.1, 133.0, 131.2, 128.2, 123.5, 120.2, 112.4, 110.7, 108.2, 102.4, 55.4, 39.0; HRMS (ESI) Calcd for $C_{15}H_{15}O_4$ $[M+H]^+$: 259.0970, found: 259.0970.

Synthesis of 7-hydroxy-3-(2-methoxyphenyl)-4H-chromen-4-one (4). To a solution of compound **3** (8 g, 1 equiv) in dry DMF (100 mL) at 0 °C was added $BF_3.OEt_2$ (7.9 mL, 2 equiv) dropwise over 5 mins. After 30 mins, methanesulfonyl chloride (4.7 mL, 2 equiv) was added, and the reaction mixture was allowed to stir at 115 °C. Upon completion, the reaction was poured into 300 mL of ice-cold water and neutralized by the addition of saturated sodium bicarbonate solution. The precipitate was filtered, and the crude product was purified using silica gel column chromatography to afford the desired compound **4** (5.5 g, 66% yield). Mp: 228-230 °C; R_f 0.23 (3:2 Hexane:EtOAc); ^{1}H NMR (400 MHz, DMSO-d_6) δ 10.70 (s, 1H), 8.17 (s, 1H), 7.93 (d, J = 8.7 Hz, 1H), 7.40 – 7.34 (m, 1H), 7.22 (dd, J = 7.4, 1.8 Hz, 1H), 7.07 (dd, J = 8.4, 1.0 Hz, 1H), 6.99 (td, J = 7.4, 1.0 Hz, 1H), 6.94 (dd, J = 8.7, 2.3 Hz, 1H), 6.87 (d, J = 2.2 Hz, 1H), 3.71 (s, 3H); ^{13}C NMR (100 MHz, DMSO-d_6) δ 174.2, 162.5, 157.6, 157.5, 154.0, 131.6, 129.6, 127.2, 121.9, 121.2, 120.1, 116.5, 115.1, 111.3, 102.2, 55.5; HRMS (ESI) Calcd for $C_{16}H_{13}O_4$ $[M+H]^+$: 269.0814, found: 269.0820.

Synthesis of 7-hydroxy-3-(2-hydroxyphenyl)-4H-chromen-4-one (5). To a solution of compound **4** (5 g, 1 equiv) in ACN (150 mL) at 0 °C was added anhydrous $AlCl_3$ (14.9 g, 6 equiv) in portions. The reaction mixture was then transferred to heating and allowed to reflux for 24 h. After completion, the reaction mixture was cooled to room temperature and quenched by the addition of water. The precipitate was filtered, and the crude product was purified using silica gel column chromatography to afford the desired **5** (3.3 g, 70% yield). Mp: 208–210 °C; R_f 0.32 (3:2 Hexane:EtOAc); ^{1}H NMR (500 MHz, DMSO-d_6): δ 10.79 (s, 1H), 9.35 (s, 1H), 8.21 (s, 1H), 7.95 (d, J = 8.7 Hz, 1H), 7.23 – 7.15 (m, 2H), 6.94 (dd, J = 8.7, 2.3 Hz, 1H), 6.92 – 6.86 (m, 2H), 6.86-6.80 (m, 1H); ^{13}C NMR (125 MHz, DMSO-d_6): δ 174.7, 162.5, 157.5, 155.5, 154.5, 131.7, 129.2, 127.2, 121.8, 119.4, 118.7, 116.6, 115.8, 115.1, 102.1; HRMS (ESI) Calcd for $C_{15}H_{11}O_4$ $[M+H]^+$: 255.0657, found: 255.0663.

Synthesis of 6a,11a-dihydro-6H-benzofuro[3,2-c]chromen-3-ol (6). In a 100 mL round-bottom flask, compound **5** (2.7 g, 1 equiv) was dissolved in ethanol and cooled to 0 °C. To the solution of **5**, solid $NaBH_4$ (2.4 g, 6 equiv) was added in portions over 10 mins. The cooling bath was then removed, and the reaction was allowed to stir at room temperature for 7 h. The solvent was removed under reduced pressure, and the reaction was quenched by the addition of water. The reaction was then neutralized with dil. HCl, and the resulting precipitate was filtered. The crude product was purified using silica gel column chromatography to afford the desired compound **6** (1.6 g, 63% yield). Mp: 142–143 °C; R_f 0.63 (3:2 Hexane: EtOAc); ^{1}H NMR (500 MHz, DMSO-d_6): δ 9.63 (s, 1H), 7.39-7.34 (m, 1H), 7.28 (d, J = 8.4 Hz, 1H), 7.14 (td, J = 7.7, 1.4 Hz, 1H), 6.88 (td, J = 7.4, 1.0 Hz, 1H), 6.78 (d, J = 7.9 Hz, 1H), 6.48 (dd, J = 8.4, 2.4 Hz, 1H), 6.26 (d, J = 2.4 Hz, 1H), 5.55 (d, J = 7.0 Hz, 1H), 4.31 – 4.24 (m, 1H), 3.74 – 3.63 (m, 2H); ^{13}C NMR (125 MHz, DMSO-d_6): δ 159.0, 158.7, 156.3, 132.1, 128.8, 127.6, 125.1, 120.5, 111.2, 109.7, 109.5, 102.8, 77.2, 65.7, 39.5; HRMS (ESI) Calcd for $C_{15}H_{13}O_3$ $[M+H]^+$: 241.0865, found: 241.0863.

Purity analysis of 6a,11a-dihydro-6H-benzofuro[3,2-c]chromen-3-ol (6) :

The purity of the compound **6** was analyzed by a high-performance liquid chromatographic method, on a reverse-phase Luna 5 mm C18(2) 100 Å LC column, using an (1:1) ACN/Water eluting system with flow rate 0.400 mL/min. The purity of 6a,11a-dihydro-6H-benzofuro[3,2-c]chromen-3-ol (**6**) was 99.81 %.

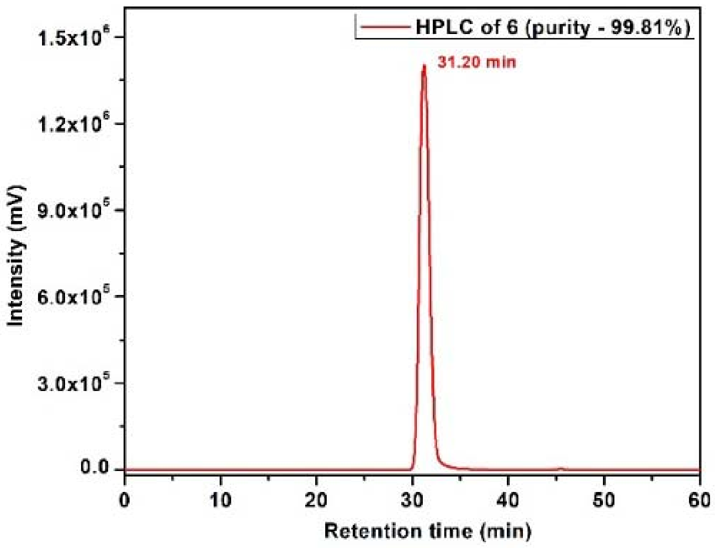

Assessment of purity: HPLC of compound **6**.

Synthesis of sodium 6a,11a-dihydro-6H-benzofuro[3,2-c]chromen-3-olate (CDRI-1709S): To a solution of compound **6** (100 mg, 1 equiv) in ACN (5 mL) was added aq. NaOH (33 mg, 2 equiv) solution at room temperature and stirred for 15 mins. The precipitate was filtered and washed with acetonitrile to furnish the desired compound **CDRI-1709S** (98 mg, 89% yield). ^{1}H NMR (500 MHz, D_2O): δ 7.38 (d, J = 7.4 Hz, 1H), 7.26 – 7.19 (m, 2H), 6.99 (t, J = 7.5 Hz, 1H), 6.82 (d, J = 8.0 Hz, 1H), 6.39 (dd, J = 8.4, 2.4 Hz, 1H), 6.10 (d, J = 2.4 Hz, 1H), 5.62 (d, J = 7.4 Hz, 1H), 4.25 (dd, J = 11.1, 4.4 Hz, 1H), 3.82 – 3.68 (m, 2H); ^{13}C NMR (125 MHz, D_2O): δ 168.9, 158.0, 156.3, 131.8, 129.2, 127.7, 125.2, 121.5, 114.8, 109.8, 106.1, 105.1, 79.2, 66.1, 39.8; HRMS (ESI) Calcd for $C_{15}H_{13}O_3$ $[M{-}Na{+}2H]^+$: 241.0865, found: 241.0867.

NMR:

^{1}H NMR Spectrum of Compound **3** (500 MHz, DMSO-d_6)

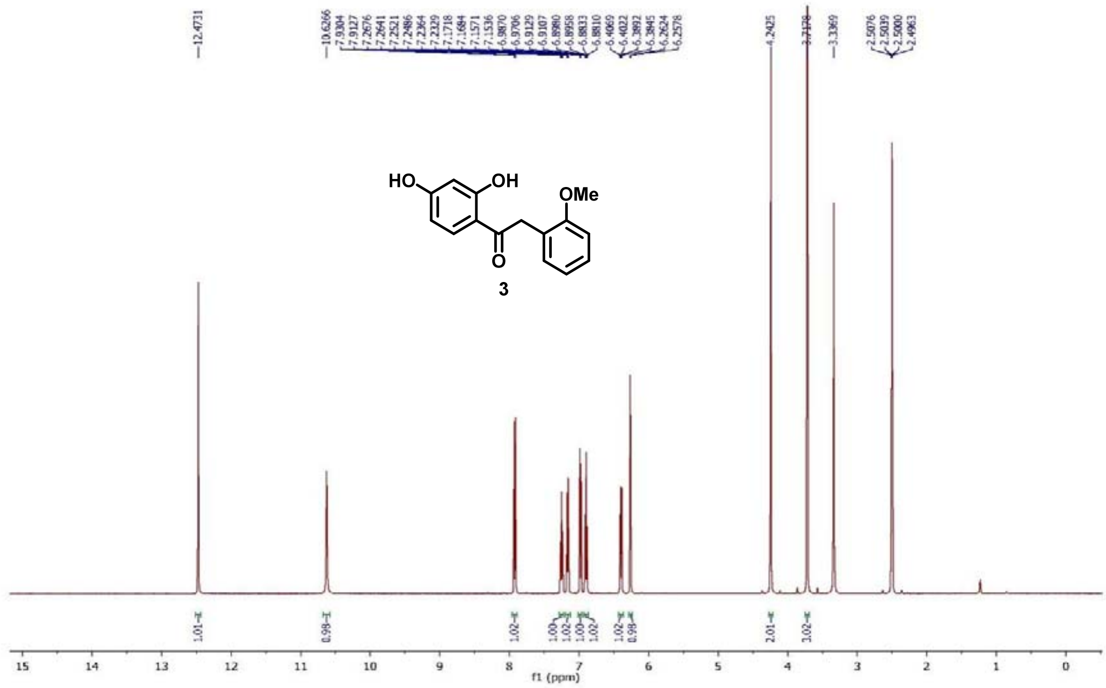

^{13}C NMR Spectrum of Compound **3** (125 MHz, DMSO-d_6)

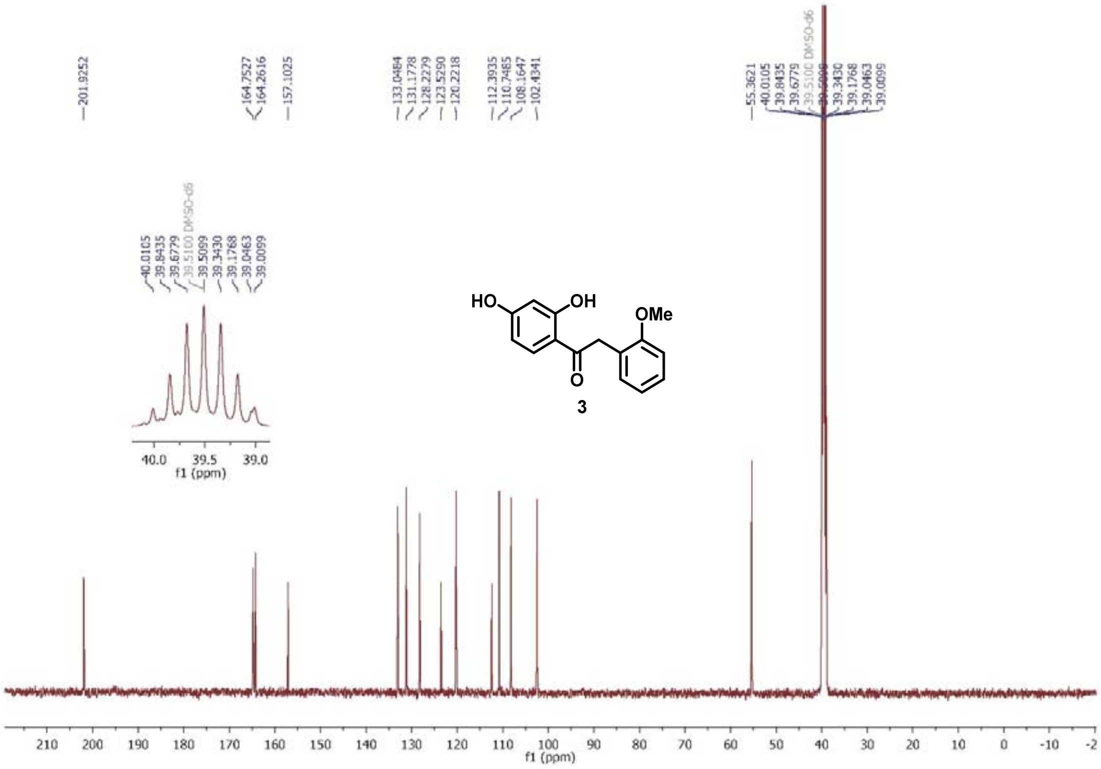

^{1}H NMR Spectrum of Compound **4** (400 MHz, DMSO-d_6)

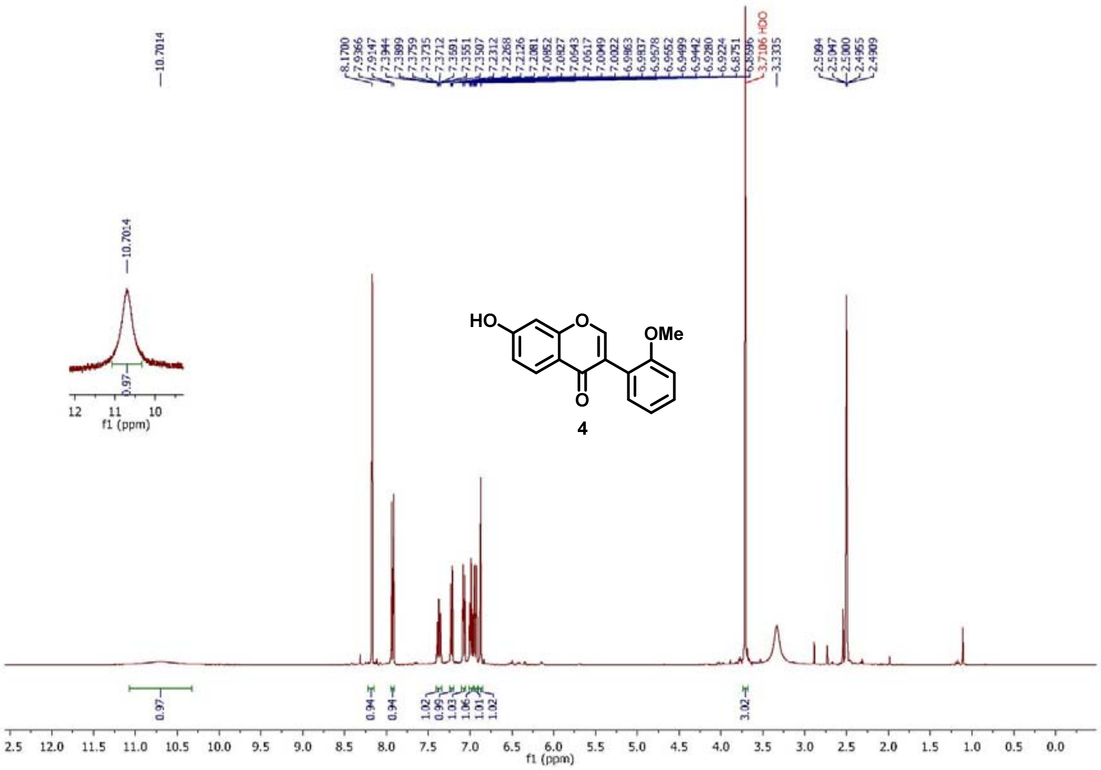

^{13}C NMR Spectrum of Compound **4** (100 MHz, DMSO-d_6)

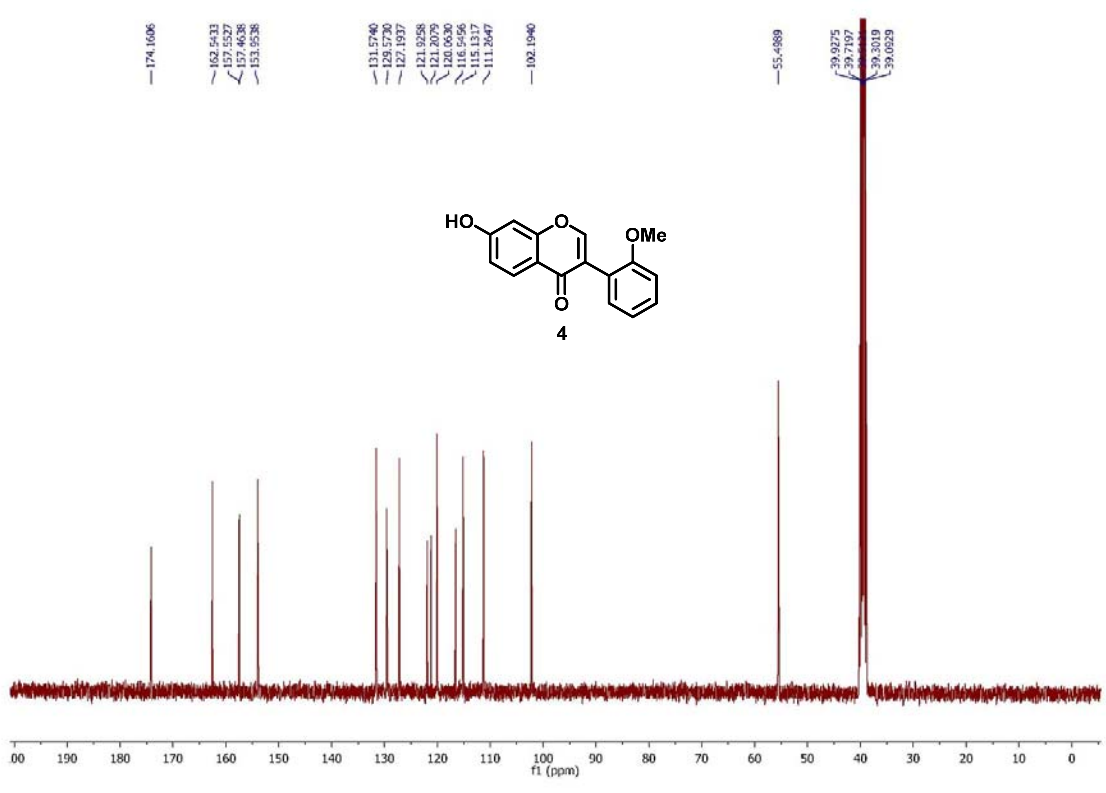

^{1}H NMR Spectrum of Compound **5** (500 MHz, DMSO-d_6)

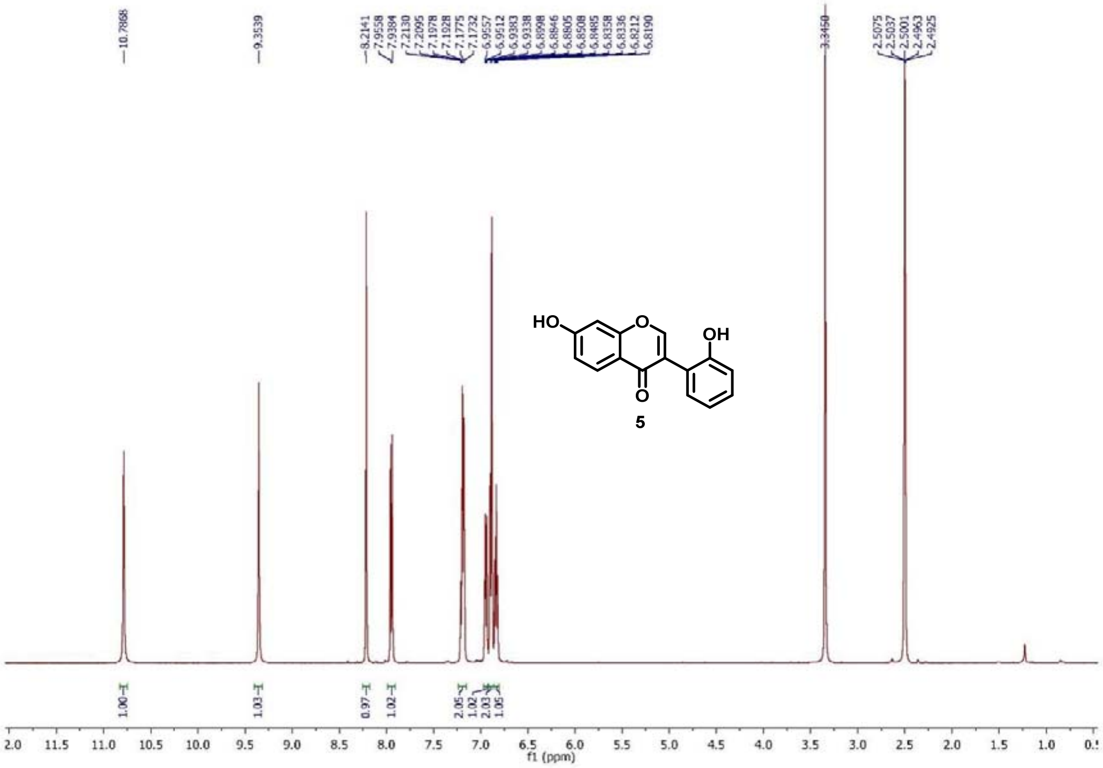

^{13}C NMR Spectrum of Compound **5** (125 MHz, DMSO-d_6)

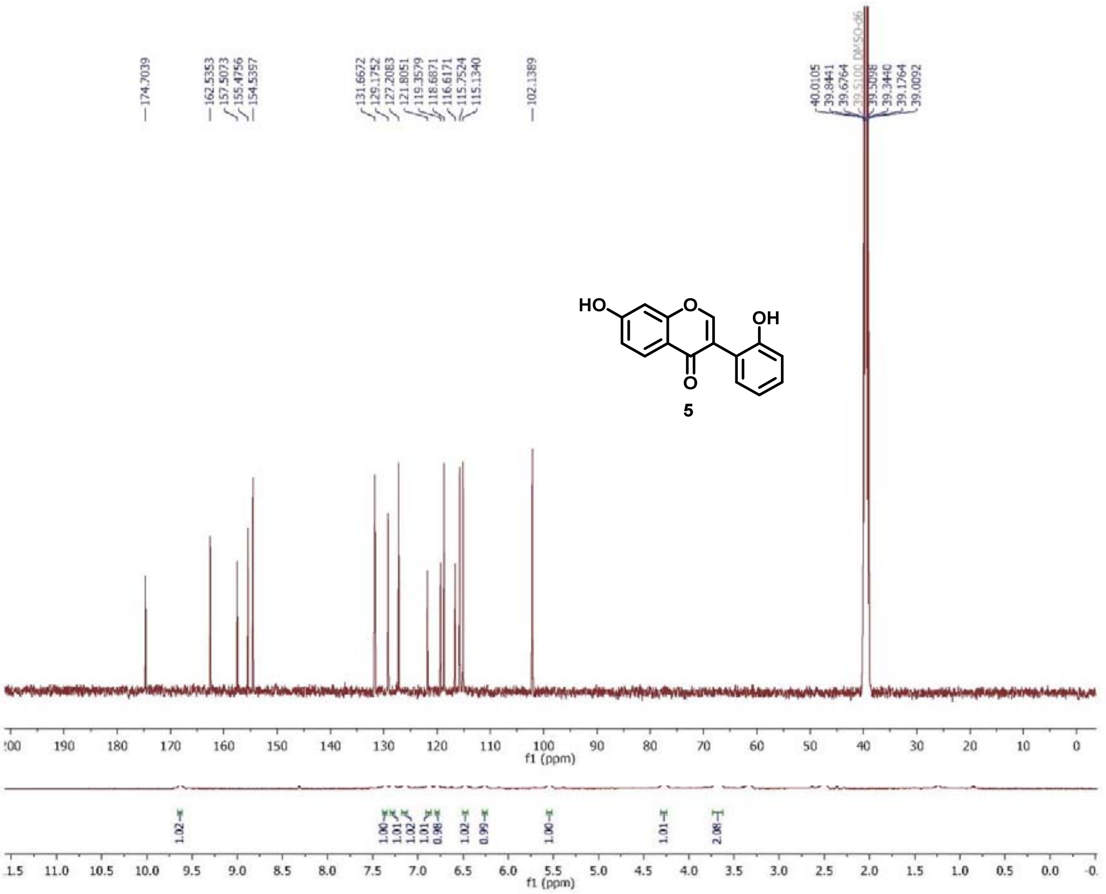

^{13}C NMR Spectrum of Compound **6** (125 MHz, DMSO-d_6)

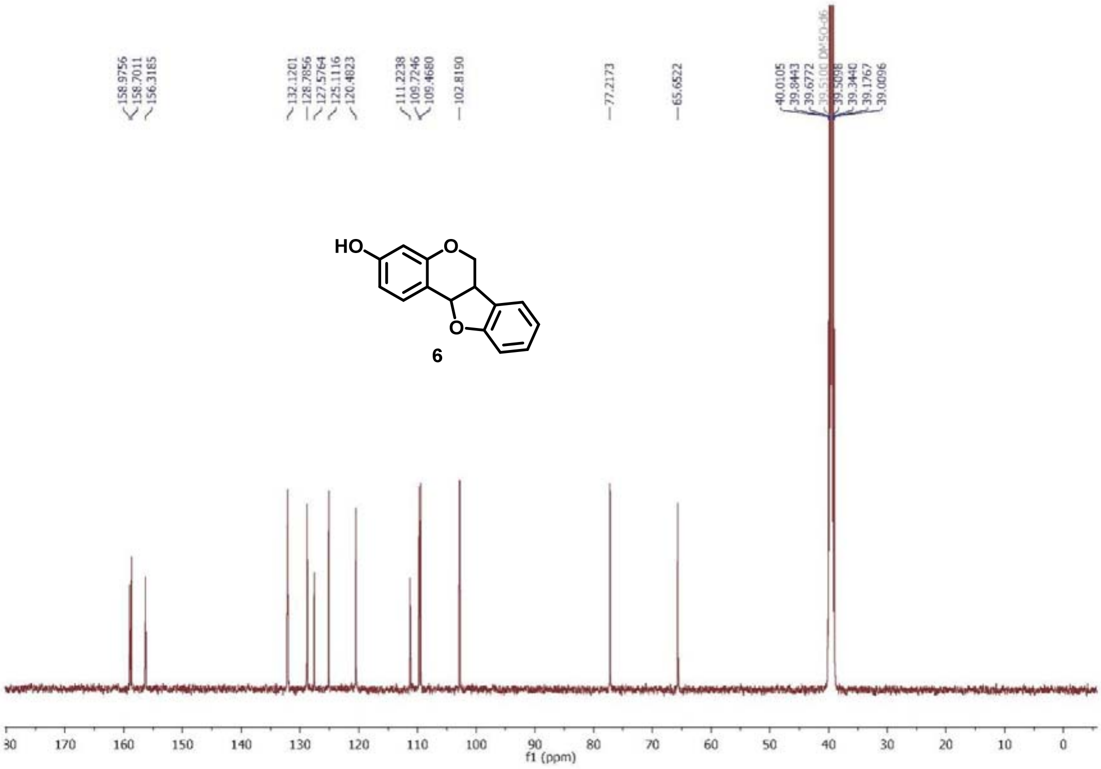

DEPT-135 NMR Spectrum of Compound **6** (125 MHz, DMSO-d_6)

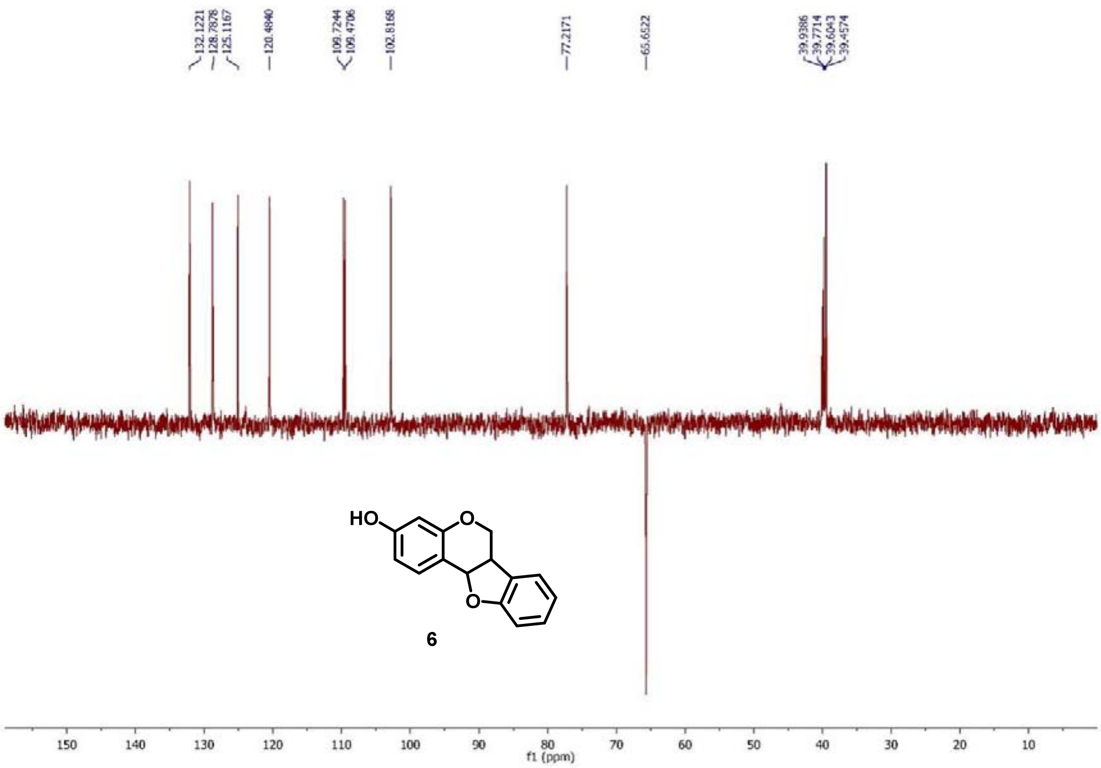

^{1}H NMR Spectrum of **CDRI-1709S** (500 MHz, D_2O)

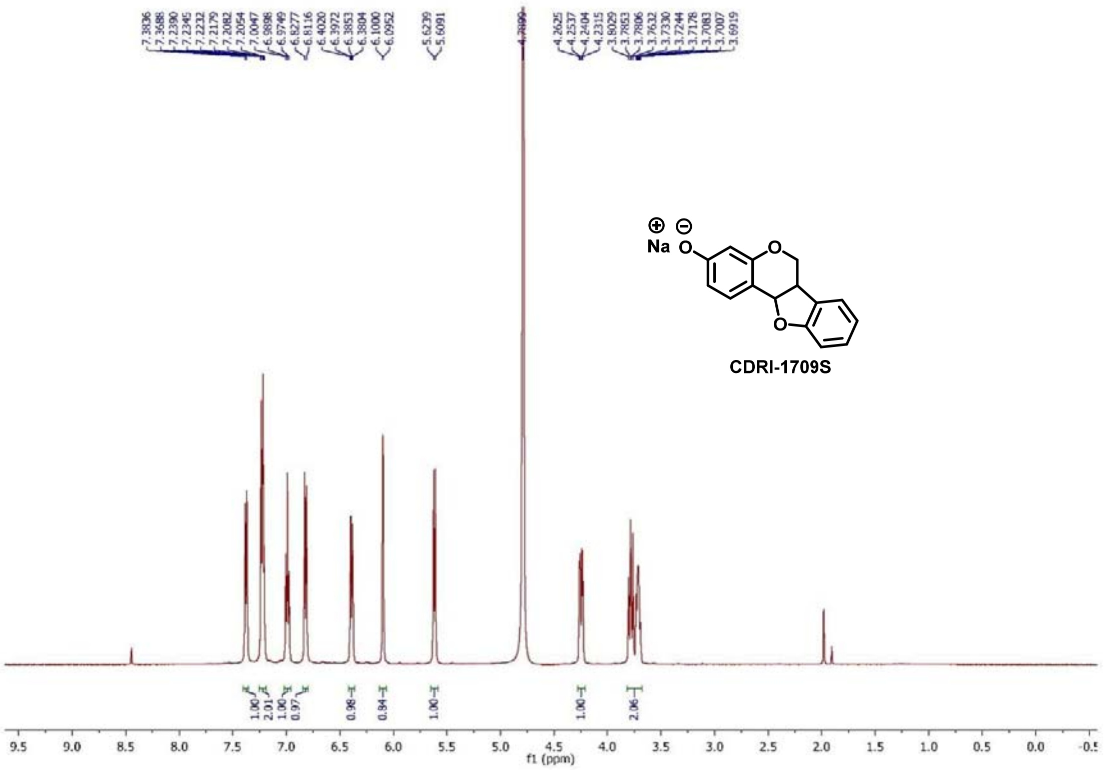

^{13}C NMR Spectrum of **CDRI-1709S** (125 MHz, D_2O)

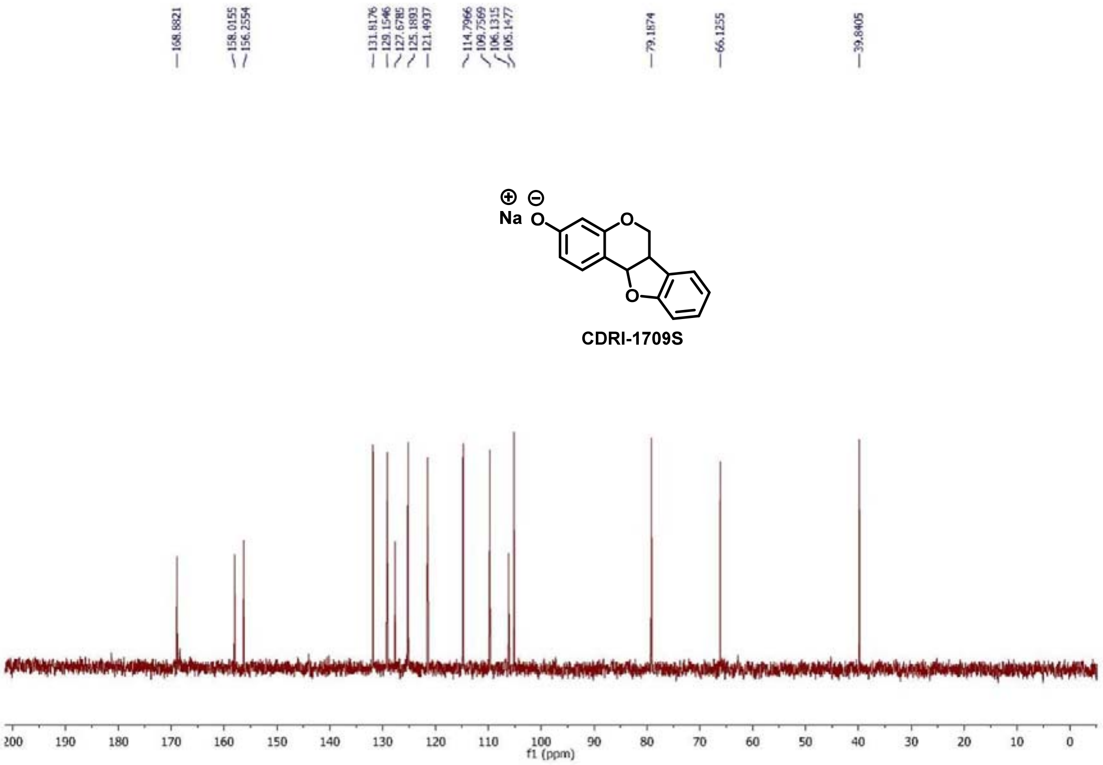

References:

1) Singh, K. B.; Awasthi, P.; Srivastava, K.; Rawat, K. S.; Rai, R.; Parveen, S.; Gautam, A. K.; Vats, R. P.; Goel, A.; Singh, D. 9-demethoxy-medicarpin: a potential bone health supplement for the management of protein deficiency-induced bone loss in growing rats. *Bioorganic & Medicinal Chemistry Letters,* **2023**, *80,* 129118.

Quantitative real-time polymerase chain reaction (QRT-PCR)

Total RNA was harvested from C2C12 myotubes or snap-frozen GN muscle tissue using a Direct-zol RNA MiniPrep kit (Zymo research, CA, USA). Total RNA (1µg) was reverse transcribed using a NeoScript cDNA synthesis kit (Genes2Me, India). SYBR Green Master mix (Applied Biosystems, MA, USA), was used for quantitative determination of relative mRNA expression using a CFX96 Real-Time PCR System (Bio-Rad, CA, USA) and ΔΔCT method, with β-actin (for myotubes) or GAPDH (for muscle tissue) used as reference genes (myotubes: n=3, muscle tissue: n=3 rats/group, in triplicates). Primer sequences are provided in the **Supplementary Table 2**.

EchoMRI

Body composition of live SD rats was analyzed using an EchoMRI-500 body composition analyzer (EchoMRI Corporation Pvt. Ltd. Singapore) on day 0, 7, and 14. Prior to analysis, the machine was calibrated by scanning columns of canola oil and distilled water provided by the manufacturer.

Figure 1

Figure 1C

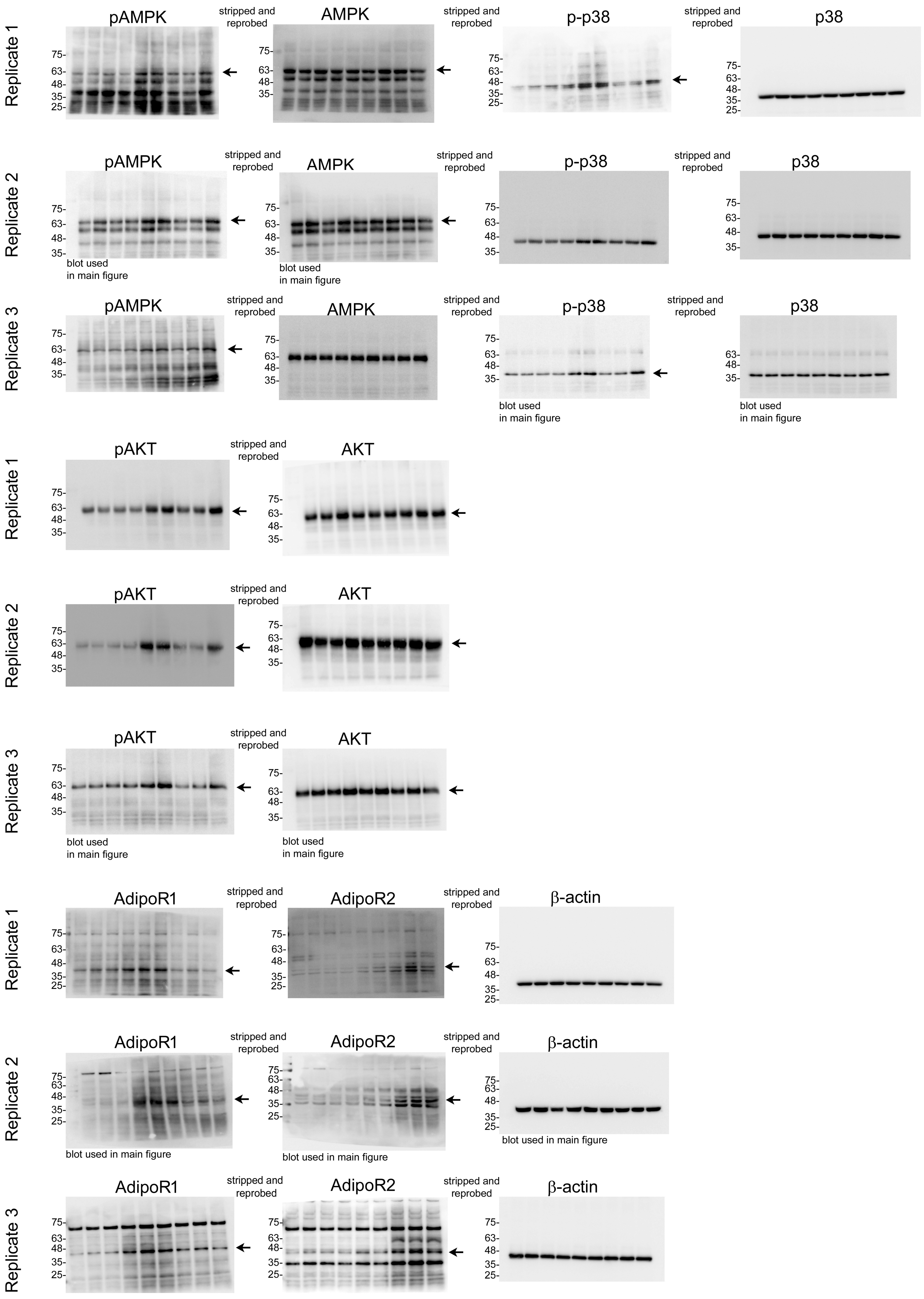

Figure 1D

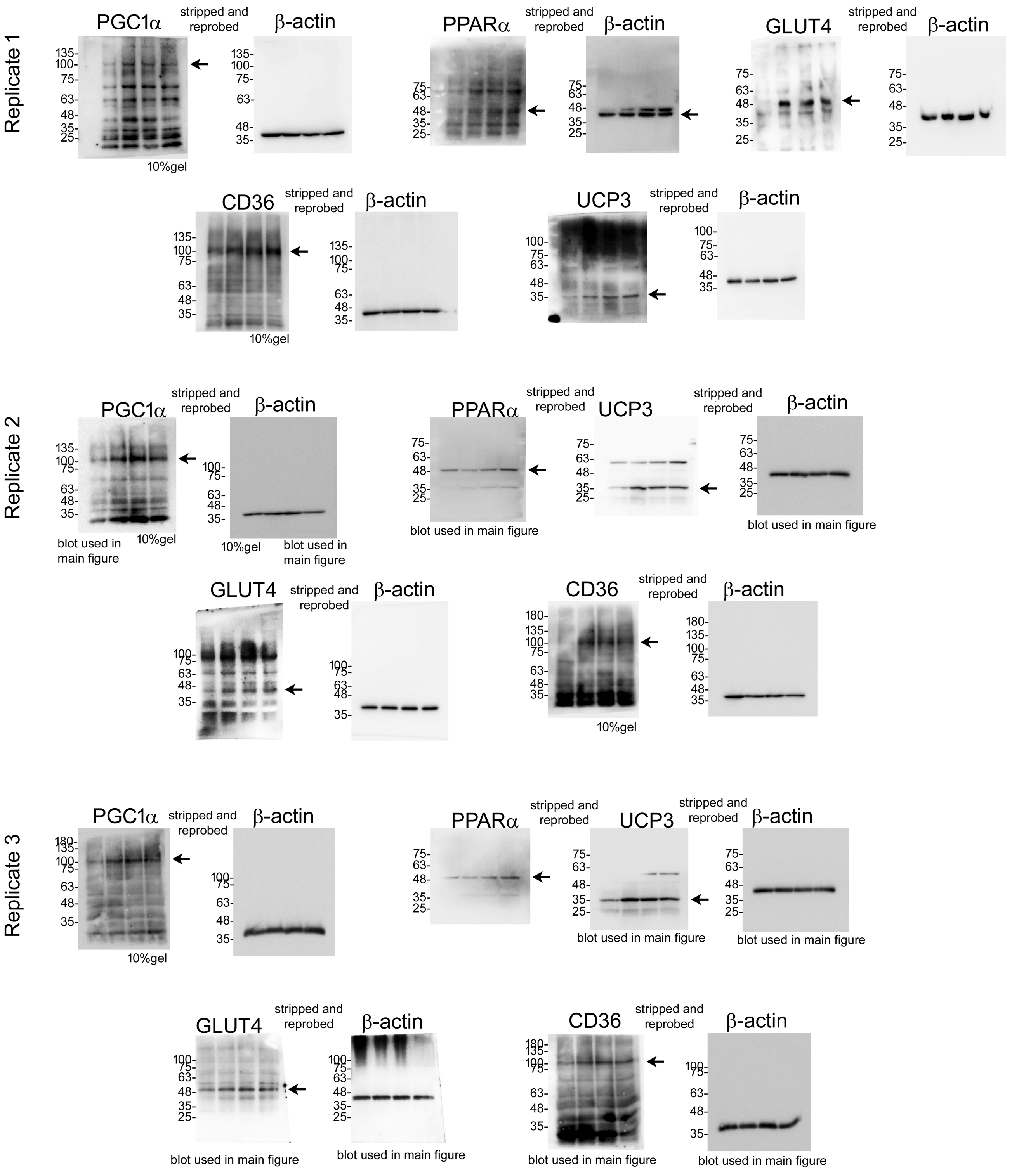
Replicate 1
PGC1α
stripped and reprobed
β-actin
10%gel
PPARα
stripped and reprobed
β-actin
GLUT4
stripped and reprobed
β-actin
CD36
stripped and reprobed
β-actin
10%gel
UCP3
stripped and reprobed
β-actin
Replicate 2
PGC1α
stripped and reprobed
β-actin
blot used in main figure
10%gel
10%gel
blot used in main figure
PPARα
stripped and reprobed
UCP3
stripped and reprobed
β-actin
blot used in main figure
blot used in main figure
GLUT4
stripped and reprobed
β-actin
CD36
stripped and reprobed
β-actin
10%gel
Replicate 3
PGC1α
stripped and reprobed
β-actin
10%gel
PPARα
stripped and reprobed
UCP3
stripped and reprobed
β-actin
blot used in main figure
blot used in main figure
GLUT4
stripped and reprobed
β-actin
blot used in main figure
blot used in main figure
CD36
stripped and reprobed
β-actin
blot used in main figure
blot used in main figure

Figure 1E-KD (set-1) -Part-A

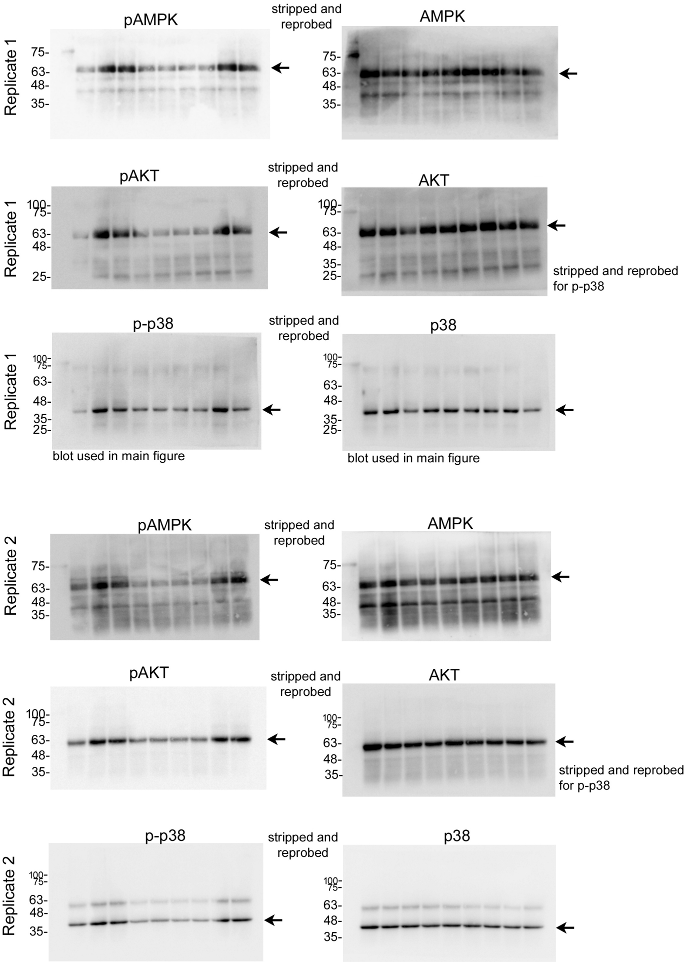

Figure 1E-KD (set1) -Part-B

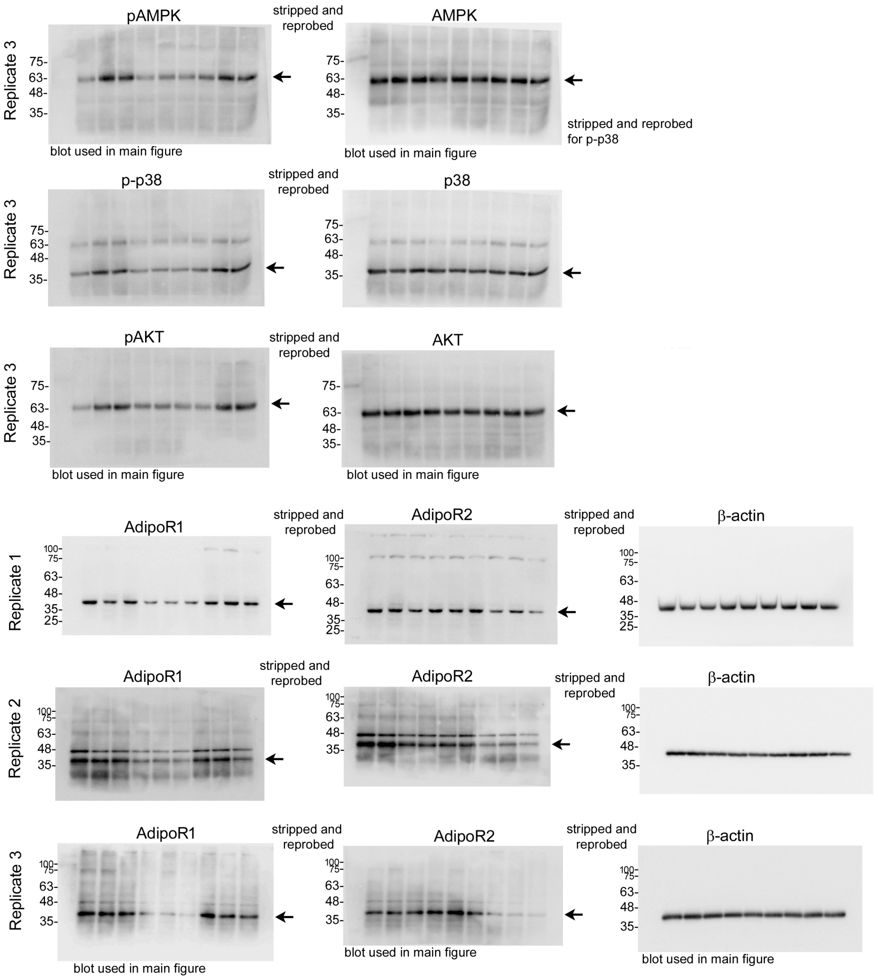

Figure 1F-KD (set-1) -Part-A

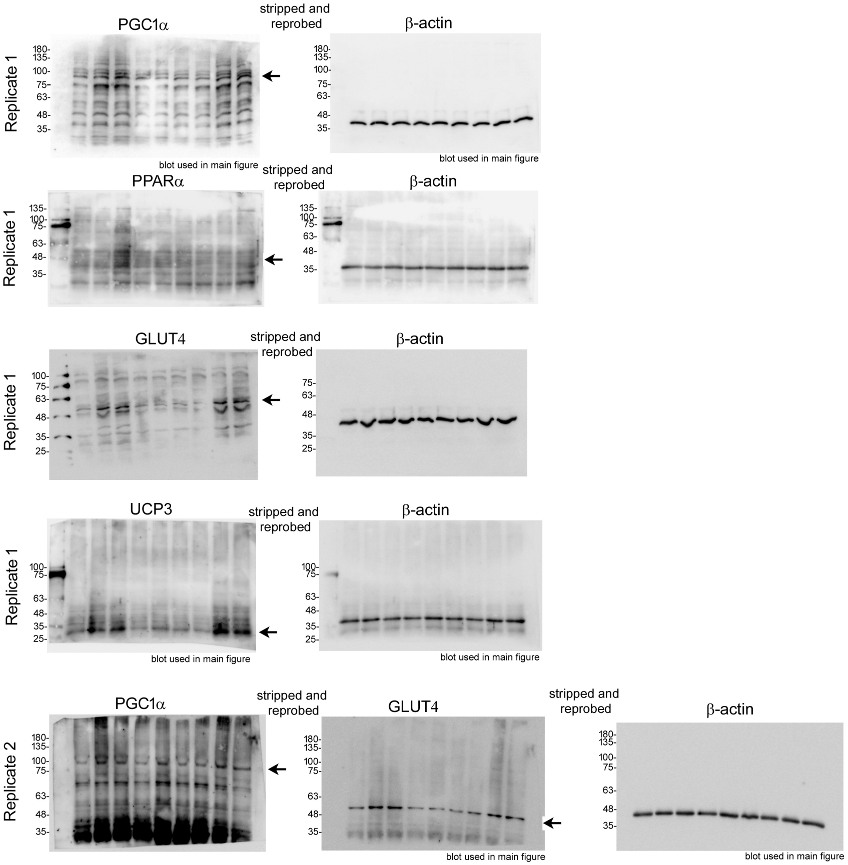

Figure 1F-KD (set1) -Part-B

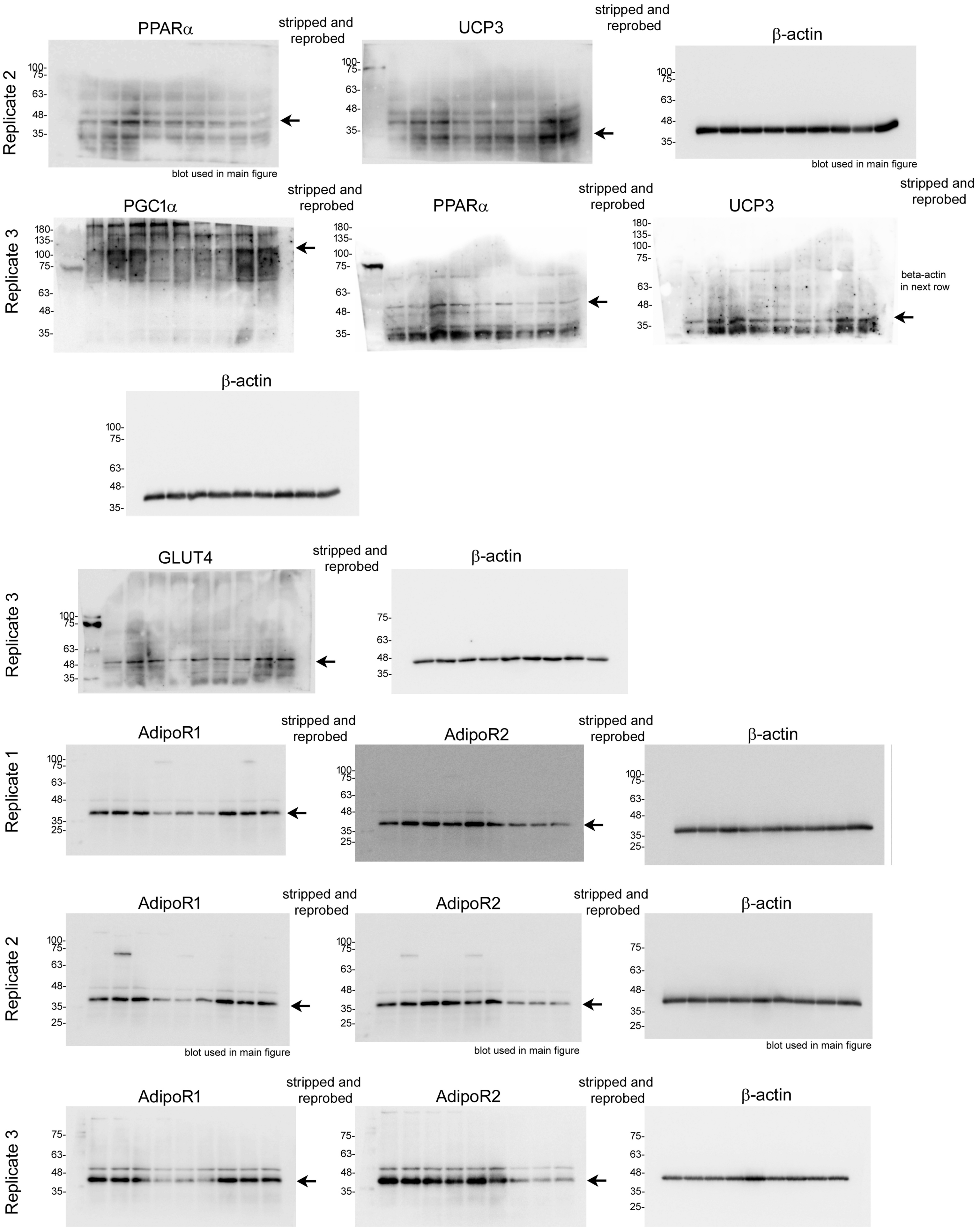

Figure 2

Figure 2B

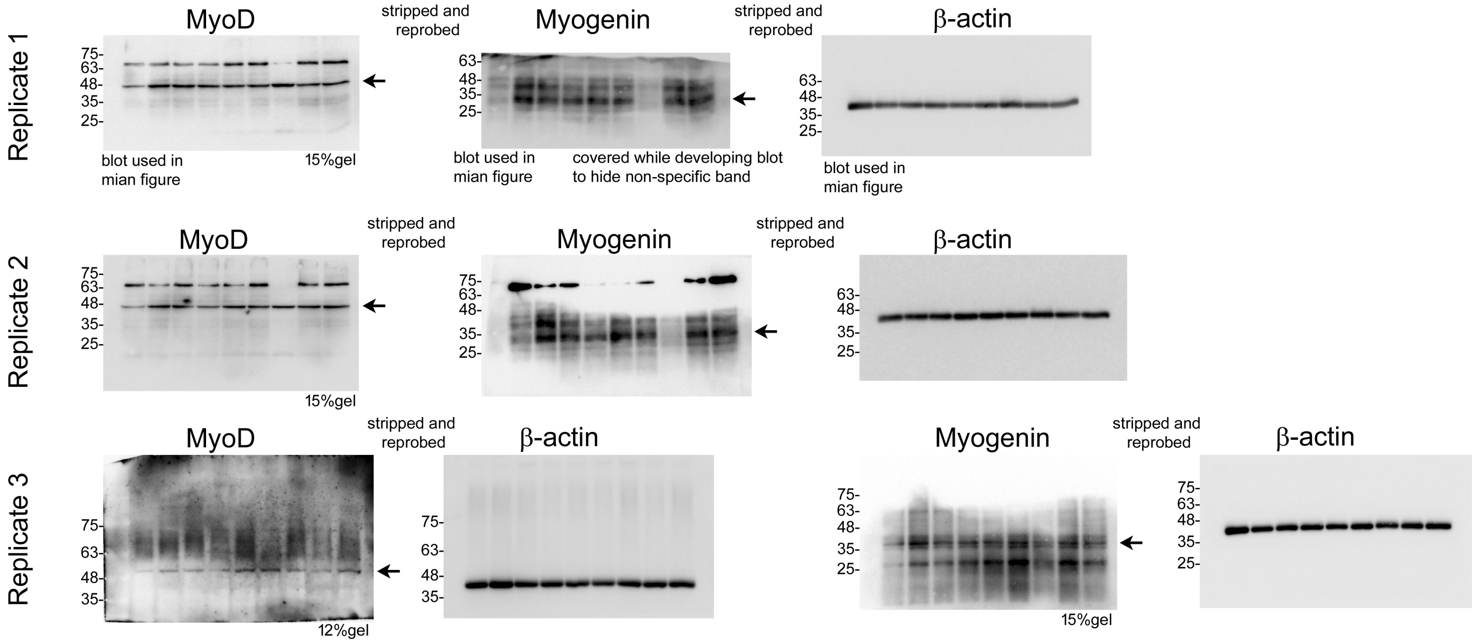

Figure 2C

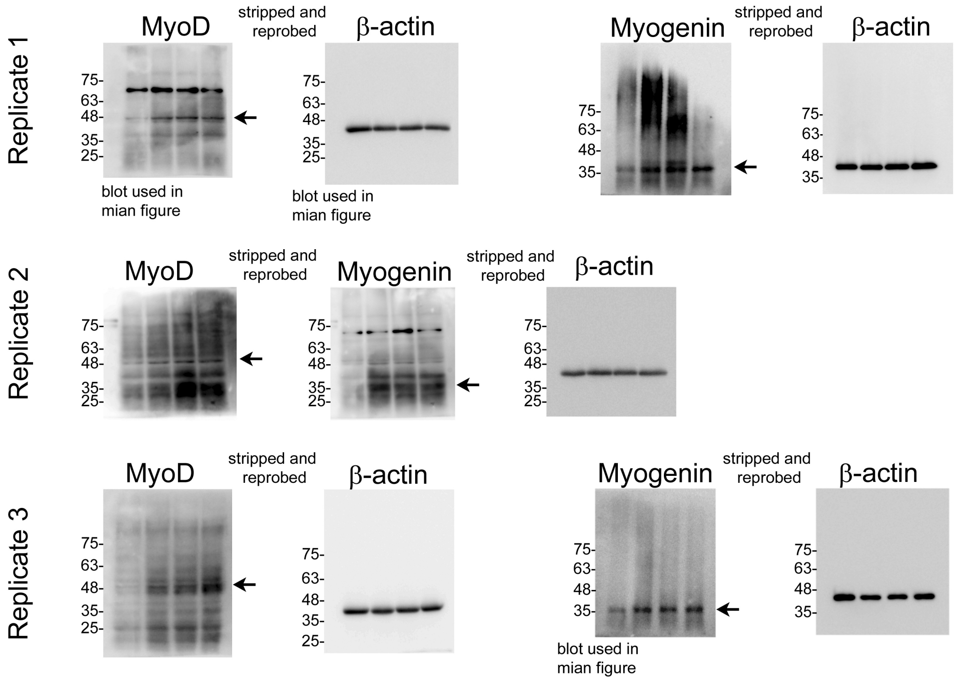

Figure 2E

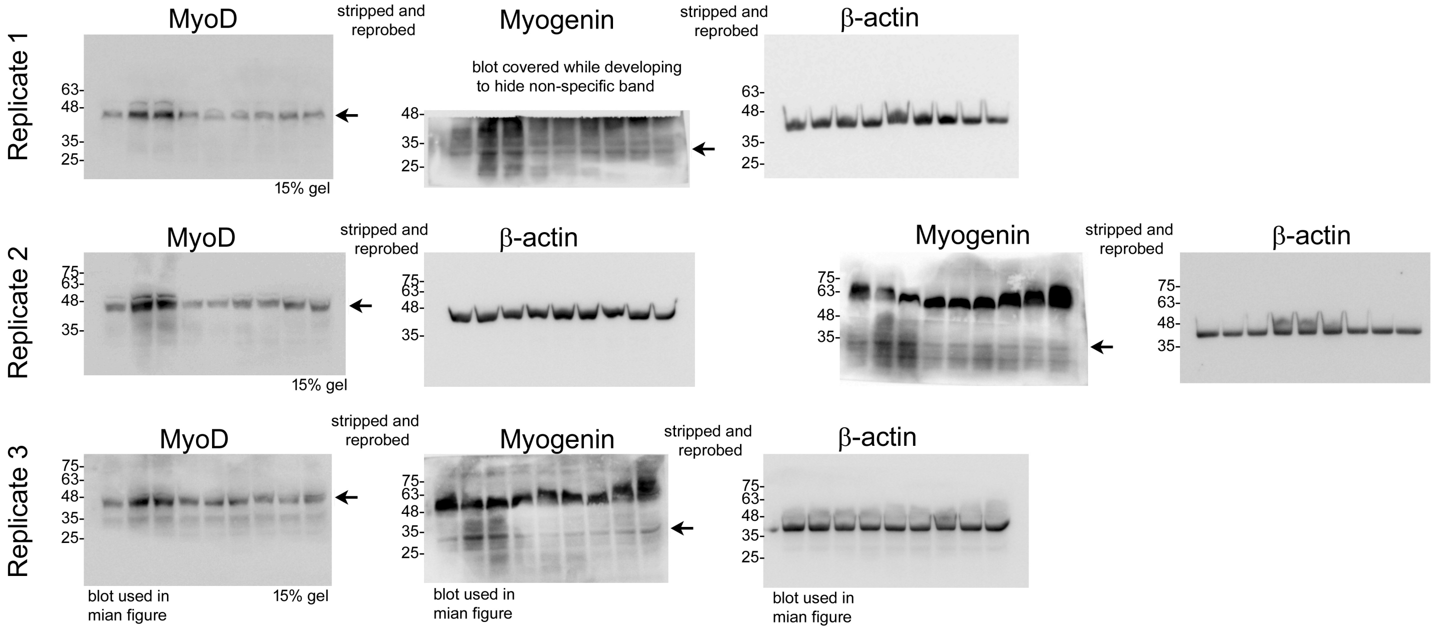

Figure 3

Figure 3B

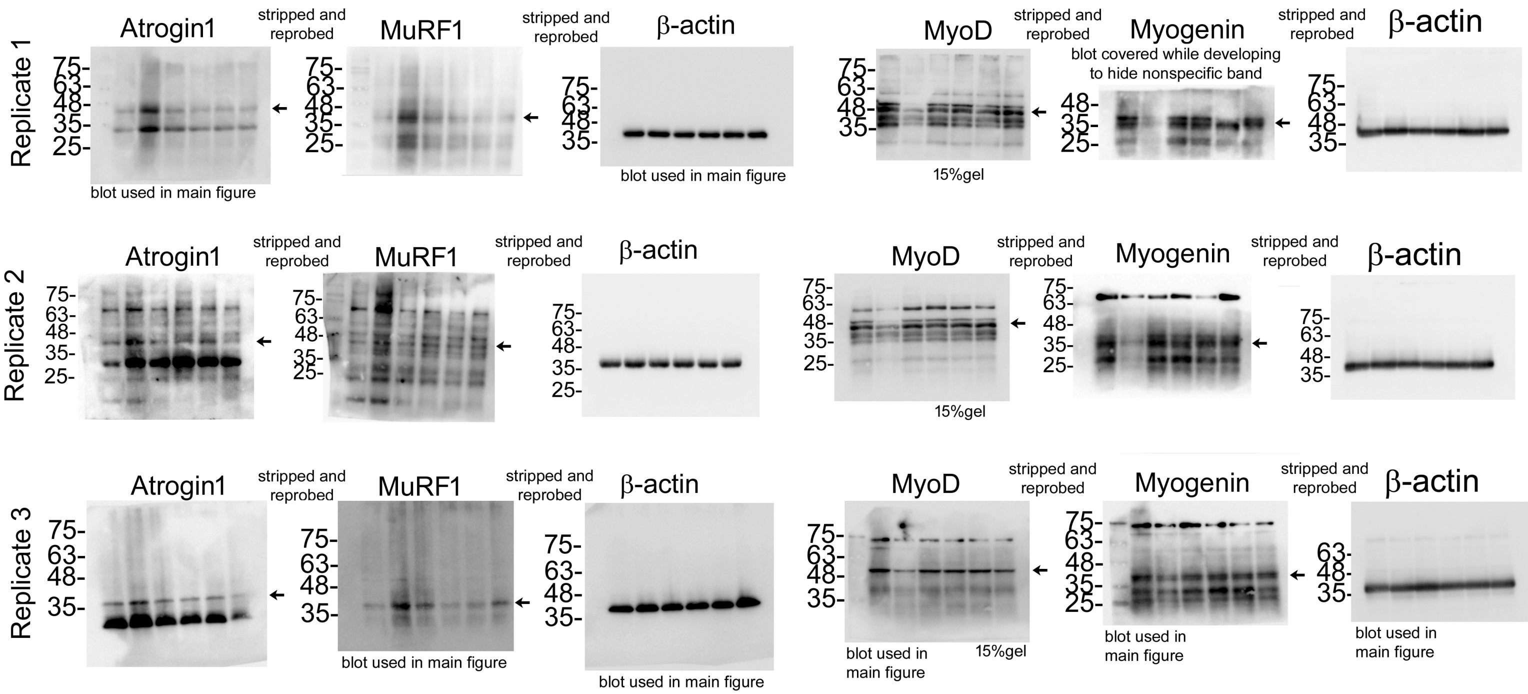

Figure 3C

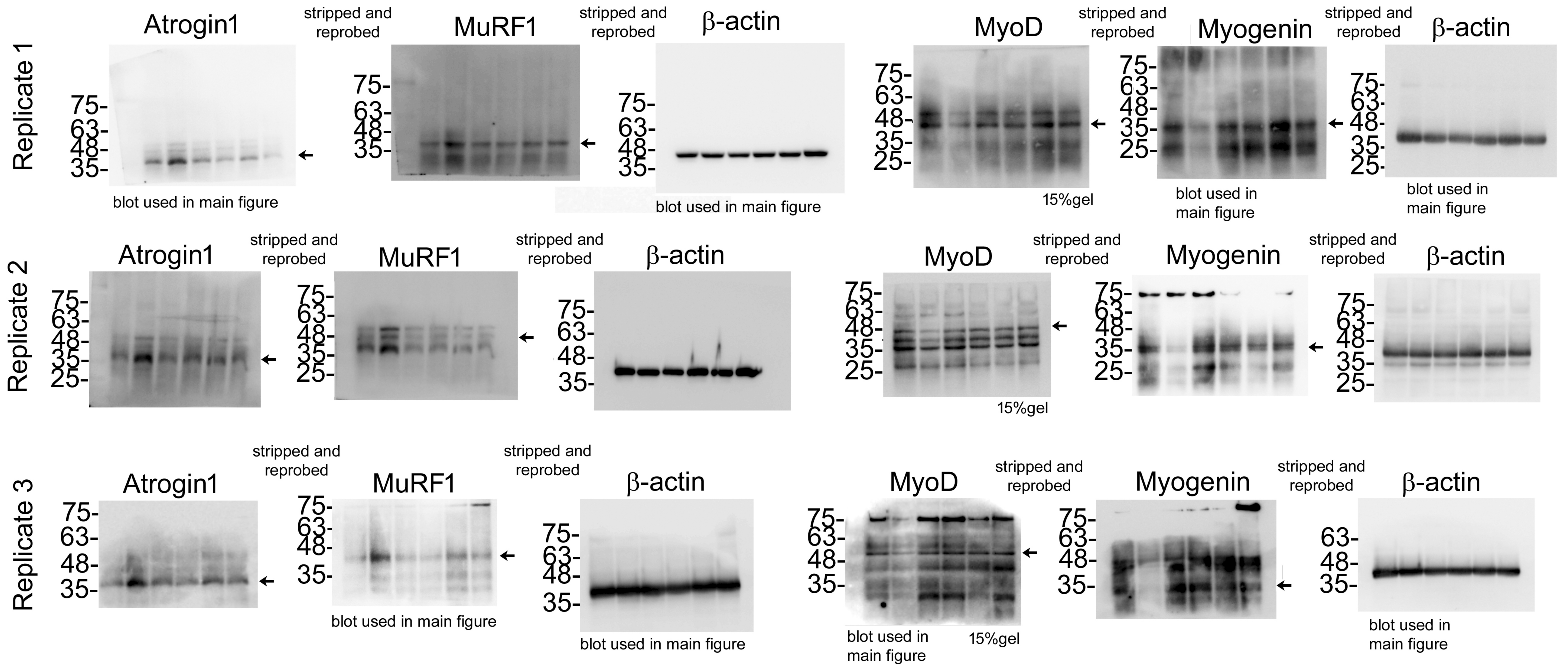

Figure 3F-PBS(-Ca^{2+})

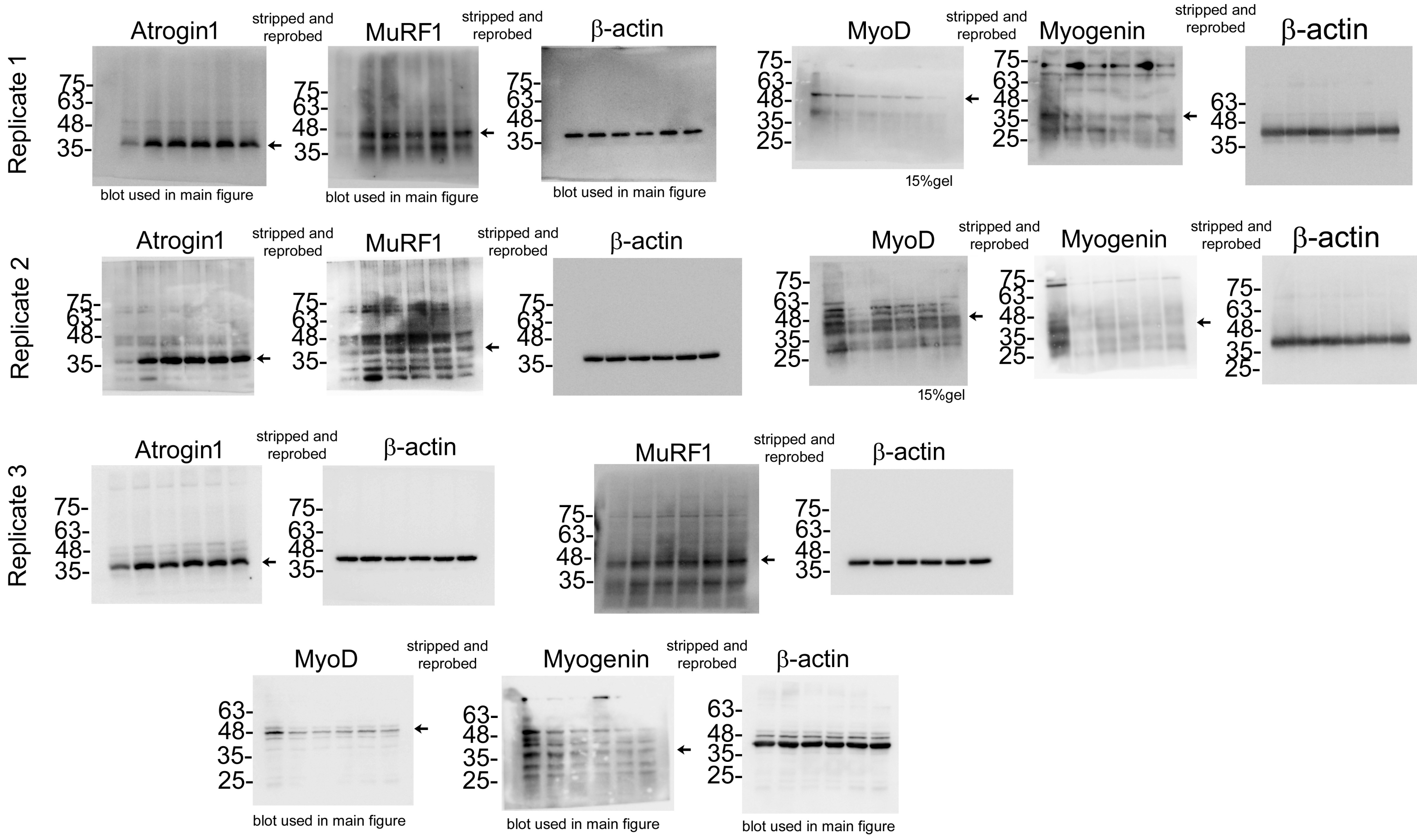

Figure 3F-PBS(+Ca^{2+})

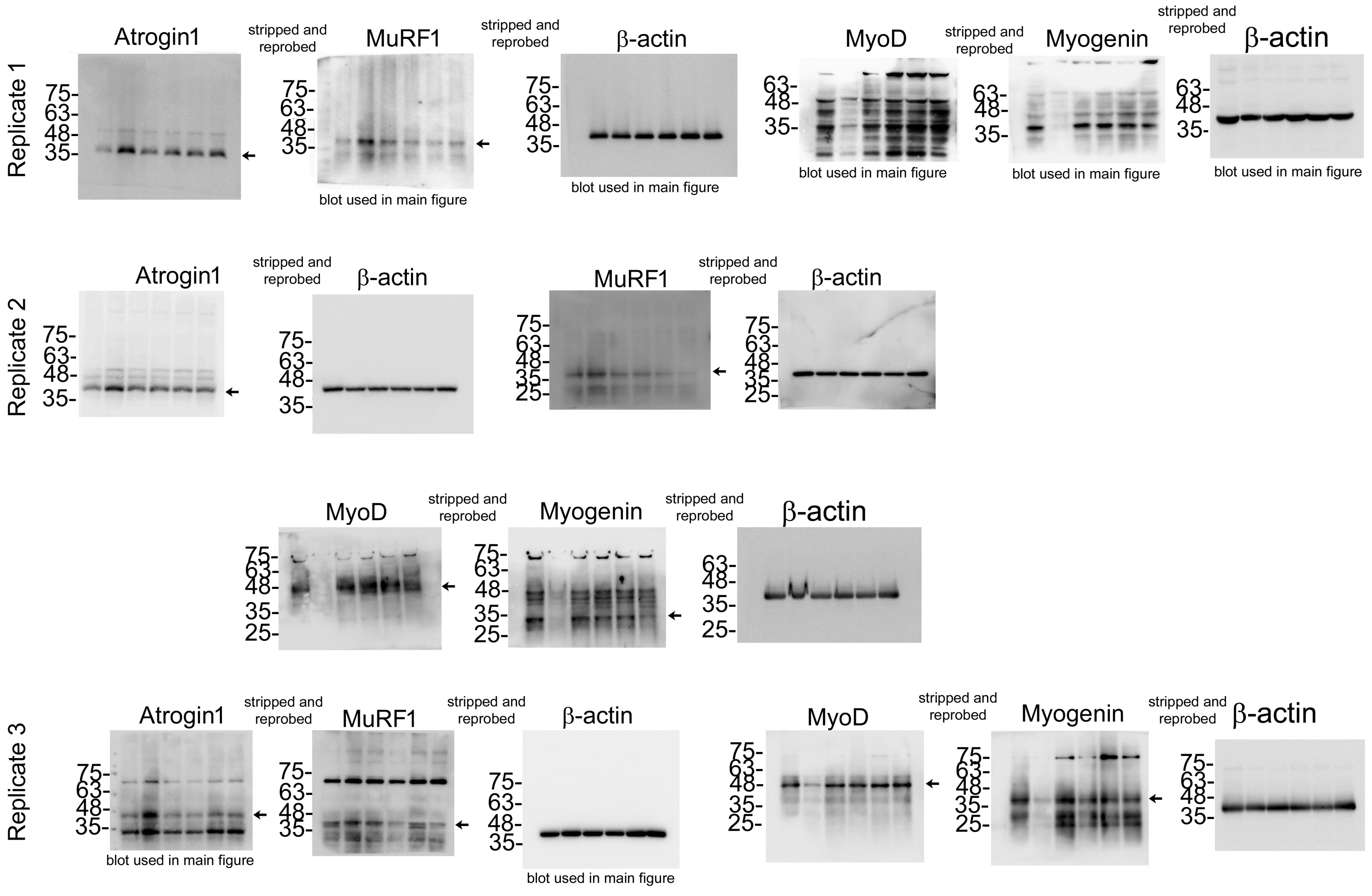

Figure 4

Figure 4A

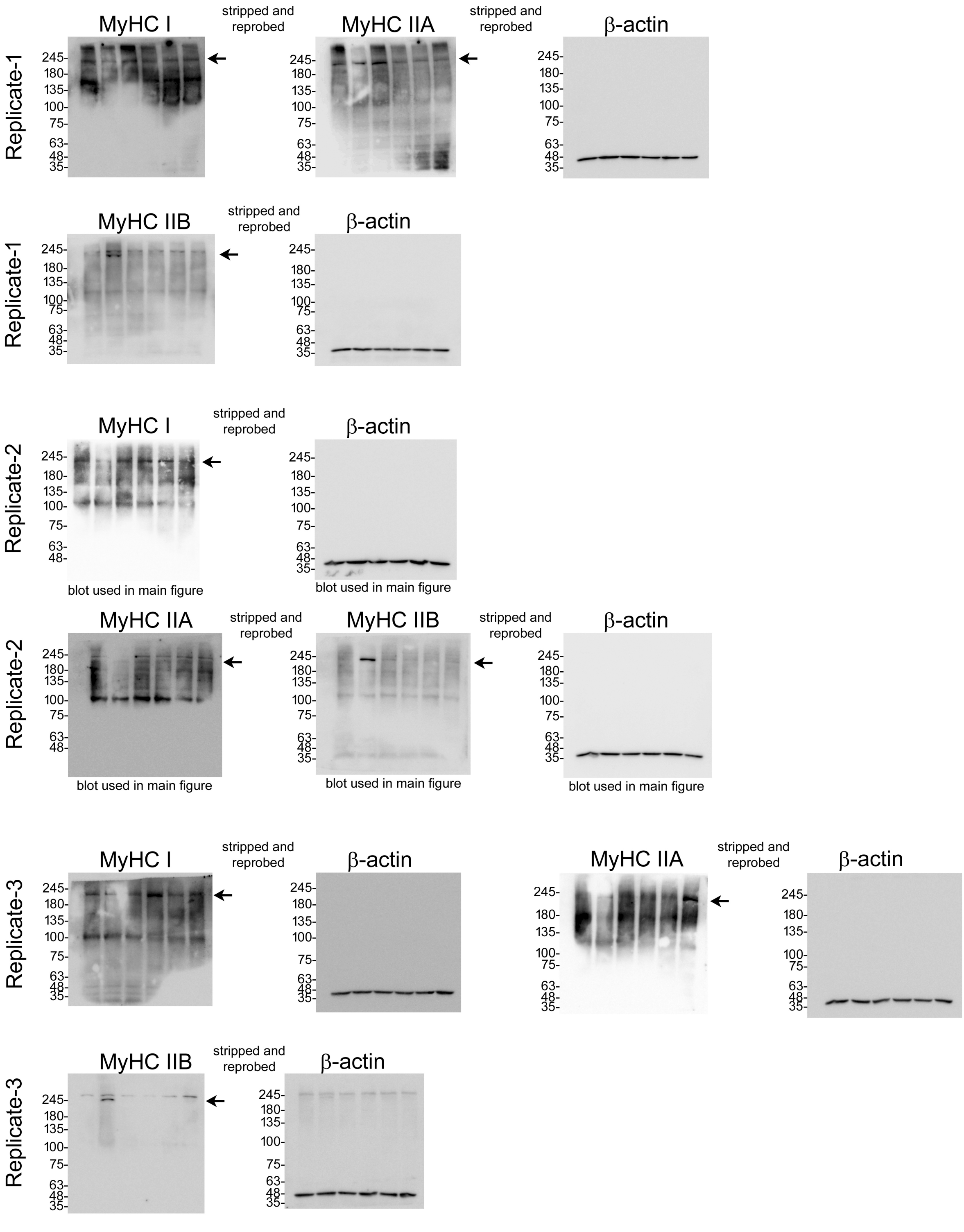

Figure 4D

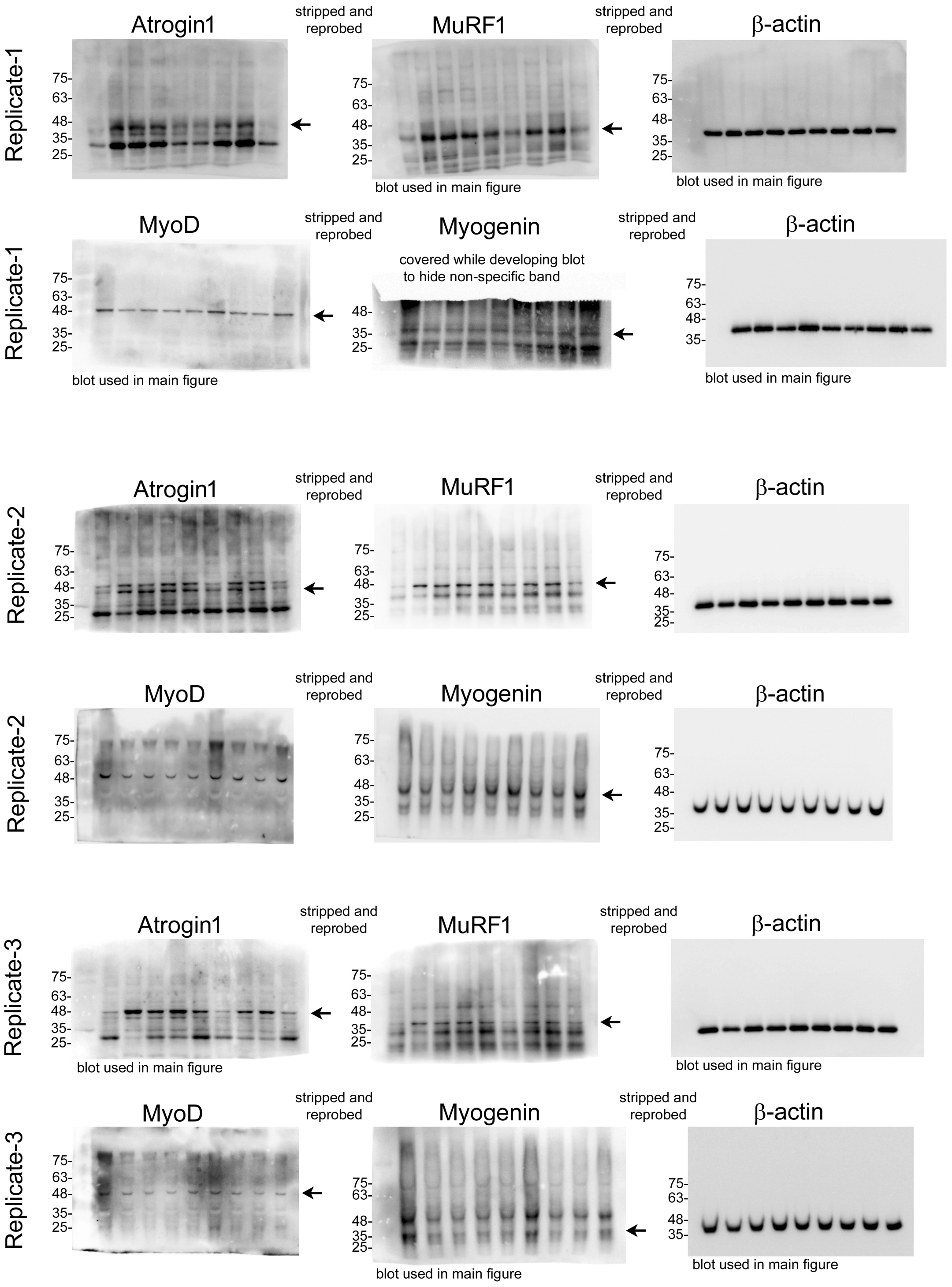
Replicate-1
Atrogin1
stripped and reprobed
MuRF1
stripped and reprobed
β-actin
75-
63-
48-
35-
25-
blot used in main figure
blot used in main figure
Replicate-1
MyoD
stripped and reprobed
Myogenin
stripped and reprobed
β-actin
covered while developing blot to hide non-specific band
blot used in main figure
blot used in main figure
Replicate-2
Atrogin1
stripped and reprobed
MuRF1
stripped and reprobed
β-actin
Replicate-2
MyoD
stripped and reprobed
Myogenin
stripped and reprobed
β-actin
Replicate-3
Atrogin1
stripped and reprobed
MuRF1
stripped and reprobed
β-actin
blot used in main figure
blot used in main figure
Replicate-3
MyoD
stripped and reprobed
Myogenin
stripped and reprobed
β-actin
blot used in main figure
blot used in main figure

Figure 5C-Part-A

Figure 5C- full blots of animals 1 - 3

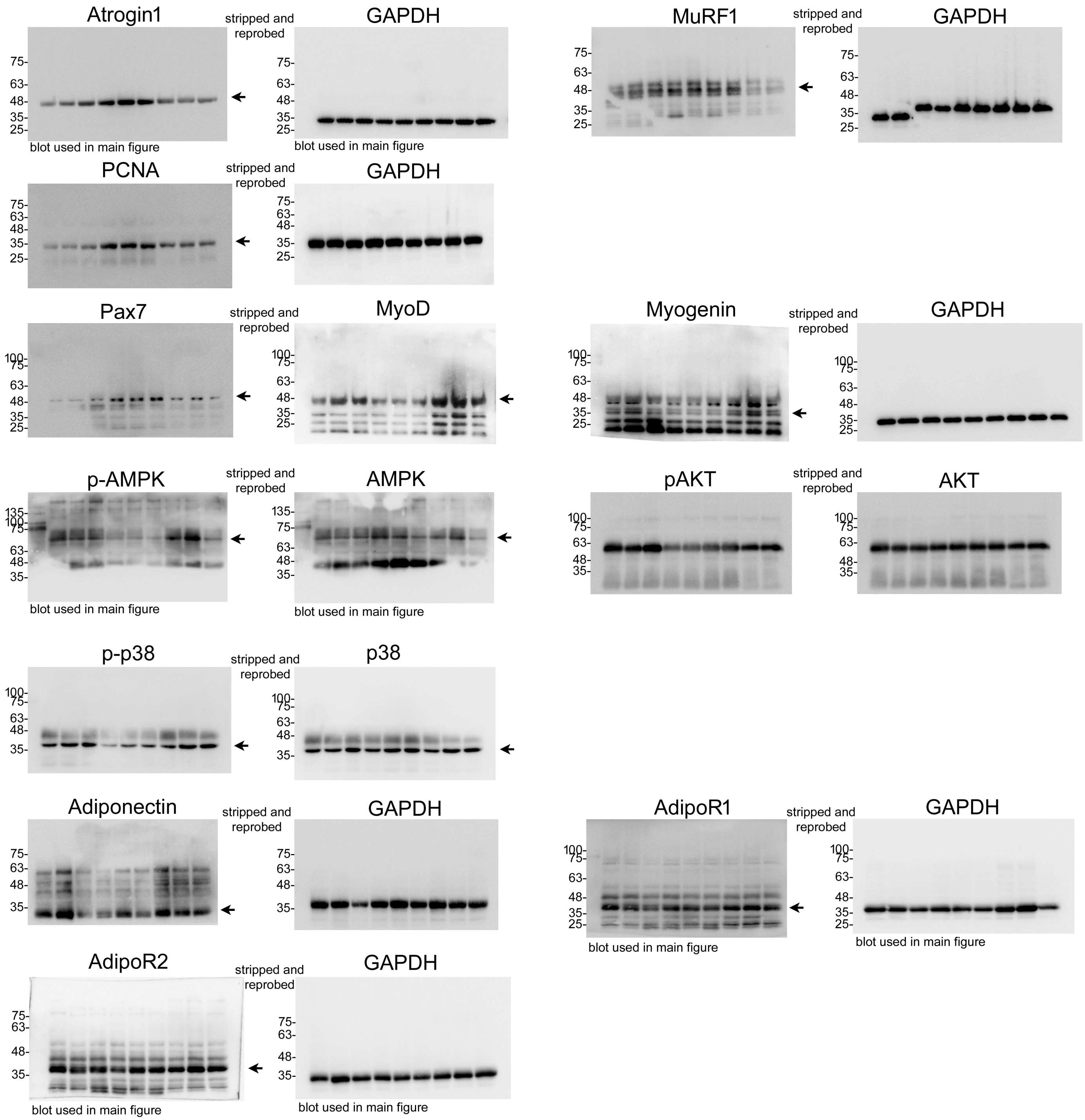

Figure 5C-Part-B

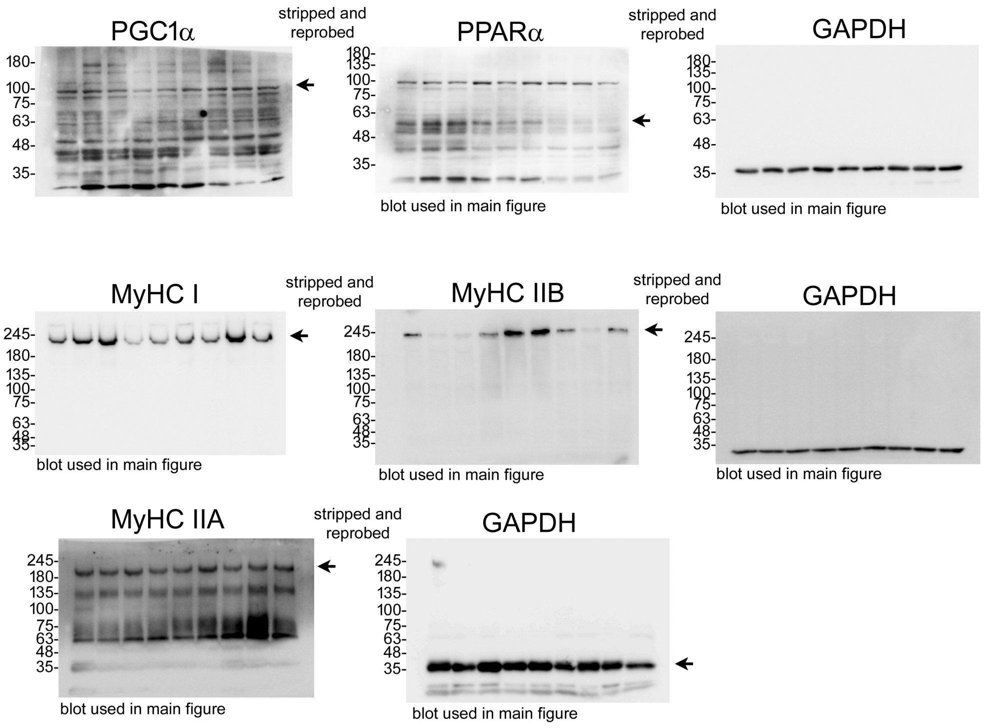

Figure 5C - full blots of animals 4 - 6

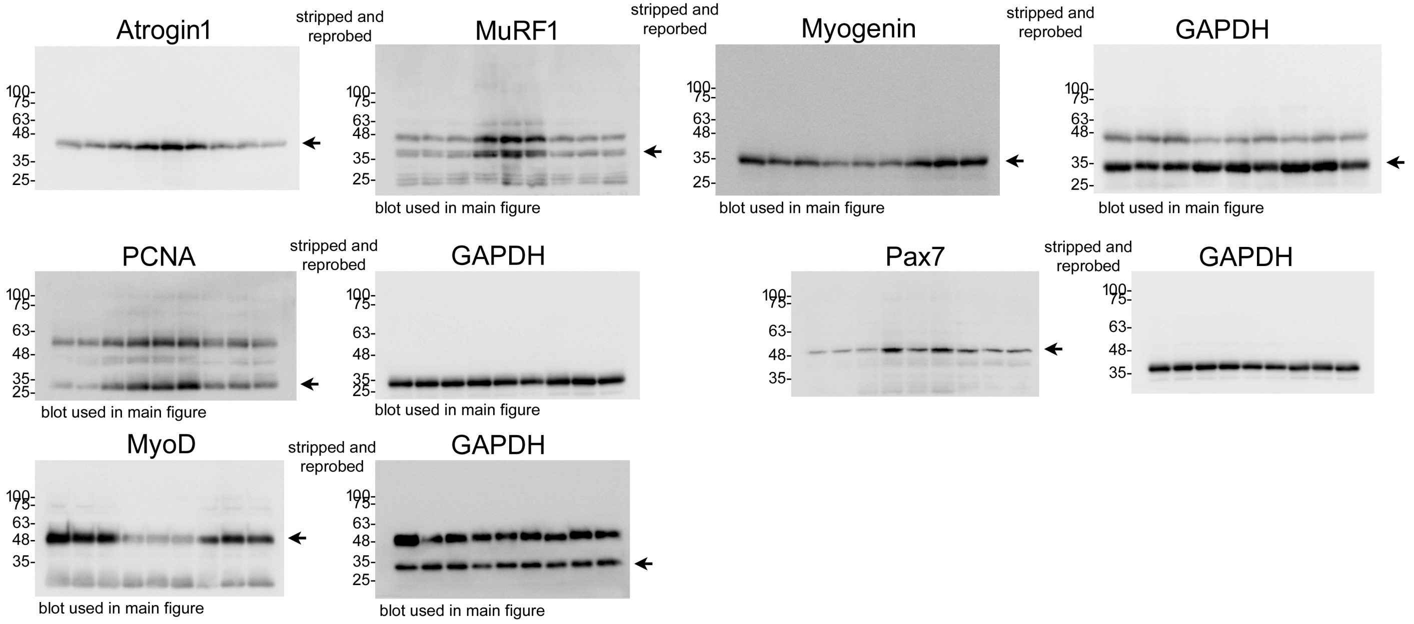

Figure 5C-Part-C

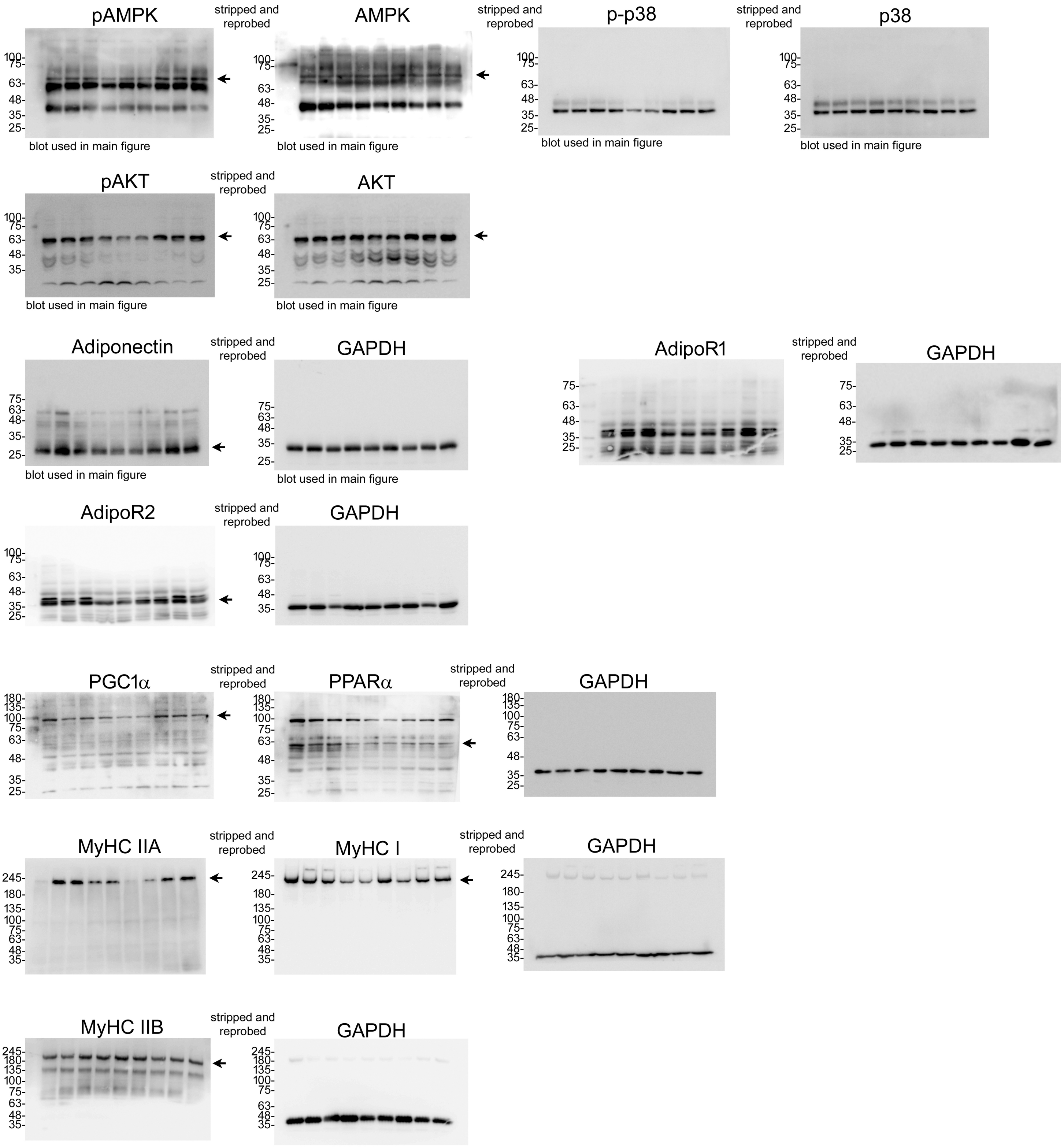

Figure 6D

Figure 6D-3d denervation

3 day denervation- animals 1-3

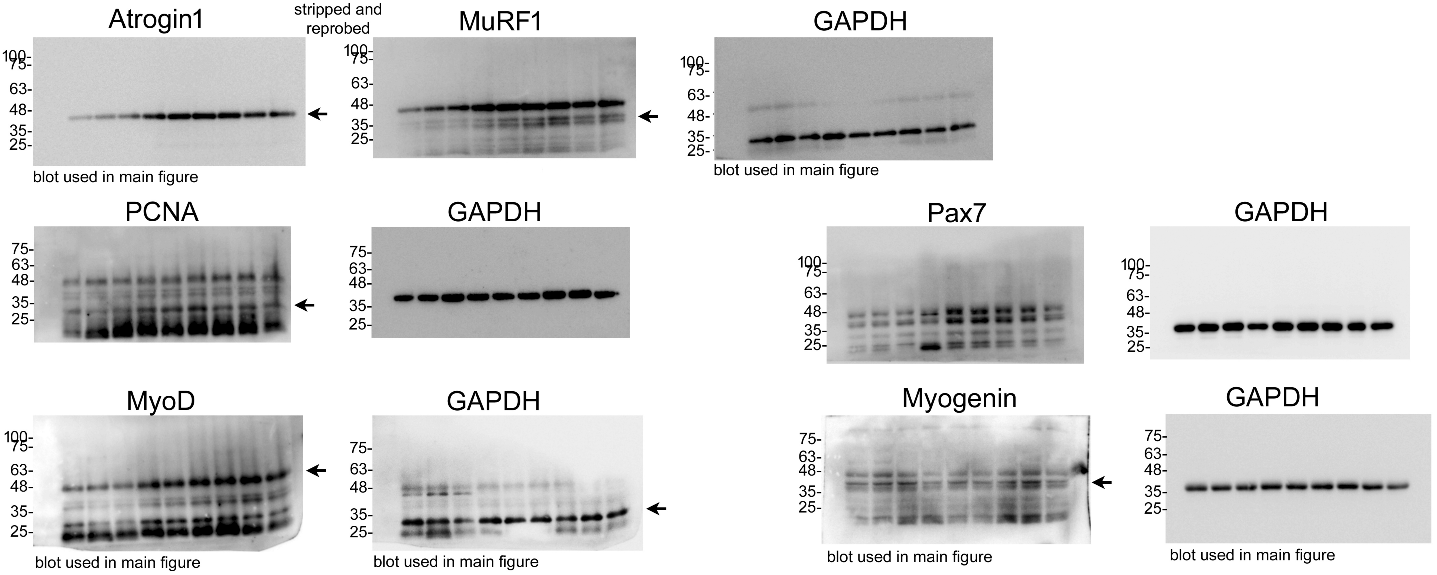

3 day denervation- animals 4-6

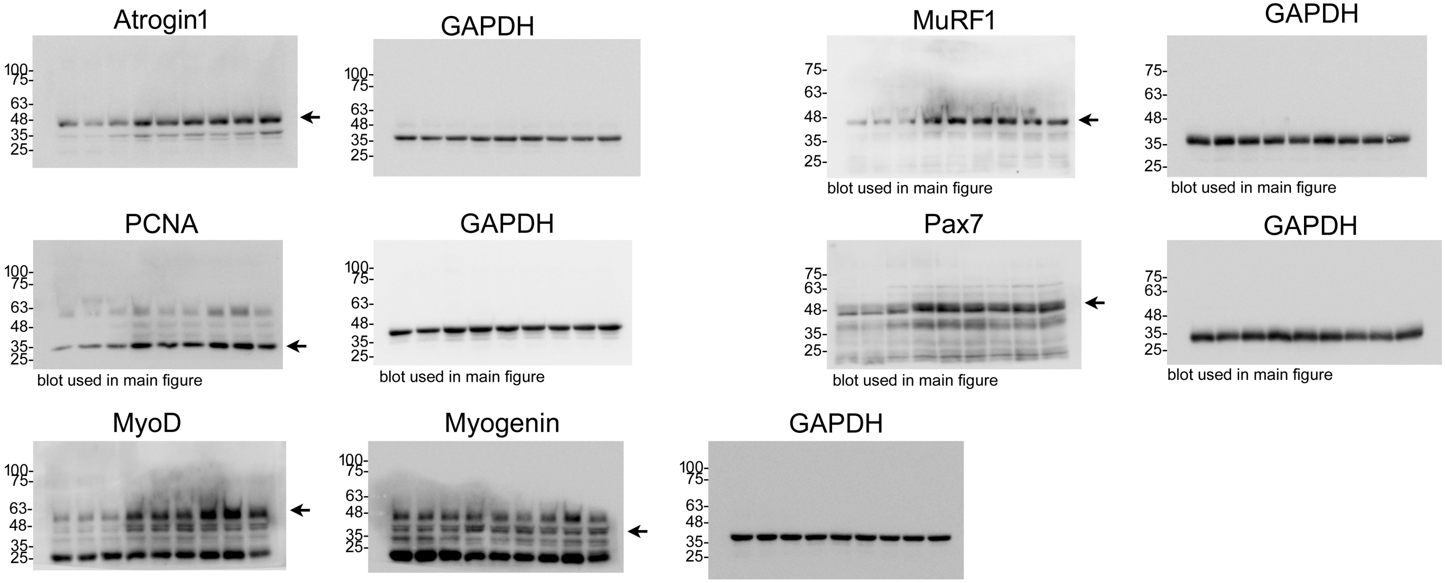

Figure 6E-Part-A

7 day denervation- animals 2,3 of sham and den; den+1709: animals 1-3

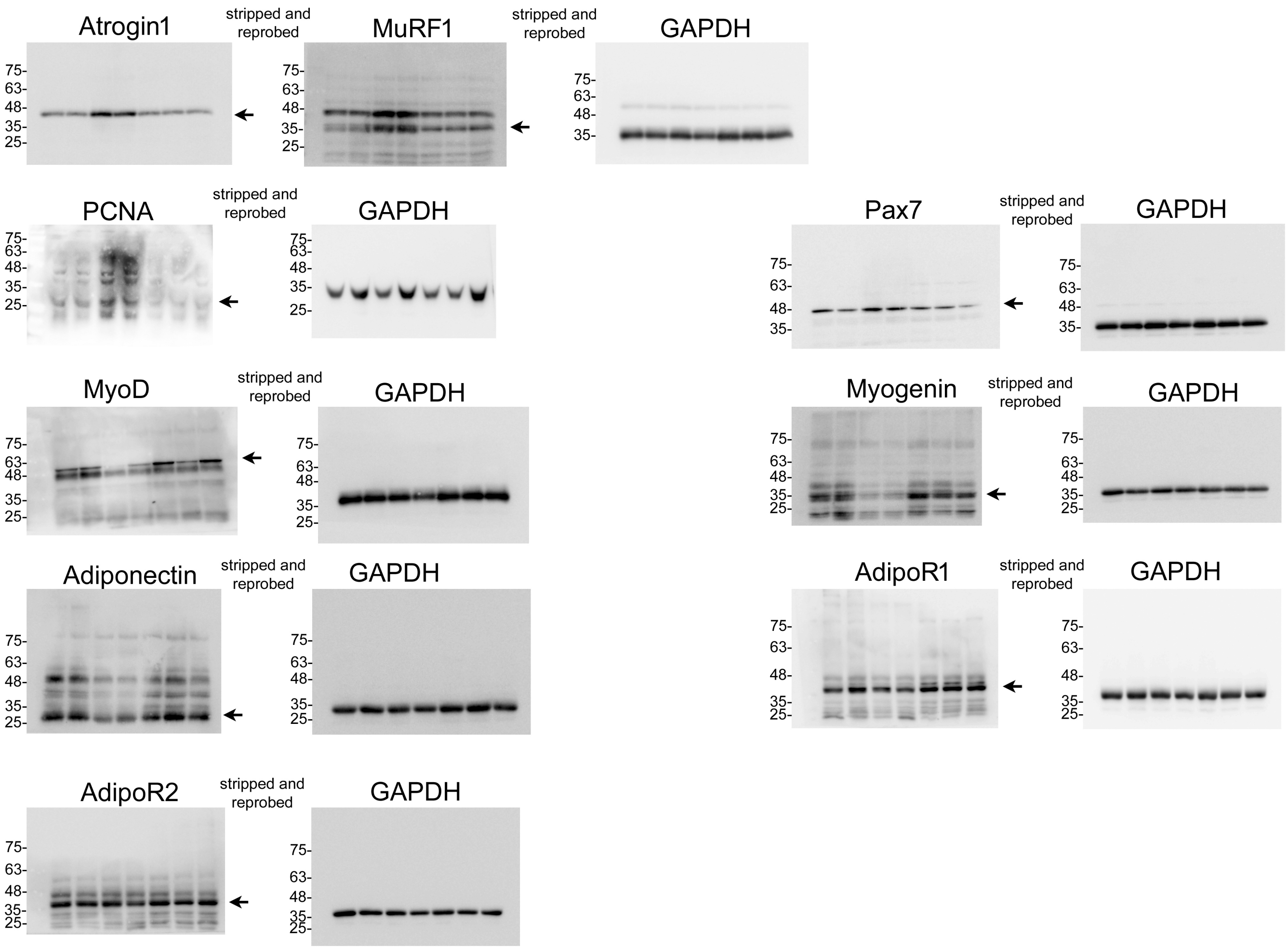

Figure 6E-Part-B

7 day denervation- animals 4-6

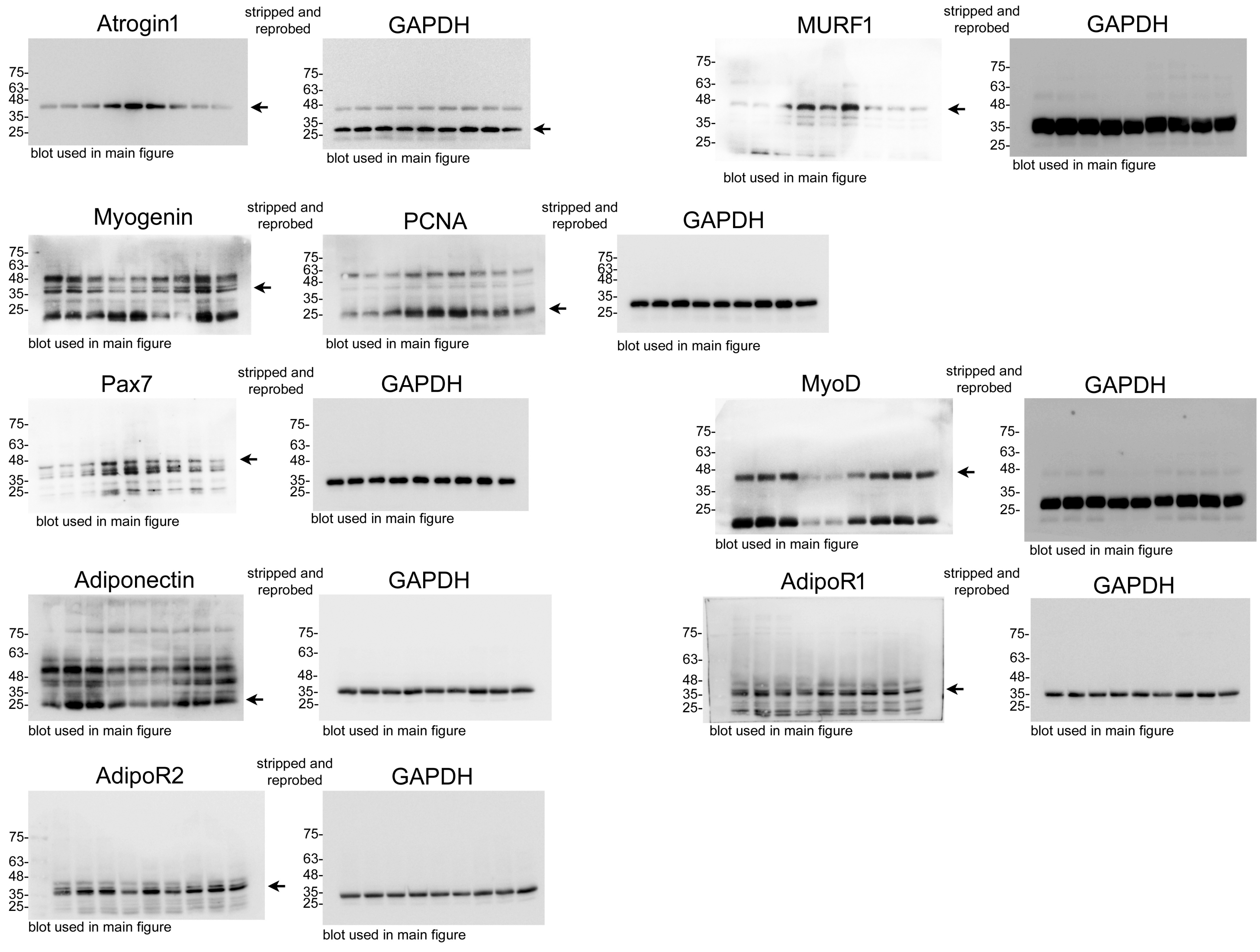

Supplementary Figure S1-Full Blots

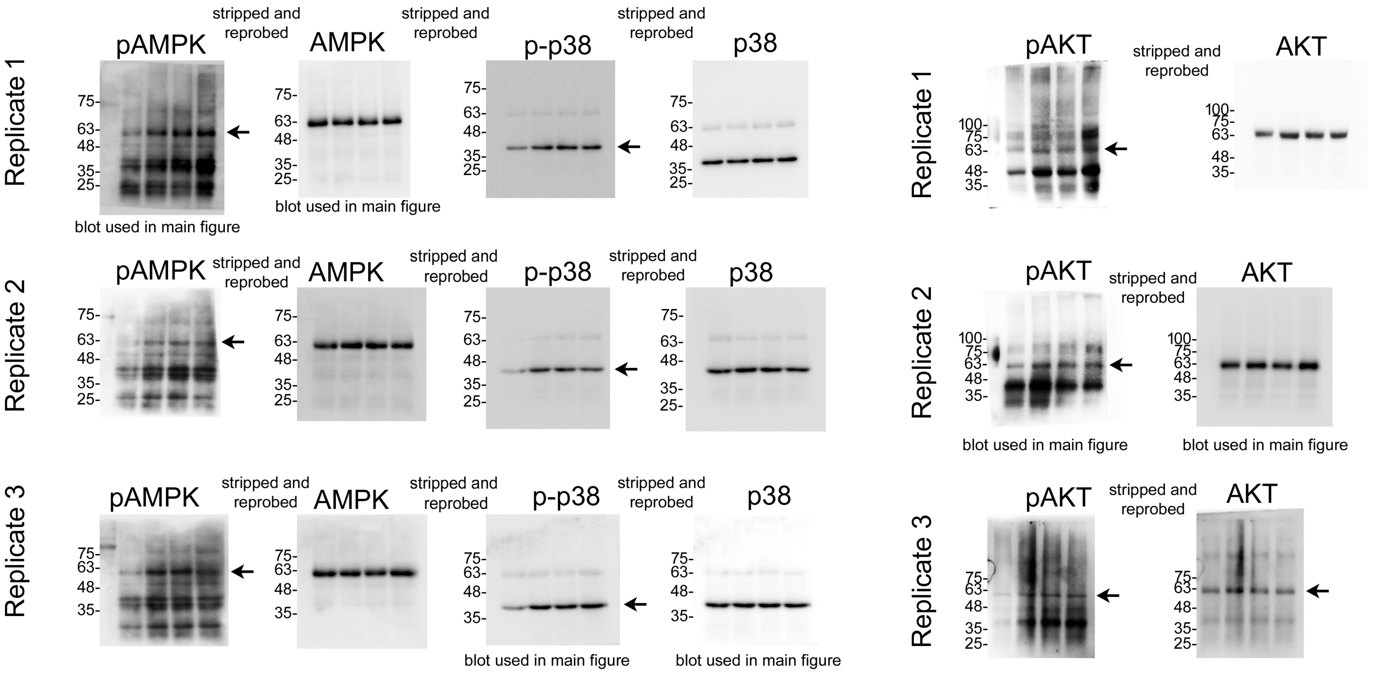

Supplementary Figure S2A-KD(set-2)-10min-Full Blots

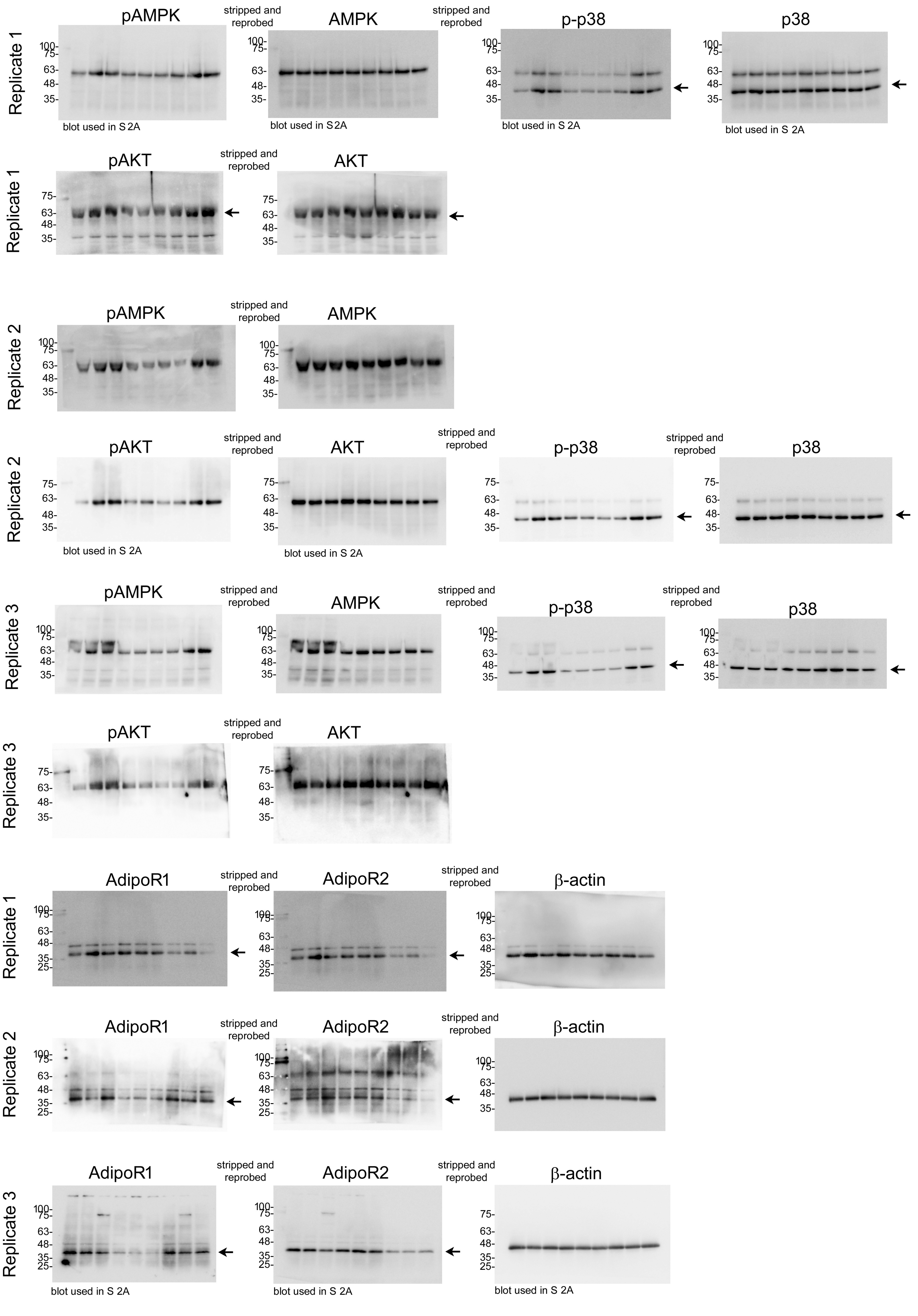

Supplementary Figure S2B-KD(set-2)-24h-Full Blots

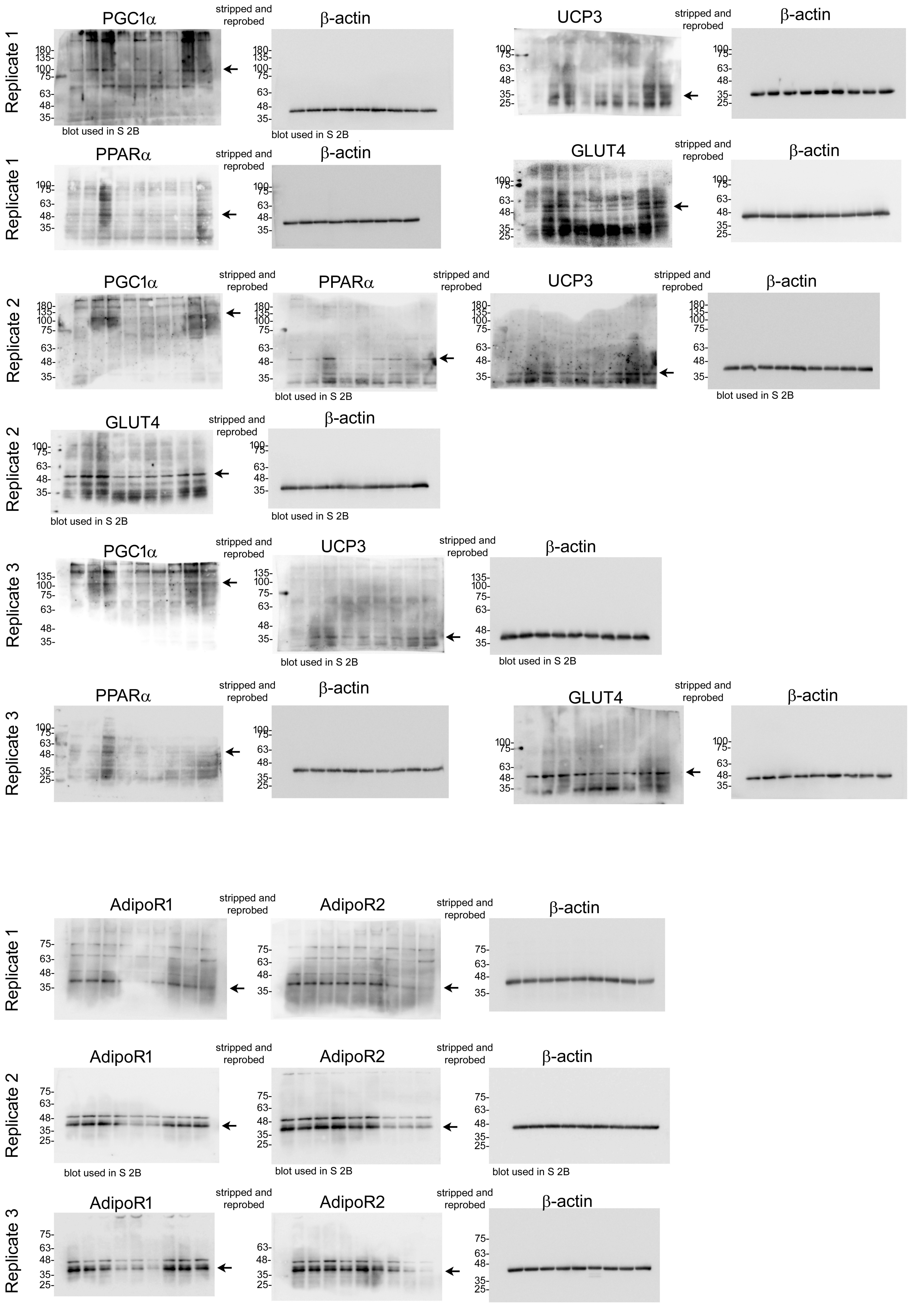

Supplementary Figure S8-Full Blots

7d V (n=5) and 7d 1709 (n=6)

all following blots used as representatives in Supplementary Figure S8

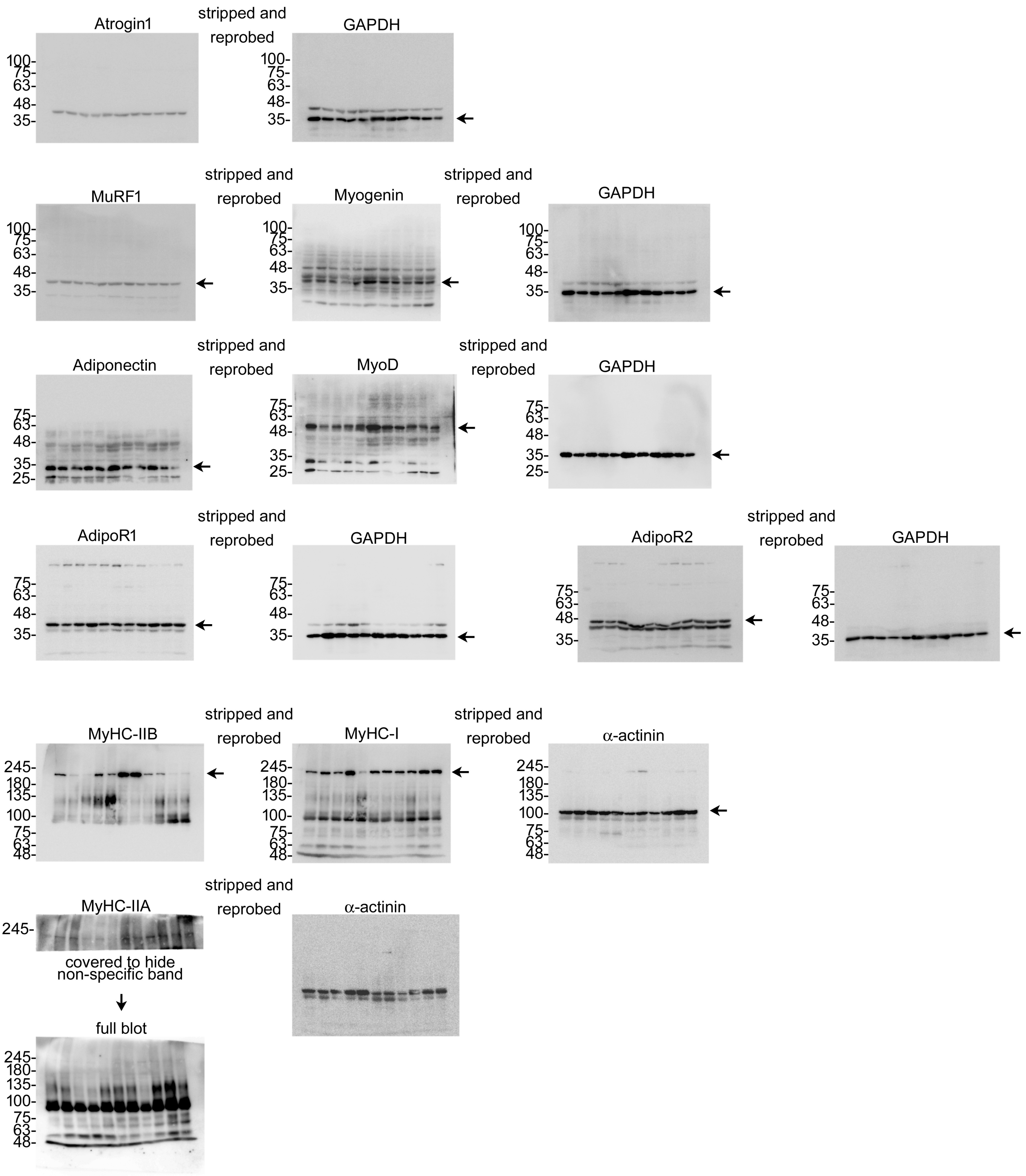

Supplement: Supplementary file 2 — Data S1: Supporting Information. [file JCSM-17-e70328-s002.pdf]
